# Early disruption of photoreceptor cell architecture and loss of vision in a humanized pig model of usher syndromes

**DOI:** 10.15252/emmm.202114817

**Published:** 2022-03-07

**Authors:** Sophia Grotz, Jessica Schäfer, Kirsten A Wunderlich, Zdenka Ellederova, Hannah Auch, Andrea Bähr, Petra Runa‐Vochozkova, Janet Fadl, Vanessa Arnold, Taras Ardan, Miroslav Veith, Gianluca Santamaria, Georg Dhom, Wolfgang Hitzl, Barbara Kessler, Christian Eckardt, Joshua Klein, Anna Brymova, Joshua Linnert, Mayuko Kurome, Valeri Zakharchenko, Andrea Fischer, Andreas Blutke, Anna Döring, Stepanka Suchankova, Jiri Popelar, Eduardo Rodríguez‐Bocanegra, Julia Dlugaiczyk, Hans Straka, Helen May‐Simera, Weiwei Wang, Karl‐Ludwig Laugwitz, Luk H Vandenberghe, Eckhard Wolf, Kerstin Nagel‐Wolfrum, Tobias Peters, Jan Motlik, M Dominik Fischer, Uwe Wolfrum, Nikolai Klymiuk

**Affiliations:** ^1^ Chair of Molecular Animal Breeding and Biotechnology LMU Munich Munich Germany; ^2^ Center for Innovative Medical Models LMU Munich Munich Germany; ^3^ Institute of Molecular Physiology Molecular Cell Biology Johannes Gutenberg University (JGU) Mainz Germany; ^4^ Institute of Animal Physiology and Genetics Czech Academy of Science Libechov Czech Republic; ^5^ Large Animal Models in Cardiovascular Research Internal Medical Department I TU Munich Munich Germany; ^6^ Ophthalmology Clinic University Hospital Kralovske Vinohrady Praha Czech Republic; ^7^ Biostatistics and Data Science Paracelsus Medical University Salzburg Austria; ^8^ Veterinary Faculty Small Animal Clinics LMU Munich Munich Germany; ^9^ Institute of Experimental Genetics Helmholtz Center Munich Neuherberg Germany; ^10^ Institute of Experimental Medicine Czech Academy of Science Prague Czech Republic; ^11^ Centre for Ophthalmology University Eye Hospital University Hospital Tübingen Tübingen Germany; ^12^ Institute for Ophthalmic Research Centre for Ophthalmology University Hospital Tübingen Tübingen Germany; ^13^ Department of Otorhinolaryngology, Head and Neck Surgery University Hospital Zurich (USZ) University of Zurich Zurich Switzerland; ^14^ Faculty of Biology LMU Munich Planegg Germany; ^15^ Institute of Molecular Physiology Cilia Biology JGU Mainz Mainz Germany; ^16^ Grousbeck Gene Therapy Center Mass Eye and Ear and Harvard Medical School Boston MA USA; ^17^ Institute of Developmental Biology and Neurobiology Johannes Gutenberg University (JGU) Mainz Germany; ^18^ Oxford Eye Hospital Oxford University NHS Foundation Trust Oxford UK; ^19^ Nuffield Laboratory of Ophthalmology NDCN University of Oxford Oxford UK; ^20^ Present address: Department of Physiological Genomics LMU Munich Munich Germany

**Keywords:** gene therapy, impaired vision, photoreceptor morphology, pig model, Usher syndrome, Development, Genetics, Gene Therapy & Genetic Disease

## Abstract

Usher syndrome (USH) is the most common form of monogenic deaf‐blindness. Loss of vision is untreatable and there are no suitable animal models for testing therapeutic strategies of the ocular constituent of USH, so far. By introducing a human mutation into the harmonin‐encoding *USH1C* gene in pigs, we generated the first translational animal model for USH type 1 with characteristic hearing defect, vestibular dysfunction, and visual impairment. Changes in photoreceptor architecture, quantitative motion analysis, and electroretinography were characteristics of the reduced retinal virtue in USH1C pigs. Fibroblasts from USH1C pigs or USH1C patients showed significantly elongated primary cilia, confirming USH as a true and general ciliopathy. Primary cells also proved their capacity for assessing the therapeutic potential of CRISPR/Cas‐mediated gene repair or gene therapy *in vitro*. AAV‐based delivery of harmonin into the eye of USH1C pigs indicated therapeutic efficacy *in vivo*.

## Graphical abstract

The full phenotypic spectrum of Usher Syndrome is reflected in a pig model carrying a patient‐specific mutation after partial humanization of the porcine USH1C gene. Retinal function in USH1C pigs was improved when harmonin expression was reconstituted by AAV‐mediated gene therapy.

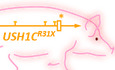

**The paper explained**

**Problem**

Due to the lack of sufficient animal models, the mechanisms of vision loss in Usher syndrome (USH) are incompletely understood and therapies cannot be sufficiently tested. As a consequence, patients inevitably suffer blindness.

**Results**

A pig model for USH reflects the combination of deafness, vestibular dysfunction, and vision loss seen in USH patients. Visual impairment in USH1C pigs, as documented by ophthalmologic examinations and parameterized behavioral tests, is accompanied by morphologic changes in photoreceptor cells. Further, photoreceptor cilia and primary cilia of fibroblasts were consistently elongated in USH1C pigs and facilitated the testing of gene supplementation and gene correction as potential therapeutics. Exploratory gene therapy experiments prove the amelioration of visual function in USH1C pigs.

**Impact**

The USH1C pig is the first animal model for studying the ocular component of the Usher syndrome *in vivo*, at the cellular and the molecular level. Our achievements will promote future research in two relevant ways: (i) reversible changes in primary cilia length make fibroblasts a simple, cheap and robust *in vitro* evaluation tool for testing treatment options in USH. (ii) The consistent multi‐disciplinary examination of the ocular phenotype paves the way for a comprehensive evaluation of vision in pigs and therefore towards a more reliable judgment of pre‐clinical therapy studies in the USH1C pig model *in vivo*.

## Introduction

Usher syndrome (USH) is the most common form of inherited deaf‐blindness in humans (Mathur & Yang, 2015). USH is clinically and genetically heterogeneous, with at least 10 genes assigned to three clinical USH types (Moller *et al*, 1989; Toms *et al*, 2020). The most severe of them is USH1, characterized by profound hearing loss from birth on, vestibular areflexia, and pre‐pubertal onset of retinitis pigmentosa (RP). In patients, the congenital sensorineural hearing impairment can be compensated with cochlear implants, but no therapeutic option is presently available for the ocular disease constituent. Thus, most USH cases lead to severe visual impairment and blindness over time, adding a substantial psychological component to the clinical symptoms (Lonborg‐Moller *et al*, 2020).

Development of therapies and understanding of USH pathogenesis in the retina is arduous as existing rodent models for USH reflect the human deficits in the inner ear, but show only a very mild, if any, retinal phenotype (Lentz *et al*, 2007; Williams, 2008; El‐Amraoui & Petit, 2014). This has been attributed to substantial differences between mouse and human in anatomy and cellular composition of the retina and stimulated discussion about alternative model species. Pigs reflect structure and function of the human eye much better than mice as they have a similar size, a comparable rod/cone ratio and a cone‐rich region, the so‐called visual streak (Chandler *et al*, 1999; Hendrickson & Hicks, 2002). Specifically, calyceal processes (CP), cellular extravaginations at the transition of the inner segment (IS) to the outer segment (OS) of photoreceptor cells (PRC) appear in many species, including human and pig, but not rodents (Wolfrum *et al*, 2010; Sahly *et al*, 2012; El‐Amraoui & Petit, 2014). Various pig models have been described for retinal degeneration, with the majority of them induced by compounds or light (Li *et al*, 2009; Wang *et al*, 2011; Monés *et al*, 2016) or additive transgenesis (Petters *et al*, 1997; Sommer *et al*, 2011; Ross *et al*, 2012; Kostic *et al*, 2013). As the latter do not truly reflect the genetic constellation in patients, the phenotype was, however, difficult to interpret and limited the utility of these models.

We therefore hypothesized that introducing a patient‐specific mutation into a partially humanized *USH1C* gene would facilitate a better genotype–phenotype correlation of an USH1C pig model. *USH1C* comprises 28 exons with alternative splicing giving rise to numerous transcript variants (Zwaenepoel *et al*, 2001; preprint: Nagel‐Wolfrum *et al*, 2021). According to their domain composition, the transcripts are classified into the three protein subgroups harmonin_a, _b, and _c (Verpy *et al*, 2000; Sahly *et al*, 2012). Most characteristic for harmonin are several PDZ domains (Kim and Sheng 2004). N‐harm and CC (coiled‐coil) domains are further integral constituents of all harmonin variants, while only harmonin_b comprises a second CC as well as a PST (proline‐serine‐threonine rich) domain (Verpy *et al*, 2000; Boeda *et al*, 2002; preprint: Nagel‐Wolfrum *et al*, 2021). A gene therapy approach in the inner ear of a murine *Ush1c* model demonstrated the physiological relevance of distinct isoforms: harmonin_a1 appeared sufficient for restoring the vestibular system while only harmonin_b1 corrected deficits in the auditory function (Pan *et al*, 2017). The specific relevance of harmonin_b variants for hearing is also illustrated by mutations in the CC2 or the PST domain, causing autosomal recessive deafness (DFNB18A) rather than USH in human patients (Ouyang *et al*, 2002) or in a naturally occurring deaf‐circler mouse line (Johnson *et al*, 2003). In contrast, USH‐causing mutations are located in the common N‐harm, PDZ, or CC1 domains (Fuster‐Garcia *et al*, 2021).

The components of the USH1 and USH2 interactome are the main, but not exclusive interaction partners of harmonin (Reiners & Wolfrum, 2006; Mathur & Yang, 2015). The mutual interplay of USH proteins and the molecular properties of harmonin, however, remain elusive. In the inner ear, harmonin serves in the formation and functionality of the interstereociliary links in the bundles protruding from cochlear hair cells (El‐Amraoui & Petit, 2005; Richardson *et al*, 2011). Harmonin is also consistently localized to the ribbon synapses of hair cells and PRCs (Reiners *et al*, 2005; Williams *et al*, 2009a; Gregory *et al*, 2011), whereas data on harmonin abundance in other compartments of the retina are contradictory in rodents (Reiners *et al*, 2003, 2005; Williams *et al*, 2009a; Sahly *et al*, 2012). In species with pronounced CP structures in PRC such as amphibians or primates, however, USH proteins, including harmonin, seem to co‐localize with CP or, at least, appear in the transition zone of IS to OS (Sahly *et al*, 2012; Schietroma *et al*, 2017). Very recently, we detected harmonin also in the OS of rods, in Müller glia cells (MGC), and in the outer limiting membrane of the human retina at the adhesive junctions between MGC and PRC (preprint: Nagel‐Wolfrum *et al*, 2021).

Based on these considerations, we designed and generated an USH1C pig model, carrying the deleterious c.C91T/p.R31X non‐sense mutation after humanization of exon 2 and its surrounding intronic sequences in the porcine *USH1C* gene. For the integration of exon 2 in the major splice variants *USH1C_a*, *_b*, and *_c*, the chosen mutation will affect all relevant *USH1C* transcripts and its disruption was, therefore, proposed to cause the full spectrum of USH. USH1C pigs revealed profound hearing impairment, pronounced vestibular dysfunction, and significant visual deficits. At the cellular level, the retinal phenotype was characterized by an early disruption of OS photosensitive disc architecture, elongated connecting cilia, and narrower cone synapses. Primary cells from USH1C pigs were sufficient for *in vitro* testing of human‐specific gene repair or gene therapy. The latter approach did also restore retinal function in an *in vivo* pilot study.

## Results

### Genetic modification of the porcine genome by introducing a human segment carrying a patient‐specific mutation into the *USH1C* gene

**Figure 1:**
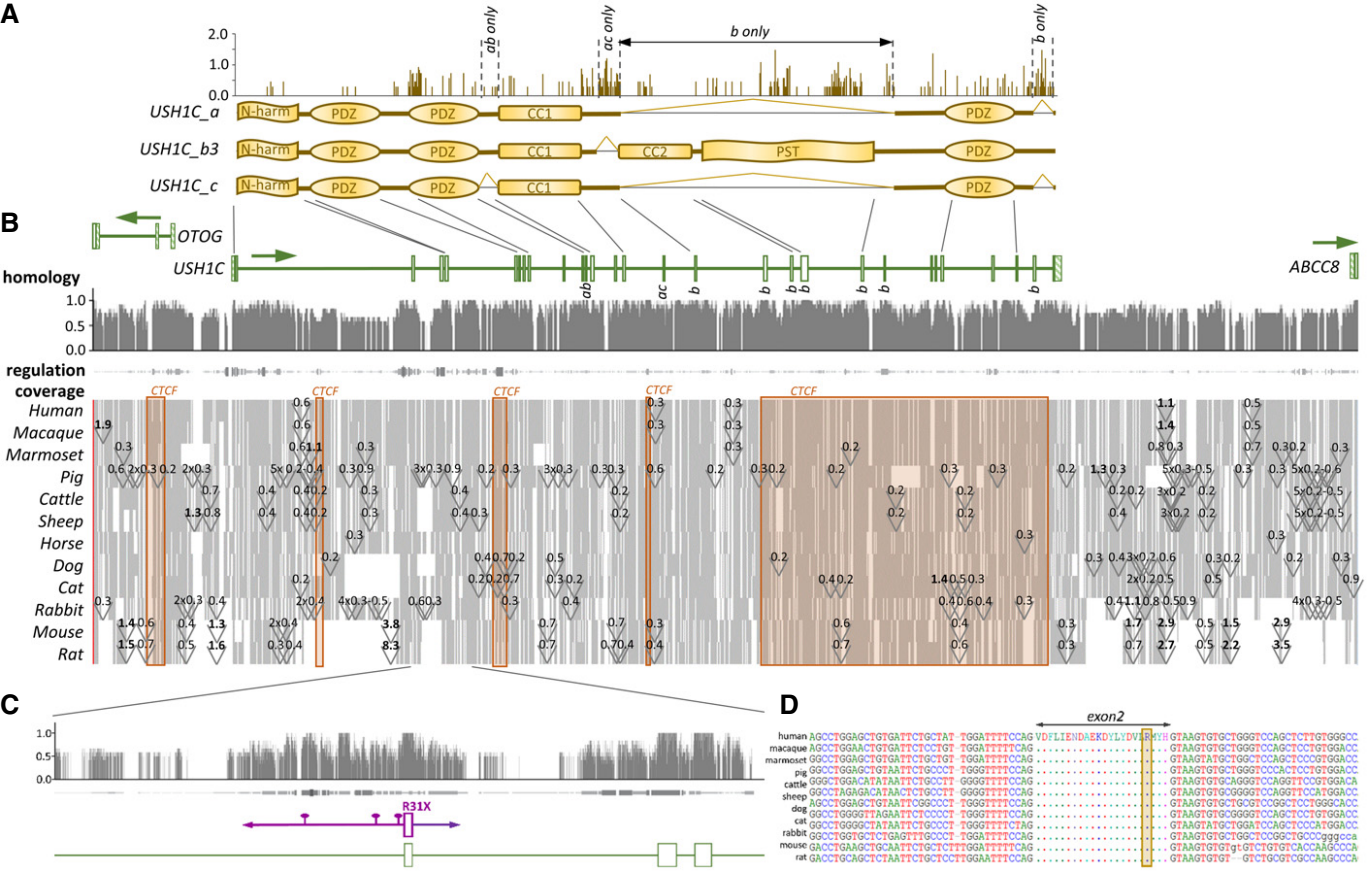
Generation of USH^R31X^ pigs Three main splice variants *_* *a*, *_* *b3*, and *_* *c* have been confirmed for human *USH1C*. According to an Entropy plot (upper panel), the coding regions appear highly conserved among mammalian species with some variability in inter‐domain segments, but also within the _*b3*‐specific PST domain.Homology: A high degree of conservation occurs at the nucleotide level, including exons but also intronic elements. Regulation: High degrees of conservation often correlate with strong indication for transcriptional regulation properties. Thickness and color intensity are according to matches to 6 prediction tools (DNase sensitivity, ChIP‐Chip, FANTOM, GeneHancer, FAIRE, PreMod). Coverage: Most of the *USH1C* gene and its surrounding regions are consistently abundant in the examined species (grey). Gaps (white) appear in all species, but mostly remain small (< 500 bp). Few intronic areas are consistently affected by the introduction of repetitive elements (triangles, with approximate length in kb). Rodents show a high evolutionary turnover, with large gaps or inserts > 1 kb in extended introns and adjacent intergenic regions of *USH1C*. Significant proportions of USH1C are indicated as CTCF/cohesion‐binding regions that appear relevant for chromosomal interactions (orange blocks).Exon 2 and its surrounding intronic sequences constitute a consistent block in the alignment with high degree of sequence conservation, strong evidence for regulatory purposes and a lack of repetitive element insertion. For this reason, the entire segment was humanized (magenta), including the causative c.C91T/p.R31X mutation as well as three patient‐specific SNPs (pins).All examined species show identical amino acid sequence on the protein level and high degrees of conservation at the intronic borders on the nucleotide level. The yellow box indicates R31, the amino acid that is mutated in R31X patients. Source data are available online for this figure.

**Figure EV1:**
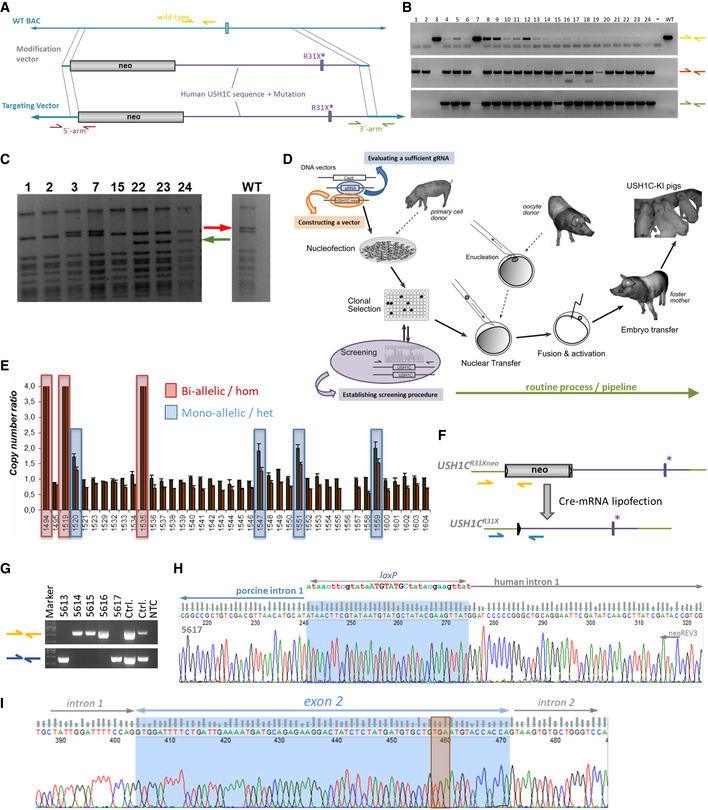
Modification process BAC clone CH242‐515C3 was modified by bacterial recombineering with a modification vector comprising the intended 1.5 kb human USH^R31X^ segment (magenta) and a floxed *neo* selection cassette. Arrows represent primer for endpoint PCR to screen for correct recombination.Correctly modified BAC clones are characterized by negative wild‐type PCR (yellow) and positive PCR spanning across the 5′‐arm (brown) and 3′‐arm (green) of homology.
*Xba*I digest of BAC clones for confirming integrity of the clone and verified the disappearance of an 18,779bp band (red arrow) and the appearance of a 14,194 bp band (green arrow) and a 5,020 bp band (too faint for detection).Modified BAC vectors were co‐transfected with plasmids expressing Cas9 and gRNA4 into pig cells. Clonal selection for neomycin‐resistance delivered single cell clones (SSCs) which were expanded for screening and subsequent usage in SCNT.SSCs were screened for modification by a qPCR‐based loss‐of‐wild‐type‐allele approach. Inverse copy number ratios of the *USH1C* locus vs reference sites in the *POU5F1* and *NANOG* genes indicated SSCs with bi‐allelic (red boxes) and mono‐allelic (blue boxes) modification. Each qPCR was run in a duplicate, facilitating the calculation of 4 quotients of reference site : target site copy numbers for each sample. The mean value of these quotients are given ± SD. For ensuring characterization of SSCs, 2 independent reference sites were used and SSCs that appeared modified in the first screening were used in an independent experiment for confirmation.Before SCNT, SSCs were lipofected with Cre‐encoding mRNA to remove the *neo* selection cassette. Primer sets were designed for discriminating founder animals with (yellow arrows) and without (blue arrows) *neo*.Representative genotyping of a founder litter, indicating that animals 5613 and 5617 have sufficiently excised *neo*. For controls (Ctrl.), WT genomic DNA was mixed with modified BAC for the neo‐PCR (yellow) and mixed with a modified BAC that had been treated with Cre for the delta‐neo PCR (blue).Representative electropherogram of PCR sequencing, confirming correct excision of *neo* in 5617. As a consequence of Cre‐mediated excision, a single lox‐site remains at the junction between porcine and human sequence.Sanger sequencing confirmed also abundance of a correctly modified humanized exon 2. The orange box indicates the TGA‐nonsense codon causing the R31X mutation.

First, we assessed the translational potential of an USH pig model by examining the harmonin proteins and the underlying genetic *USH1C* structure across mammalian species. With the exception of a few small segments in the coding regions, harmonin shows an outstandingly high degree of conservation between the species (Fig 1A). Extended CCCTC‐binding protein (CTCF)/cohesin‐binding regions suggest that exons undergoing complex splicing are controlled by distantly located chromosomal segments in *cis* (Holwerda & de Laat, 2013; Ghirlando & Felsenfeld, 2016) rather than by local regulatory elements (Fig 1B). In contrast, a 1.5 kb segment comprising exon 2 and its surrounding intronic regions were predicted to be relevant in gene regulation (Fig 1C). Conservation is noticeable throughout the entire region, culminating in a 100% identity among all amino acid positions encoded by exon 2 in all examined species (Fig 1D). We therefore modified the porcine *USH1C* gene by a corresponding 1.5 kb element including a disruptive c.C91T/p.R31X nonsense mutation (Zwaenepoel *et al*, 2001) as well as 3 intronic single nucleotide polymorphisms (SNPs) identified on the disease allele of a human USH1C patient. A modification vector was produced by bacterial recombineering out of a bacterial artificial chromosome (BAC) covering the porcine *USH1C* locus and combined with CRISPR/Cas components to introduce the desired modification in pig primary cells (Fig EV1A–C and Appendix Fig S1A–D).

Following previous attempts to generate pig models (Kurome *et al*, 2015; Vochozkova *et al*, 2019), single‐cell clones (SSCs) were generated from primary cells, nucleofected with the modification components, and screened for the desired modification (Fig EV1E and Appendix Fig S1E and F). Somatic cell nuclear transfer (SCNT) was used for establishing founder animals from verified genetically modified SSCs (Fig EV1D). Female cell clones with both *USH1C* alleles modified were further lipofected with Cre‐mRNA in order to excise the selection cassette prior to their usage in SCNT experiments (Fig EV1F). Out of 12 embryo transfers, 4 litters were produced, delivering a total of 18 piglets. Genetic analysis confirmed excision of the *neo* cassette in 5 out of 15 founder animals, constituting a remnant single lox site in intron 1 (Fig EV1G and H), the humanized fragment (Fig EV1I) as well as the correct conjunction of the modification to the porcine sequence at the 3′‐end of the modification in the animals (Appendix Fig S1G).

Strikingly, all cloned founder animals showed a pronounced circling phenotype at birth (Movie EV1), correlating with a dysfunctional vestibular system (Ernest *et al*, 2000; Johnson *et al*, 2003). For this reason, animals were raised in a rescue deck from birth on (Egerer *et al*, 2018). After 48–72 h of interval feeding by hand, circling essentially ceased and USH1C piglets were able to feed themselves from the nurturing unit. We interpret this as central vestibular compensation and re‐weighting of multisensory input to the balance system.

In general, USH1C pigs developed and acted normally, but stress‐induced circling proved a consistent hallmark in the case animals found themselves challenged by new situations. At an age of 3–6 months, we occasionally observed a spontaneous nystagmus of the eyes (Movie EV2) in some animals. This is very likely caused by an asymmetrical tonic firing of vestibular afferents from the right and left labyrinth (Baloh *et al*, 2012; Dougherty *et al*, 2020) and has been anecdotally described in USH patients (Moller *et al*, 1989; Bonneau *et al*, 1993). 8‐month‐old USH1C pigs were fertile and 3 sows became pregnant after insemination with wild‐type (WT) sperm and gave birth to F1 litters, comprising heterozygous offspring (Appendix Fig S2A). F2 USH1C piglets were produced by mating F1 animals with each other or by inseminating of the cloned founder sows with sperm from one of their heterozygous offspring. In all USH1C offspring, a circling phenotype appeared as it had been observed in the F0 generation produced by SCNT. During breeding, it became obvious that some of the founder animals carried two distinct mutations of the *USH1C* alleles. One constituted the c.C91T/p.R31X mutation, while the other was a large genomic disruption, deleting exon 2 of the *USH1C* gene (Appendix Fig S2B). Genomic evaluation by endpoint PCR and qPCR and transcriptional analysis of heterozygous animals confirmed this hypothesis (Appendix Fig S2C and D and Appendix Fig S3). Details on animals used for experimental and breeding purposes are shown in Appendix Table S1.

### Motion analysis demonstrates vestibular dysfunction and visual deficits in USH1C pigs

**Figure 2:**
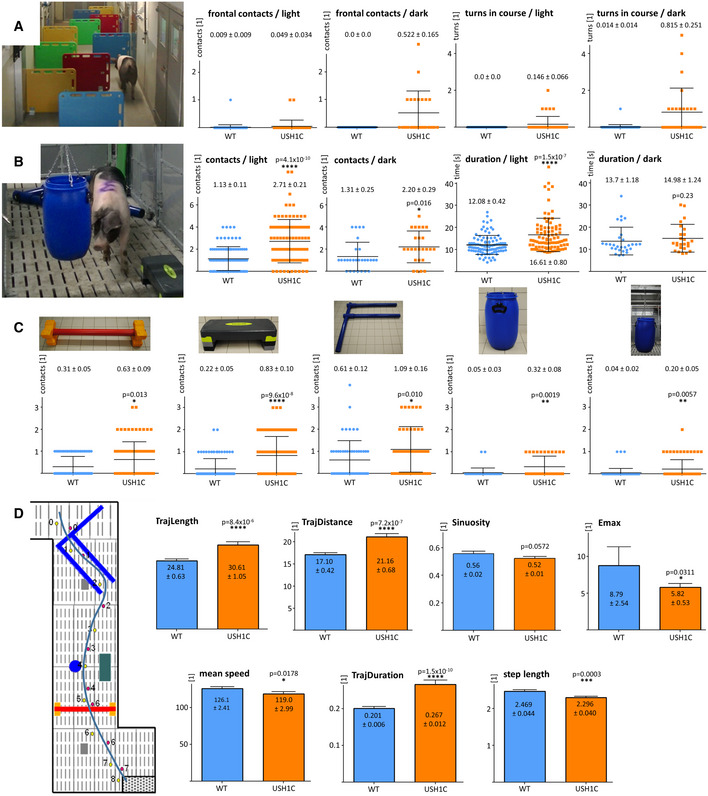
Impaired orientation of USH1C pigs in 3‐dimensional space A–CCohorts of animals were trained for specific parcour settings and then examined in repeated runs. Setting of hurdles was changed between the runs. Time and snout contacts were monitored. Analysis included all runs, except those in which the experimental pig did not meet the compliance criteria. Data points are plotted, lines indicate mean values (MV) ± SD, numbers give MV ± SE in addition. In a barrier course (A), different arrangements of shields (see Appendix Fig S4A) were used to challenge orientation. Only minor differences between WT (blue) and USH1C (orange) pigs were observed in the dark or under normal light conditions. Data points are summarized for two separate groups at 5–12 months of age (1 USH1C, 1 WT in 25 runs in the dark and 16 runs under light; 1 USH1C, 6 WT in 16 runs in the dark and 11 runs under light). (B) In a similar approach, different obstacles were placed instead of shields (see Appendix Fig S5) and revealed intensified sensing by snout contacts for USH1C pigs in the dark and under light. In the light, WT pigs were also faster. 5 USH1C and 5 age‐matched WT control pigs were examined in 22 runs under light conditions and 6 runs in the dark at 7–12 months of age. Statistical evaluation was performed by two‐tailed Mann–Whitney U‐test (*P*‐values indicated). For analysis of normalized data sets see Appendix Fig S6. (C) Detached analysis of the tests in the obstacle course (B) revealed that both, hurdles that primarily challenge the vestibular system (red cavaletti, blue F) as well as hurdles daring vision (sideboard, hanging or standing ton) provoked significantly higher alternative orientation by snout contacts for USH1C pigs. Statistical evaluation was performed by two‐tailed Mann–Whitney *U*‐test (*P*‐values indicated). *P < 0.05, **P < 0.01, ***P < 0.001, ****P < 0.0001.DPlotting individual runs from the obstacle course (B) to a planar matrix facilitated parameterizing the positions of the left (red) and right front leg (red) as well as the time when animals touch the ground (in seconds) and comparison of the trajectory to the positions of the hurdles. Data were then introduced in TrajR for evaluating standard motion parameters. Coordinates in the parcour were indicated in cm and Y‐axis of the respective parameters are given according the TrajR output. Error bars and numbers indicate mean values (MV) ± SE. *P < 0.05, **P < 0.01, ***P < 0.001, ****P < 0.0001.

To quantify changes in visually guided behavior, animals were challenged in distinct settings. In a barrier course in which animals had to bypass vertical shields to reach a food bowl (Barone *et al*, 2018), USH1C pigs did not show obvious differences to WT controls regarding duration or gait instability (Movies EV3, EV4 and Fig 2A and Appendix Fig S4). Interestingly, however, USH1C animals hesitated to enter the course in the dark (average 2.9 lux), turned around more often within the course, and had more frontal contacts with obstacles than WT control animals while these differences were less pronounced under light conditions. A more complex obstacle course was carried out on a cohort of 5 USH1C and 5 age‐matched WT controls. Over an age of 7–12 months, animals had to repeatedly walk through a parcour in which they had to cross or bypass distinctly shaped hurdles in varying constellations (Movies EV5, EV6 and Appendix Fig S5). In the dark (0.1–12 lux), USH1C pigs had significantly more contacts with hurdles, while their speed was similar to WT (Fig 2B and Appendix Fig S6). In the light (50–150 lux), USH1C pigs did not only touch hurdles more often, but were also significantly slower than WT controls. Plotting the data points for individual animals supported the interpretation of a general difference between USH1C pigs and WT control animals (Appendix Fig S7). Remarkably, USH1C pigs had difficulties to pass hurdles taking a pronounced vertical movement and therefore requiring balance (such as the red cavaletti) as well as obstacles challenging stereopsis and peripheral vision (such as the hanging or standing blue barrel and the step board) (Fig 2C). While the interpretation of an impaired vestibular system was further supported by the clearly different gait pattern in USH1C pigs (Movies EV5 and EV6), the interpretation of a reduced visual function in these animals was corroborated by a more detailed analysis of the movement in the obstacle course (Fig 2D). It became obvious that USH1C pig trajectories were longer (TrajLength and TrajDistance) compared to WT, with more frequently abrupt changes in direction (lower Emax). In addition, they took more time to complete the course (TrajDuration) while their step length was shorter.

### Clinical examinations confirm hearing loss and vision impairment in USH1C pigs

**Figure 3:**
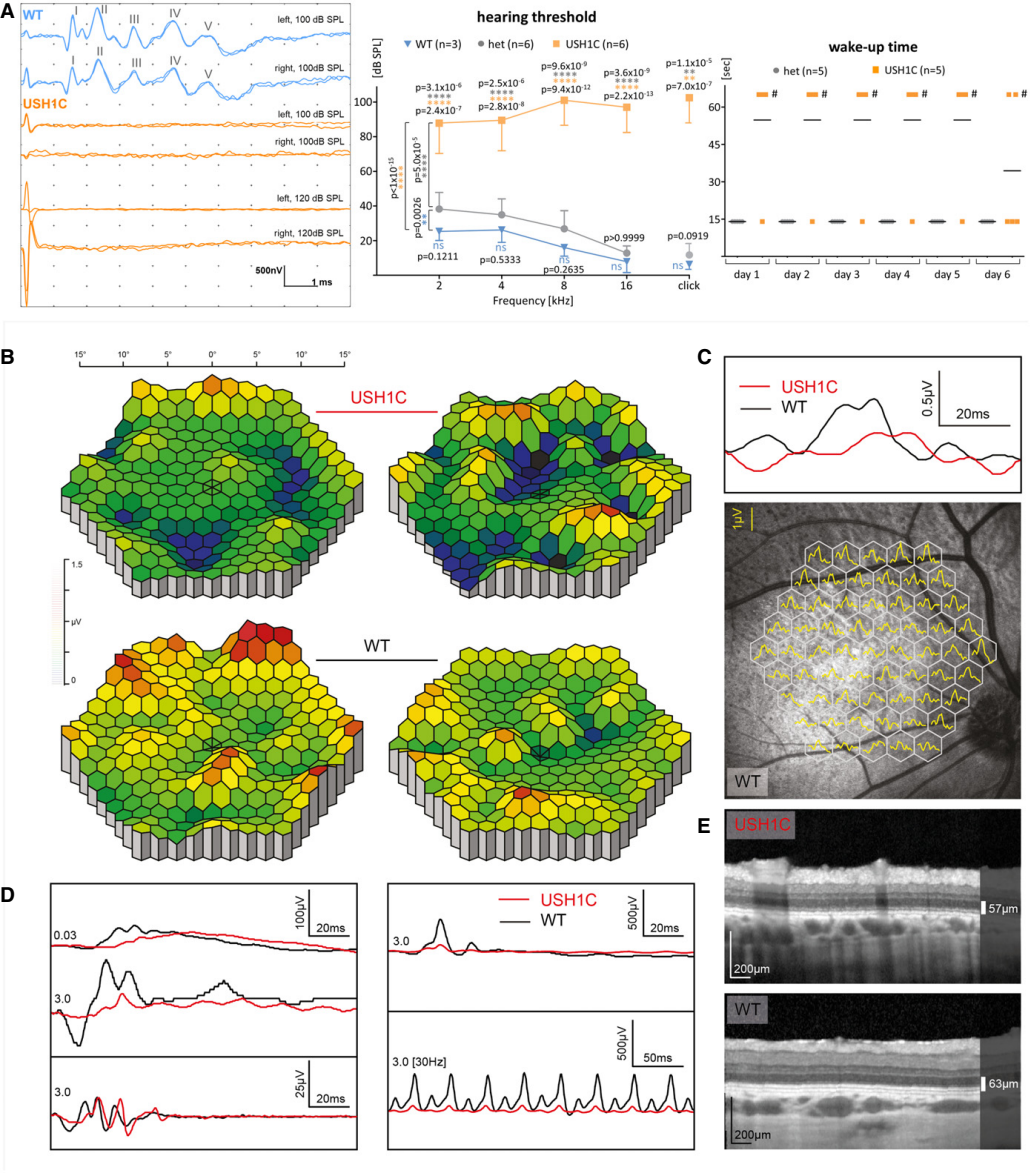
Hearing deficits and impaired vision in USH1C pigs ALeft Panel: Representative ABR to supramaximal click stimuli revealed stable response at the characteristic peaks I‐V in 3‐week old WT pigs at 100 dB SPL, but not in age‐matched USH1C pigs up to 120 dB SPL (left) (1 WT and 2 USH1C pigs, both ears, 2 traces each). Middle Panel: Hearing deficits measured by ABR were more pronounced at higher frequencies in 8‐week old USH1C pigs. Differences between WT and heterozygous USH1C^+/−^ (het) were not significant (*t*‐test) for the respective frequencies, but a 2‐sided ANOVA test indicated a < 0.01 significance (**) overall. *P*‐values for differences between USH1C pigs and WT or heterozygous pigs were < 0.0001 (****) at all tested frequencies and < 0.01 (**) for a click test. Data points represent mean values (MV) ± SD. Right Panel: Behavior tests supported impaired hearing in USH1C pigs. In their first days of life, reaction times of sleeping piglets upon calling by the mother sow were measured. Piglets waking up immediately entered the box within 15 s (Movie EV7). If not waking up within 60 s, piglets were stimulated by nudging (#). Mean values are indicated.B–EIn the eye, representative results were gained from 2 WT and 2 USH1C pigs at 12 months of age. For the limited number of animals, no statistical evaluation was performed. (B) A retinal dysfunction is evident in the multifocal electroretinogram (mfERG) of the visual streak in USH1C pigs compared to WT. Yellow/red colors indicate higher response amplitudes (1–1.5 µV), while blue/green colors indicate lower response amplitudes (0–1 µV) reflecting cone photoreceptor activity in the tested area. (C) Upper Panel: The average sum potential from the tested area highlights a delayed and lower peak amplitude in the visual streak in USH1C vs WT pigs. Traces indicate the average of all 61 hexagonal segments of single mfERG recordings. Lower Panel: Localization of the mfERG recordings. (D) Ganzfeld (gf) ERG recordings in USH1C pigs vs. WT demonstrate a significant reduction in the scotopic standard flash responses and responses after photopic standard flash stimuli. Left panel (top to bottom): Dark adapted single flash recordings following a dim light (0.03 cd*s/m^2^, rod only) or a brighter (3 cd*s/m^2^, rod and cone) stimulus. The oscillatory potentials shown at the bottom are extracted from the above standard flash recording. Right panel (top to bottom): Response after a light adapted single flash at 3 cd*s/m^2^ intensity and responses following a light adapted flicker (same intensity) at 30 Hz. (E) The retinal architecture as demonstrated by optical coherence tomography remained fairly intact, indicating a large therapeutic window.

**Figure EV2:**
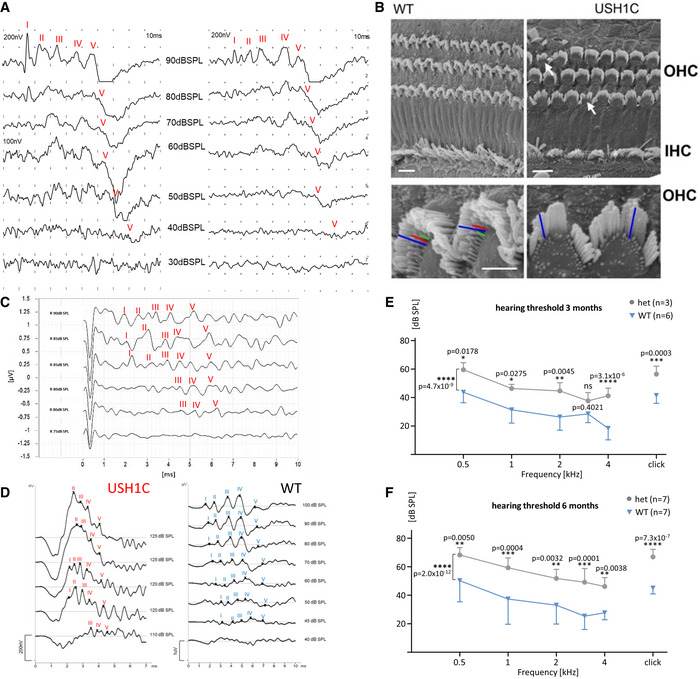
Impaired auditory system in USH1C pigs AABR measurements were conducted on two WT pigs to determine the threshold for sufficient detection of response peak V to click stimuli.BScanning electron microscopy of the cochlear sensory epithelium in 3‐week old WT and USH1C pigs reveals alterations in the hair bundle arrangements of outer (OHC) and inner cochlear hair cells (IHC) (arrows). USH1C OHC lack stereocilia rows in the hair cell bundles, indicated by colored lines at higher magnification. Scale bars, upper panel 10 µm; lower panel: 5 µm.C, D(C) and (D) show exemplary response curves for determining hearing thresholds (dB SPL) by click ABR in an 8‐week‐old USH1C animal (C) as well as 2‐year‐old USH1C and WT pigs (D).E, FFrequency‐specific ABR of animals at an age of 3 months (E) and 6 months (F) indicate an increased threshold for heterozygous USH1C^+/‐^ pigs, compared to WT littermate controls. Data points are presented as mean value (MV) ± SD. Statistical examination was carried out by *t*‐test for each frequency, a 2‐side ANOVA test was performed over all frequencies. *P < 0.05, **P < 0.01, ***P < 0.001, ****P < 0.0001.

Auditory brainstem response (ABR) documented severe hearing deficits in USH1C pigs as early as 3 weeks and up to 2 years of age, indicated by a lack of response even upon click stimulus at sound pressure levels of up to 120 dB sound pressure level (SPL) (Figs 3A and Fig EV2). The findings of sensorineural hearing loss (Cvejic *et al*, 2009) correlated with changes in the arrangement of hair cell cilial bundles and the lack of stereocilia in inner ear hair cells (Fig EV2B), as well as with the findings in *Ush1c* mouse models (Johnson *et al*, 2003; Lefevre *et al*, 2008) and the congenital deafness in human patients (Koenekoop *et al*, 1993). The hearing threshold of heterozygous USH1C^+/−^ pigs was slightly elevated compared to WT littermates at distinct ages (Figs 3A and EV2E and F). Hearing thresholds for both WT and heterozygous animals slightly declined with increasing test frequency, while thresholds in USH1C animals deteriorated further for higher frequencies. In line with their profound sensorineural hearing loss, USH1C piglets did not react on their mother´s call for nourishing (Movie EV7). Most of them needed nudging for waking up, while heterozygous littermates arrived at their mother within 15 s (Fig 3A). Consistently, some USH1C piglets were within the faster group, presumably awoken from being sideswiped by their littermates.

Clinical examination of the eyes in USH1C pigs at 1 year of age revealed changes comparable to early stages of USH in human patients. A reduction of cone photoreceptor function was evident in the central visual streak area analyzed by multifocal electro‐retinography (ERG, Fig 3B and C). Both rod and cone photoreceptor cell (PRC) responses were markedly attenuated in USH1C pigs compared to age‐matched wild‐type animals at 12 months of age (Fig 3D and Appendix Fig S8). USH1C animals showed > 70% reduction in the scotopic standard flash a‐wave amplitude ([mean value ± SD] 21 ± 18 µV in USH1C vs. 79 ± 2 µV in WT animals) and a > 50% reduction in scotopic b‐wave amplitude (75 ± 32 µV in USH1C vs. 154 ± 39 µV in WT animals). Responses after photopic standard flash stimuli showed a 50% reduction of a‐wave amplitude ([mean ± SD] 10 ± 0.1 µV in USH1C vs. 20 ± 5 µV in WT animals) and a 60% reduction in the respective b‐wave amplitude (86 ± 49 µV in USH1C vs. 215 ± 50 µV in WT animals). Confocal scanning laser ophthalmoscopy (cSLO) of the retina and choroid layer showed no differences between WT and USH1C pigs (Appendix Fig S9). Interestingly, spectral domain optical coherence tomography (OCT) (Fig 3E) revealed only minor differences in retinal architecture between WT and USH1C pigs with good preservation of outer retinal layers containing the PRCs, suggesting a large therapeutic window.

### Lack of harmonin in sub‐cellular structures disrupts photosensitive disc architecture

**Figure EV3:**
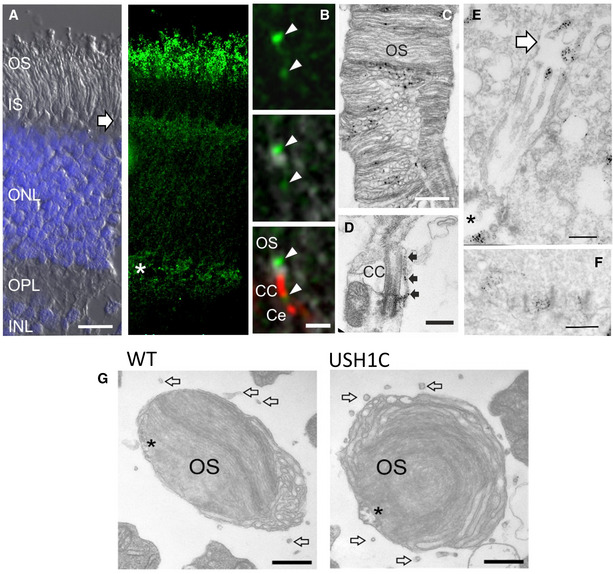
Localization of harmonin in the WT retina Representative staining of longitudinal sections from porcine WT retina (2 pigs, 1 retina each, 4 TR).
AHarmonin (green) is present in the layer of PRC outer segment (OS), the outer limiting membrane (arrow) and in the outer plexiform layer (OPL, asterisk), where PRC synapses are located. Left image: DIC image super‐exposed with DAPI for nuclei in outer nuclear layer (ONL) and inner nuclear layer (INL). Right image: Immunohistological staining with anti‐harmonin antibody. IS, PRC inner segment. Scale bar 10 µm.BImmunohistological co‐localization of harmonin (green) and the connecting cilia (CC)/centriole (Ce) marker centrin (red) at the OS base (arrowheads, scale bar 1 µm).C, D(C) Immunoelectron microscopy detects harmonin at PRC OS discs (scale bar 500 nm) as well as (D) at the base of connecting cilia (CC, scale bar 500 nm) and in calyceal processes (arrows).E, F(E) In Müller glia cells, harmonin is localized at microvilli tips (arrow, scale bar 500 nm), at the cell adhesion region (asterisk) as well as (F) in cone synaptic pedicles (scale bar 500 nm).GHorizontal cross sections through cone OS reveal persistence of calyceal processes in USH1C pig (arrows, scale bar 400 nm). Asterisks indicate axoneme projections into the OS.

**Figure 4:**
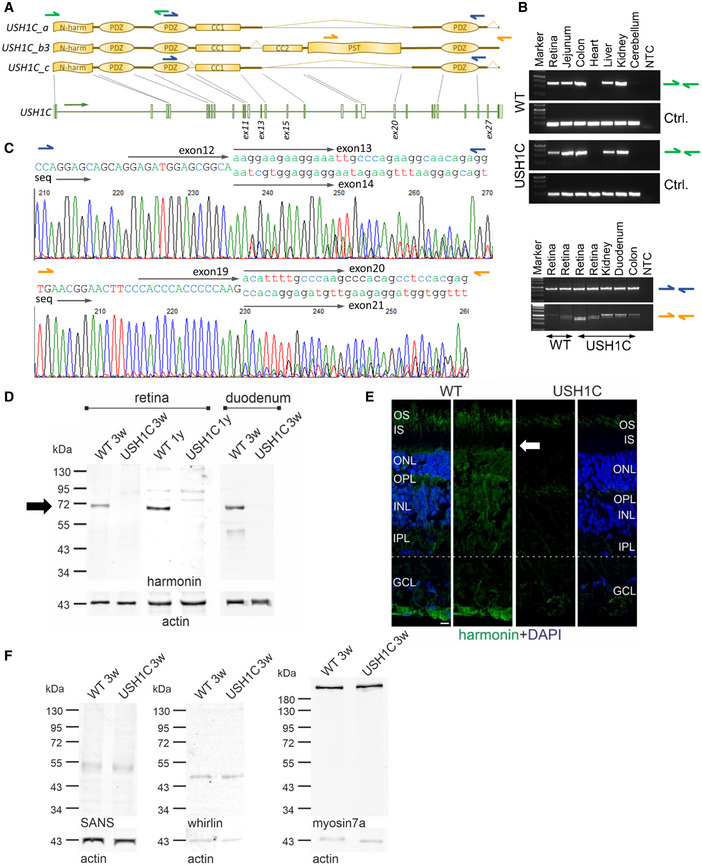
Lack of harmonin does not alter expression of other USH proteins RT–PCR was used to detect any *USH1C* transcripts (green arrows) and for discriminating *USH1C_a* and *_c* splice variants (blue arrows) from *USH1C_b3* (orange arrows).
*USH1C* transcripts were consistently detected in all tested organs of WT and USH1C pigs, except in the heart and cerebellum. RT–PCR for *GAPDH* was used as control (Ctrl.).
*USH1C_a* or *_c* splice variants were detected in the retina, kidney and intestine (blue arrows). Electropherogram from the duodenum, confirm alternative splicing of exon 13, but not exon 11. *USH1C_b* variants were also detected in all examined organs, with presumably two splice variants (orange arrows). Electropherogram from the colon, indicates alternative splicing of exon 20 as presumable reason for the double band. Exon 27 was not detected in any amplicon of *USH1C_b* RT–PCR (Appendix Fig S10D and E). Sequencing was done with a forward primer.Representative Western blot analysis proved lack of harmonin protein expression in retina and duodenum of USH1C pigs at the age of 3 weeks (3w) (1 piglet, 1 retina, 1 duodenum) and 1 year (1y) (2 pigs, 1 retina each, 3 TRs). Arrow indicates the expected size of harmonin isoform a (72 kDa).Representative immunofluorescence staining of harmonin (green) of longitudinal cryosection through WT and USH1C pig retinas (2 pigs, 1 y, 1 retina each, 3 TRs, scale bar 10 µm) demonstrated the absence of harmonin staining in the outer segment (OS) of photoreceptor cells and in the other layers below the outer limiting membrane (OLM, arrow) of the USH1C pig retina.Representative Western blot analysis of retinal protein extracts, indicating that the abundance of the USH proteins SANS (USH1G), whirlin (USH2D) or myosin7a (USH1B) is unaffected (1 piglet, 3w, 1 retina each, 2 TRs).

**Figure 5:**
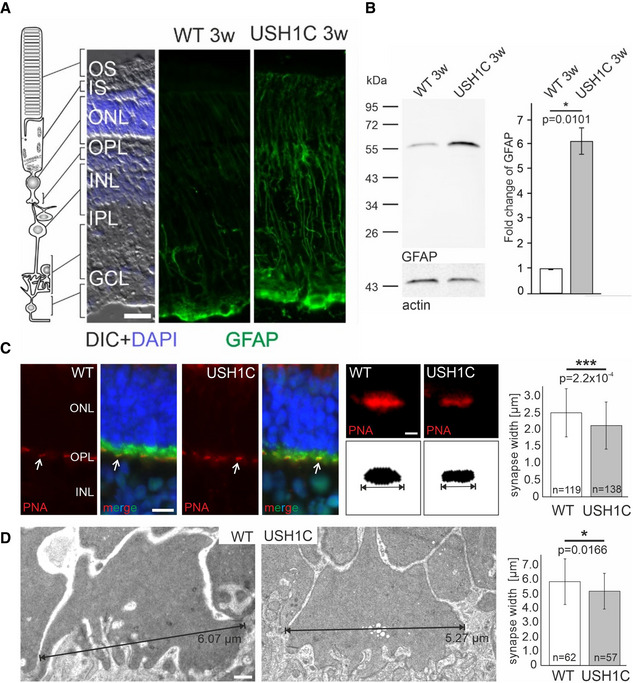
USH1C^R31X^ induce upregulation of GFAP and a synaptic phenotype in USH1C pig photoreceptor cells A, BUpregulation of GFAP in the retina of USH1C pigs at an age of 3 weeks (3 w). (A) Representative immunofluorescence staining of GFAP in Müller glia cells which extend throughout almost the entire retina from the OLM (arrow) to the ganglion cell layer (GCL) of the retina. The consistent increase of GFAP expression in the Müller glia of USH1C pigs indicates Müller cell activation and gliosis. IS, inner segment; ONL, outer nuclear layer; OPL, outer plexiform layer; INL, inner nuclear layer; IPL, inner plexiform layer; scale bar: 20 µm. (B) Left panel: Western blot analysis of GFAP protein expression in 3 w old USH1C piglets and age‐matched controls. Anti‐actin Western blot was used as loading control. Right panel: Quantification of Western blot bands in 4 gels by the LI‐COR Odyssey system revealed a strong increase GFAP expression in 3w USH1C piglets when compared to age‐matched WT controls (2 piglets, 3w, 1 retina each, 2 TRs, error bars represent SD of the mean, two‐tailed Student’s *t*‐tests, *P < 0.05).C, DReduced synaptic width in USH1C pigs at an age of 1 year (1 y). (C) Fluorescent microscopic analysis of the cone synapse phenotype. Left panel: Representative images of longitudinal sections through WT and USH1C pig retinae stained for the pre‐synaptic marker PSD‐95 (green) and by fluorescent peanut agglutinin (PNA, red) for cone synaptic pedicles (white arrows) and counter‐stained by DAPI for nuclear DNA (blue). Scale bar 10 µm. Middle panel: Higher magnification of a PNA‐stained cone synaptic pedicle. Synapse width was determined as the maximum extension of consistent PNA signals. Scale bar 1µm. Right panel: Measuring cone synaptic pedicle width in WT and USH1C pigs by applying a Fiji script to PNA‐stained sections indicated reduced synaptic width (2 pigs, 1 y, 1 retina each, number of examined synapses indicated, error bars represent SD, two‐tailed Student’s *t*‐tests, ***P < 0.001). (D) Determining cone synaptic pedicles width by TEM. Left panel: Representative images of retinal cross sections. Scale bar 500nm. Right panel: Quantification of synaptic width confirmed the significantly reduced width of cone synaptic pedicles in USH1C pigs. (1 pig, 1 y, 1 retina each, number of examined synapses indicated, error bars represent SD, two‐tailed Student’s *t*‐tests, *P < 0.05).

Previous studies of *USH1C*/harmonin expression, particularly in the mouse retina, were inconsistent but indicated localization of harmonin in PRC, MGC, and secondary retinal neurons. (Reiners *et al*, 2003; Reiners & Wolfrum, 2006; Williams *et al*, 2009b; Sahly *et al*, 2012). In WT pigs, we detected harmonin in the PRC layer, the outer limiting membrane (OLM) and outer plexiform layer (OPL) (Appendix Fig EV3). More specifically, the protein was found in the PRC outer segment (OS), the OS base, in calyceal processes (CP) and synaptic pedicles. Harmonin abundance in MGC microvilli tips and the cell adhesion region of the OLM is in line with expression profiles and subcellular localization of harmonin in the human retina (preprint: Nagel‐Wolfrum *et al*, 2021). In both, USH1C and WT pigs, *USH1C* transcripts were detected in multiple but not all organs (Fig 4A and B). Sanger sequencing confirmed correct splicing of the humanized exon 2 in USH1C pigs (Appendix Fig S10A and B). While in some tissues (colon, duodenum), RT–PCR indicated considerable proportions of transcripts lacking exon 13 (Fig 4C), splicing out of exons 13 or 14 appeared only at very low levels in the retina (Appendix Fig S10C). *USH1C_b* splice variants were found at low level in general, but clearly indicated a lack of exon 27 in all transcripts and alternative splicing of exon 20 (Appendix Fig S10D and E). We did not detect transcripts without exon 11, a core characteristic of *USH1C_c*, indicating that *USH1C_a1* is the dominant transcript of *USH1C* in the pig retina. At the protein level, harmonin was consistently lacking in Western blot analysis (Fig 4D) and in tissue sections (Fig 4E), confirming the sufficient suppression of harmonin translation by the premature R31X nonsense mutation. No changes were observed in protein levels of SANS (encoded by *USH1G*), whirlin (encoded by *WHRN* (*USH2D*)), and myosin VIIa (encoded by *MYO7A* (*USH1B*)) in the retina (Fig 4F). In contrast, glial fibrillary acidic protein (GFAP) was considerably upregulated in the USH1C retina already at an age of 3 weeks (Fig 5A and B), suggesting gliosis, a phenomenon often seen in inherited retinal diseases (Hippert *et al*, 2015) or injury‐mediated degradation processes (Bringmann *et al*, 2009).

**Figure 6:**
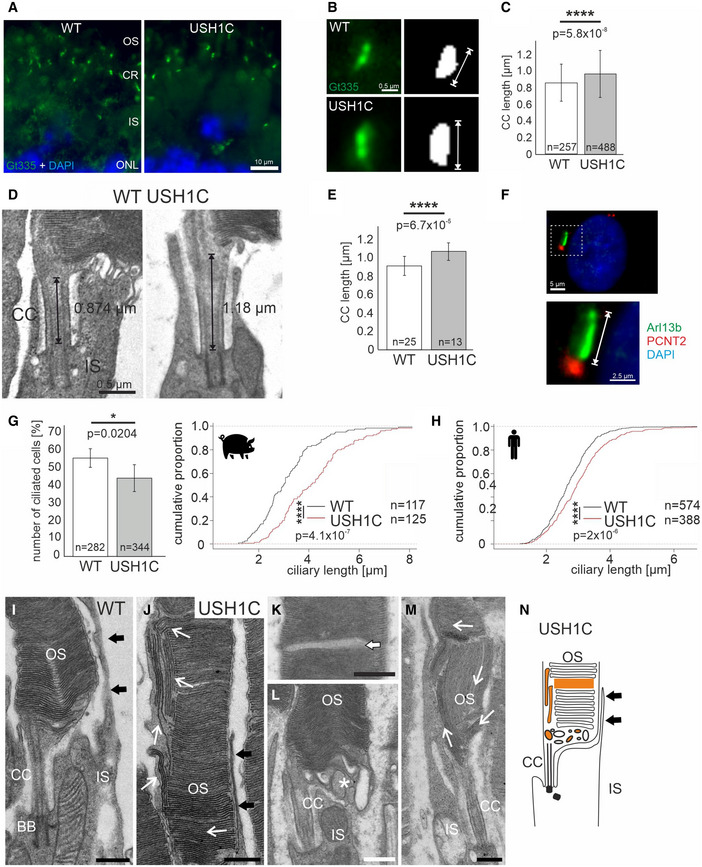
Changes in ciliary structures and outer segment architecture of USH1C photoreceptor cells A–CCiliary length measured by fluorescence microscopic analysis at 1 year of age (1y). (A) Representative immunostaining of the ciliary marker Gt335 (green) in longitudinal cryosections of PRC ciliary regions in WT and USH1C retinas. Counterstaining by DAPI, scale bar 10 µm. (B) Gt335 staining at higher magnification and principle of quantifying ciliary length by Gt335 staining. Scale bar 500nm. (C) Length of connecting cilia (CC) appear elongated in USH1C when applying a Fiji script to immunostained histological sections (2 pigs, 1y, 1 retina each, number of examined connecting cilia is indicated, error bars represent SD, two‐tailed Student’s *t*‐tests, ****P < 0.0001).D, EMeasuring CC length in TEM sections. (D) Representative images of longitudinal cross sections and principle of determining CC length. Scale bar 500 nm. (E) Elongation of CC length is confirmed in TEM sections of USH1C PRC. (1 pig, 1y, 1 retina each, number of examined connecting cilia indicated, error bars represent SD, two‐tailed Student’s *t*‐tests, ****P < 0.0001).F–HLength of primary cilia in dermal fibroblasts. (F) Staining of ciliary structures in fibroblasts stained with ARL13B (ciliary shaft, green) and PCNT2 (ciliary base, red), DAPI (nucleus, blue). Scale bar upper panel 5 µm, lower panel 2.5 µm. (G) Left panel: The number of ciliated cells is decreased in porcine fibroblasts of USH1C pigs, compared to WT controls (6 TR, number of examined cells indicated, error bars represent SD, two‐tailed Student’s *t*‐tests, *P < 0.05). Right panel: The length of primary cilia in porcine fibroblasts is increased in USH1C (6 TR, number of cells examined indicated, Kolmogorov–Smirnov (KS) test, ****P < 0.0001). (H) Similarly, ciliary length of fibroblasts from a human USH1C^R31X/R80fs^ patient is elongated, compared to a healthy control (7 TR, number of cells examined indicated, KS‐test, ****P < 0.0001).I–MOuter segment (OS) PRC architecture by TEM of longitudinal retinal sections. (I) At 3w, WT show normal sub‐cellular organization, including parallel stacking of photosensitive discs and calyceal processes (black arrows, representative image from 2 pigs, 2 retinas, scale bar 600 nm). (J) In 3w USH1C rods, membrane discs appear also in vertical orientation (white arrows, representative image from 2 pigs, 2 retinas, scale bar 750 nm). At 1 y, interstitial gaps appeared in disc stacks of the OS in USH1C pigs (K, arrow, scale bar 550 nm) and vesicles are found at the OS base (L, asterisk, scale bar 500 nm). (M) The disc architecture in 1 y animals was substantially distorted (arrows, representative image from 2 pigs, 2 retinas, scale bar 850 nm).NLocalization of cellular anomalies in an USH1C rod highlighted in orange.

While the general structure of the retina appeared grossly intact in USH1C pigs (Figs 4E and EV3G, and Appendix Fig S9), subcellular analysis revealed specific disruptions in the architecture of compartments containing harmonin. In line with its proposed scaffold function in ribbon synapses (Gregory *et al*, 2013), the loss of harmonin results in significantly reduced extension of the cone synaptic pedicles. This was confirmed by both the quantitative determination of cone‐specific peanut agglutinin (PNA) stretches at synapses (Fig 5C) and the direct measurement of synapse width in transmission‐electron microscopy (TEM) (Fig 5D). We also found significant increases in length of the connecting cilium (CC) in rod PRCs, as determined by quantitative measurements of the ciliary marker GT335 stretches (Fig 6A–C) as well as by direct examination of TEM ultrathin sections (Fig 6D and E). Aiming at identifying a consistent relevance of harmonin for cilia formation, we examined primary cilia in dermal fibroblast of USH1C and WT in pigs and humans (Fig 6F). While the number of ciliated cells in USH1C pigs was reduced compared to WT (Fig 6G), the length of the primary cilia was consistently elongated in fibroblasts isolated from USH1C pigs and a USH1C^R31X/R80fs^ patient after induction of ciliogenesis (Fig 6G and H). Most striking, however, was the deprivation of rod OS architecture in PRC. While this is normally characterized by a strictly horizontal stacking of parallel photosensitive membrane discs (Fig 6I) (Sjostrand, 1953), we detected vertically orientated membrane discs (Fig 6J) in animals as early as 3 weeks of age. This aberrant structuring became even more prominent at 12 months when the stacking of discs was further infringed by interstitial gaps and vesicle‐like structures at the OS base (Fig 6K–M). In contrast to rods, the OS architecture in cones remained normal throughout the first year of life (Fig EV3G).

### USH1C pigs facilitate testing of therapeutic approaches *in vivo* and *ex vivo*

Besides its usage in studying disease progression and its underlying mechanisms, the USH1C pig will also support the development of novel treatment approaches. The defined genetic cause of the model, the disruption of exon 2, affects all relevant USH1C splice forms and, therefore, facilitates the testing of a broad spectrum of treatment options. We addressed fundamental questions by examining the tropism of distinct serotypes of adeno‐associated viruses (AAV) for transducing cells of the retina *in vivo*, by testing the gene therapy and gene repair approaches *in vitro* and by exploring the potential to restore eye function *in vivo* after sub‐retinal injection of AAV expressing USH1C mRNA.

**Figure 7:**
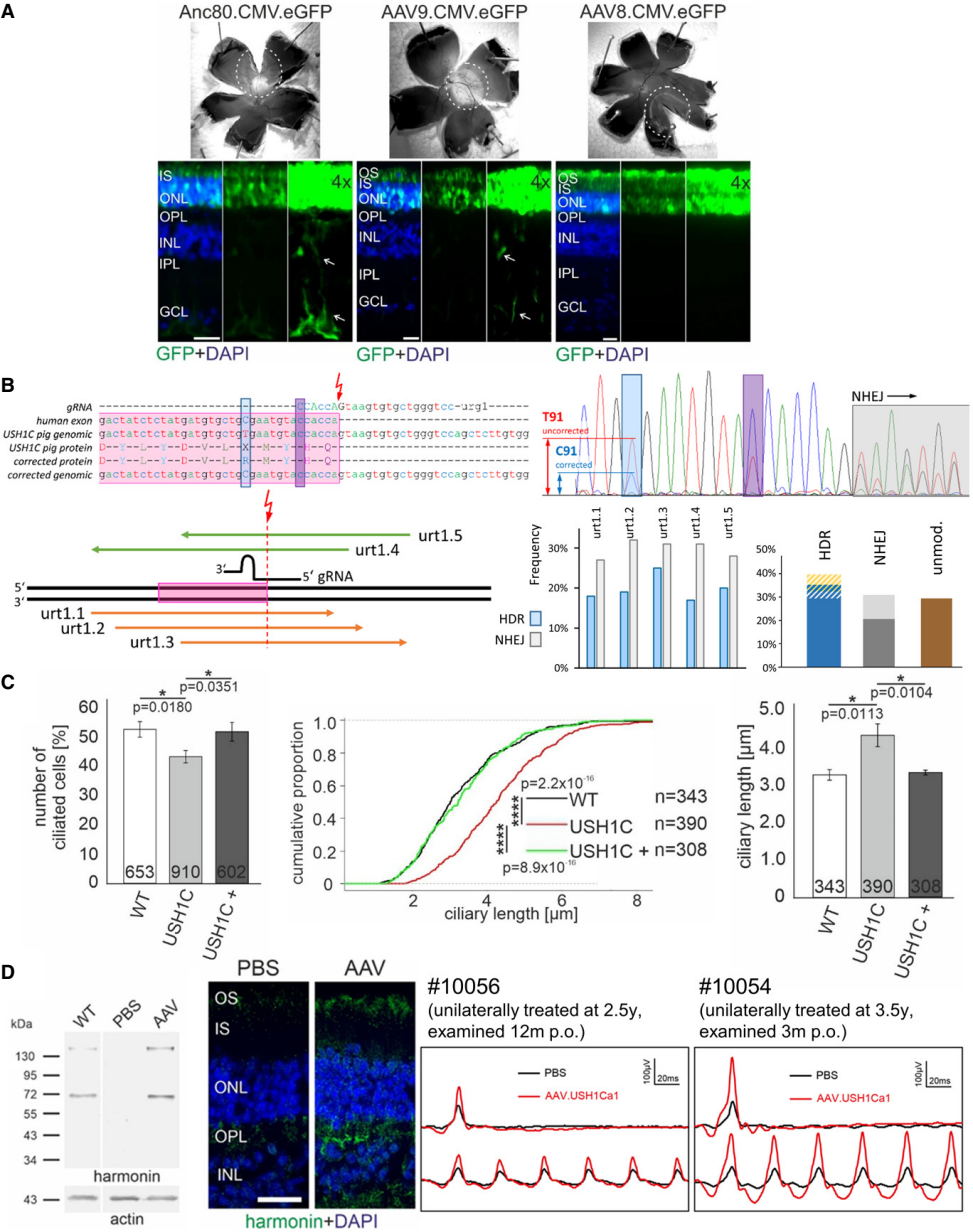
Therapeutic approaches AAV transduction of retinal layers. AAV8, AAV9, and Anc80 encoding eGFP under the ubiquitous CMV promoter were injected subretinally into the eyes of mature WT pigs *in vivo*. Upper Panel: Epifluorescence of dissected eyes demonstrates eGFP fluorescence in the bleb region of whole mount preparations of all three AAV samples. Dashed circles indicate bleb size. Lower panel: Epifluorescence images of longitudinal retina sections revealed eGFP expression in the PRC layer (OS, IS and ONL) for all AAVs. Increased exposure (4×) documented transduction of MGC (arrows) by AAV9 and Anc80 but not by AAV8 (representative images from 1 pig, 1 retina each, 2 TR, scale bars 25 µm).CRISPR/Cas‐mediated gene repair was tested in primary cells of USH1C pigs (see also Fig EV4). Upper left: The cutting site (red arrow) of the most favorable gRNA was directly at the transition from exon 2 (pink) to the downstream intron. Positions of the correcting mutation (blue) and blocking mutation (magenta) are indicated. Lower left: Distinct ssODN binding to the sense (green) or anti‐sense strand (orange) were compared. Upper right: Sanger electropherograms confirmed the correct introduction of the correcting (blue) and blocking (magenta) mutations and indicated by standing NHEJ mutations (grey, overlapping nucleotide peaks) at the gRNA cutting site. Lower right: Frequencies of HDR (blue) and NHEJ (grey) in mixed cell populations were similar for the tested ssODN templates (left). 68 single cell clones treated with urt1.3, indicated that HDR appeared either exclusively (dark blue) or was accompanied by intronic (blue/white shaded) or exonic (blue/gold) mutations (right). In some clones, HDR transformed only the blocking mutation site which is more closely located to the cutting site (white/gold). NHEJ events appeared as insertions (dark grey) or deletions (light grey). The frequency of unmodified clones is depicted in brown.Effects of harmonin_a1 expression were investigated in dermal fibroblasts. Left panel: Transfection of USH1C primary cells with an expression plasmid (USH1C^+^) increased the number of ciliated cells toward WT level (3 TR, number of examined cells is indicated, error bars indicate SD, two‐tailed Student’s *t*‐test, *P < 0.05). Middle and right panel: Length of cilia in USH1C primary cells was shifted toward WT level (3 TR, number of examined cells is indicated, error bars indicate SD, Kolmogorov–Smirnov test, *P < 0.05, ****P < 0.0001).Efficacy of AAV gene therapy was examined *in vivo* by sub‐retinal injection of Anc80 virus capsid expressing harmonin_a1 under a ubiquitous promoter into a USH1C pig (see also Fig EV5). Left panel: Sub‐retinal injection reconstituted harmonin expression in the AAV‐treated eye but not in the sham‐treated eye (3 TR). Protein extract from a WT retina served as positive control, actin staining as loading control. The 72 kD band correlates with harmonin_a isoforms while higher MW indicates putative dimer formation. Middle panel: Immunofluorescence reveals absence of harmonin in the retina of the PBS‐injected control eye and harmonin abundance (green) in the retina of the AAV‐treated eye. (Representative image from a single animal. Blue: DAPI, scale bar 25 µm). Right panel: Photopic gfERG reveal increased a‐ and b‐wave response to single flash and flicker stimulation in the AAV‐treated (red), compared to the sham‐treated (black) eyes of two USH1C pigs.

For testing the distribution of AAV vectors, defined amounts (200 µl, 2 × 10^10^ vg/eye) of established AAV8, AAV9, or a modified Anc80 vectors, each of them expressing eGFP under control of a CMV promoter (Vandenberghe *et al*, 2013; Carvalho *et al*, 2018), were injected into the sub‐retinal space of the eye in WT pigs followed by examination of eGFP expression after 5 weeks. While AAV8 transduced only PRCs efficiently, AAV9 and Anc80 transduced both, PRC and MGC (Fig 7A, lower panel). Anc80 was much less effective in transducing cells of the retinal pigment epithelium (RPE) than AAV9 and, in particular, than AAV 8 (Appendix Fig S11), indicating that the target specificity in the eye can be controlled by the appropriate choice of the AAV capsid. No relevant other off‐target expression of eGFP was noted for the tested AAVs.

**Figure EV4:**
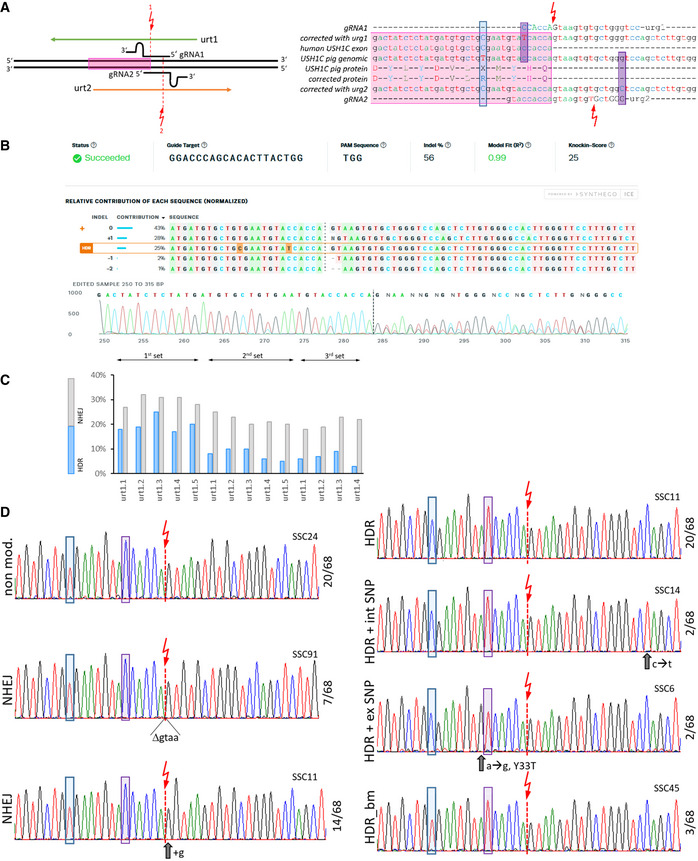
CRISPR/Cas‐mediated gene repair In an initial approach the 2 oppositely oriented gRNA1 and gRNA2 and repair oligonucleotides urt1 and urt2 were tested for their efficacy to introduce NHEJ‐mediated indel formation. Exon 2 is marked by a pink box. The cutting sites of the Cas9 are shown as red arrows and the distinct positions at which the respective oligo‐nucleotides should introduce blocking mutations are indicated by magenta boxes. The position of T91 and its corrected variant C91 is indicated by a blue box.Sanger sequencing electropherograms were used to estimate efficacy of HDR after co‐transfection of plasmids expressing Cas9 and a gRNA and commercially synthesized ssODN repair templates into primary cells from USH1C pigs. PCR products from mixed cell clones were analyzed for NHEJ and HDR by the ICE CRISPR Analysis Tool.Optimization was performed with gRNA urt1 and five distinct repair oligo‐nucleotides in three independent experiments. The rate of HDR and NHEJ was determined as in (B).Single cells clones were generated from the pool nucleofected with gRNA1 and urt1.3 and analyzed by Sanger sequencing of PCR products. A diverse pattern of distinct modifications was observed in 68 examined single cell clones (SSCs). Representative electropherograms from designated SSCs are shown with the frequency of the respective pattern indicated at the right side. The cutting site of Cas9 as well as the correcting and blocking mutation are indicated as in (A). “non mod.” indicates SSCs without changes at the target site. NHEJ appeared either as deletions or insertions. HDR events were mostly restricted to the correct transformation of the correcting and blocking mutations. Some SSCs, however, showed accompanying mutations in the intronic (int SNP) or exonic (ex SNP) regions, with the latter potentially causing amino acid exchanges. Occasionally as well, HDR occurred only at the blocking mutation site, which is located closer to the cutting site than the correcting mutation site.

The capability for correcting the causative *USH1C^R31X^* mutation in the humanized exon 2 by CRISPR/Cas‐mediated gene repair was evaluated by applying distinct constellations of human‐specific gRNA and repair oligo‐nucleotides into pig primary kidney cells (PKCs), isolated from USH1C founder animals (Figs 7B and EV4). The efficacy of transforming the nonsense codon TGA into an Arginin‐encoding CGA by homology‐directed repair (HDR) reached 40%, determined in DNA preparations from mixed cell clones. Upon clonal selection, the analysis of 68 single cell clones (SSCs) confirmed that 40% of the clones underwent gene correction, while non‐homologous end‐joining (NHEJ) was seen in 31% of the SSCs. Occasionally, HDR was accompanied by additional mutations, either small insertions/deletions or single nucleotide exchanges. For the specific positioning of the gRNA, a considerable proportion of mutations that appear in intron 2 would not affect the correct transcription and translation of *USH1C*.

For exploring the potential of harmonin restoration by gene therapy, we transfected a vector encoding the human harmonin_a1 splice variant into primary skin fibroblasts derived from *USH1C* pigs. By using the number of ciliated cells as well as the length of the primary cilium as therapeutic readout (Fig 6G and H), we confirmed that treated fibroblasts differed significantly from untreated USH1C cells and reached the levels determined in WT fibroblasts for both parameters (Fig 7C).

**Figure EV5:**
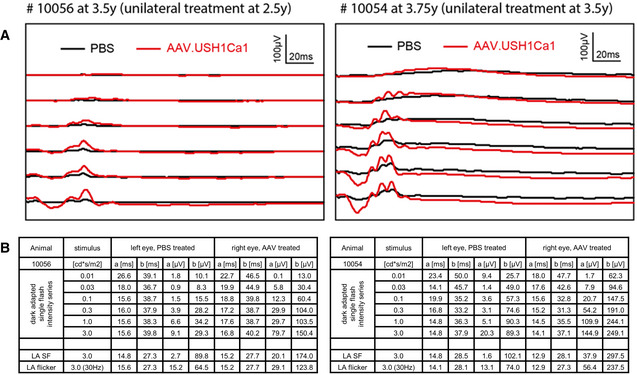
Efficacy of AAV gene therapy in USH1C pigs 2 USH1C pigs underwent sub‐retinal injection of an Anc80‐capsid expressing harmonin_a1 under control of a CMV promoter in the right eye and of PBS in the left eye. Pig 10056 was injected at an age of 2.5 years (2.5 y) and terminated after 12 months with ERG evaluation and tissue sampling for molecular analysis. Animal 10054 was injected at an age 3.5 y with the same setting and monitored intermittently after 3 months.
Response to single flashes after dark adaptation in the AAV‐treated eye (red) and in the sham‐treated eye (black).Quantification of response times and amplitudes of a‐ and b‐waves after dark in two treated USH1C pigs. LA SF, light adapted single flash. LA flicker, light adapted flicker.

The potential to compensate for the lack of harmonin in USH1C pigs was explored by applying a volume of 200 µl, 1 × 10^11^ vg Anc80.CMV.harmonin_a1 vector into the sub‐retinal space of one eye while the other eye was sham‐treated with PBS. In one 3.5‐year‐old USH1C pig, we detected harmonin in histological sections and in Western blot after 12 months in the treated eye (Fig 7D). Furthermore, amplitudes of the a‐ and b‐waves were significantly increased in the scotopic and photopic ERG, compared to the sham‐treated eye, similarly to another animal that was treated accordingly at an age of 3.5 years and examined after 3 months (Figs 7D and EV5).

## Discussion

Combining hearing deficits, vestibular dysfunction, and vision loss, the USH1C pig is the first animal model to reflect the full syndromic phenotype in human USH1 patients. The early onset of symptoms makes it suitable for studying the basic mechanisms of the disease as well as for evaluating strategies of therapeutic intervention. The persistence of a largely intact retinal architecture throughout the first year of life provides a wide therapeutic window and the potential to revert the pathogenesis by targeting dysfunctional, but still viable photoreceptor cells. The specific design of the genetic modification allows the examination of a broad diversity of therapies, including classical additive gene therapy, human‐specific gene repair, and translational read‐through or cell replacements. The establishment of breeding populations (Appendix Fig S2), the provision of distinct therapeutic options *in vitro* and the success of AAV‐based delivery of harmonin in exploratory experiments *in vivo* (Figs 7, EV4, and EV5) collectively demonstrate the potential of the model for evaluation of therapeutic interventions. Consequently, USH1C pigs can now be used for systematic pre‐clinical examination *in vivo*, similar to recent ventures in genetically engineered pig models (Langin *et al*, 2018; Regensburger *et al*, 2019; Moretti *et al*, 2020). In such studies, housing USH1C pigs under standardized conditions will minimize environmental confounding, whereas the pronounced genetic diversity of conventionally produced pigs needs consideration. While presenting a statistical challenge for pre‐clinical proof‐of‐principle studies, this heterogeneity is, of course, much more likely to reflect the scenario in clinical trials and might thus lead to a more robust predictive power of the model in the translation of new therapeutic strategies (Ebermann *et al*, 2010).

The manifestation of an ocular phenotype in USH1C pigs is striking, taking into account the multiple efforts that have been made in creating such models in other species with so limited success (Williams, 2008). Our results reinforce some of the multiple sentiments that have been postulated as reasons for the lack of a vision phenotype in rodents. The onset of retinal dysfunction in pigs within the first year of life argues against the notion that the limited life span of mice prevents the developing of a similar phenotype in the mouse eye. While a potentially distinct function of the USH proteins in the retinae of different species cannot be readily excluded, the degree of sequence conservation of harmonin and its immediate interaction partners is remarkable (Fig 1 and Appendix Fig S12), insinuating a conserved and consistent nature of the USH1 interactome across species.

Crucially, porcine harmonin appears to be localized to CP (Fig EV3D) which is thought to stabilize the OS structure in the PRC of different species, although not in rodents (El‐Amraoui & Petit, 2014). A recent publication demonstrated the absence of CP in cone PRCs and a pronounced outgrowth of disc membranes at the base of rod OS upon partial knock‐down of the *USH1F*‐encoded protein pcdh15 in the clawed toad (Schietroma *et al*, 2017). While the complete lack of harmonin in pig did neither show outgrowth of disc membranes nor affect the formation of CP to a similar extent, it explicitly disturbed the photosensitive disc architecture in rods (Fig 6I–N). Considering the actin‐binding properties of harmonin, the changes in disc architecture and CC dimensions (Fig 6A–E) link the retinal pathology in USH1C pigs to the proposed role of the actin cytoskeleton as driving force in disc neogenesis and in the structural maintenance of the PRC OS (Corral‐Serrano *et al*, 2020; Spencer *et al*, 2020). Specifically, ciliogenesis has been shown altered upon dysregulation of the actin cytoskeleton at a molecular level (Kim *et al*, 2015), an effect that has been also associated with neurodegenerative diseases (Karam *et al*, 2015). The consistent elongation of CC in PRC and primary cilia in dermal fibroblasts (Figs 6 and 7C) further confirms the tight interaction of the ciliary actin system with axonemal microtubules in PRCs (Wolfrum & Schmitt, 2000; Corral‐Serrano *et al*, 2020) and the general role of the actin system in the organization of ciliary axonemes (Kiesel *et al*, 2020). Although precise mechanistic insights into the role of harmonin in cilia function require further studies, our data provide clear evidence that USH is a true retinal ciliopathy in its fundamental aspects (Bujakowska *et al*, 2017).

Remarkably, the significantly disturbed photosensitive disc architecture in rod OSs and the ciliary aberrations (Fig 6) are associated with a significant (c. 70%) reduction in rod‐derived ERG responses (Fig 3D). The apparent decline in cone‐derived ERG (c. 50%) and the reduced width of cone synaptic pedicles (Fig 5C and D) indicate the early onset of cone‐specific alterations as well. Both, structural and clinical observations thus support the notion of a rod‐cone dystrophy phenotype in USH1C pigs, reflecting the situation in human patients quite well (Koenekoop *et al*, 1993; Khateb *et al*, 2020). The early onset of PRC malfunction was in line with the up‐regulation of GFAP in MGC (Fig 5A and B) and with reports of visual deficits in early childhood of USH patients (Stingl *et al*, 2019). Interestingly, the changes in subcellular morphology, the reduction in electrophysiological PRC function and the observed changes in visually guided behavior (Fig 2) come along with only very mild effects on overall retinal morphology (Fig 3E and Appendix Fig S9). It needs to be considered, however, that the retinal phenotype in USH1 patients varies widely, ranging from intact central retinal architecture and excellent visual acuity at 45 years to advanced central retinal damage and legal blindness by their early 30s (Lenassi *et al*, 2014). Such a phenotypic heterogeneity is observed in most if not all forms of inherited retinal diseases and is thought to arise from genetic or environmental confounding factors or a combination thereof (Veleri *et al*, 2015). The possibility to examine the USH1C pig model under standardized conditions will help to discern the effect of the mutation from other influencing factors.

In our work, we give also first indications for a potentially impaired hearing ability in heterozygous USH1C carriers (Figs 3A and EV2E and F). As the results were consistent at distinct ages, in different groups of animals and across the examined frequencies, we interpret this as a slight but significant biological effect. It must be considered that we are not aware of reports on impaired hearing in human USH1C carriers and so we cannot seriously estimate if these findings are of clinical relevance for human USH1C carriers. Taking into account the very low numbers of patients carrying distinct *USH1C* mutations, however, we would definitely exclude an evolutionary benefit of heterozygous *USH1C* defects, as it has been reasoned for the spreading of genes such as *HBB* (Piel *et al*, 2010) or *CFTR* (Gabriel *et al*, 1994; Pier *et al*, 1998) in the human population.

For the different aging processes in pig and human, a direct correlation of ages between the species is misleading. Instead, we propose to correlate the manifestation of fertility at 5–8 months in pigs, compared to 12–15 years in human beings, suggesting that a 12‐month‐old pig may fairly correspond to a biological age of 15–25 years in humans (http://www.age‐converter.com/, https://unleashpetphotography.com/animal‐age‐calculator). Accordingly, the phenotype in USH1C pigs appears to reflect patients well within the published spectrum of disease severity and progression. As a consequence, we would also suppose that translating clinically established endpoints (as measured e.g., by ERG or OCT) from human to pig will support the assessment of future pre‐clinical studies in our model. Vice versa, the multi‐disciplinary examination of the model described here defined novel disease hallmarks, which may give rise to alternative endpoints for future clinical trials. The value of additional read‐out parameters such as motion analysis (Fig 2) is corroborated by a pivotal study documenting the success of the *voretigene neparvovec* AAV‐based drug in the multi luminance mobility test (MLMT) as primary endpoint (Russell *et al*, 2017).

The specific wavelength sensitivity of cones (Kostic & Arsenijevic, 2016) and the complex interaction of vision with other sensory organs required substantial effort in the investigation of visually guided behavior. Parcour tests were conducted under distinct light conditions, but calculated luminance levels in the pig eye suggested that the experiments took place at the lower and upper limit of mesopic sensing (Zele & Cao, 2014). Albeit this prevents a clear discrimination of rod‐ and cone‐based vision in the motion analysis, essential conclusions can be drawn. First, the similarity of core parameters such as duration and snout contacts in the hurdle parcour between USH1C animals and WT controls in the dark confirms that pigs substantially rely on non‐vision sensing and denies an exclusive or dominant effect of vestibular dysfunction on movement. Second, the clear differences in illuminated environment and the characteristic difficulties that USH1C pigs have in the recognition of defined objects (Fig 2A–C) document their substantial problems in orienting within a structured 3D space. Although an influence of the bilateral vestibular dysfunction on orientation cannot be excluded (Kremmyda *et al*, 2016), the faster and smoother locomotion (Fig 2D) of WT control pigs, specifically under light conditions, demonstrates how a normal visual function promotes effective clearance of obstacles and “elegant” motion.

Taken together, the generation of an USH1C pig model has substantially improved our molecular understanding of the disease. We now have several lines of evidence how harmonin supports PRC function and the particular integrity of the OS. The early onset of basic defects in the retina and the impairment during the first year provides an ideal foundation for following the natural history of USH1C. Combining quantitative motion analysis with clinical examination tools will promote the definition of read‐out parameters in pre‐clinical studies and in clinical trials for treating the Usher syndrome, but also in trials involving patients with other progressive blinding or audiovestibular diseases. The long‐term maintenance of retinal structures and, moreover, the success of exploratory gene therapy approaches in older animals is highly relevant as it indicates a large window of therapeutic opportunity for USH1 patients.

## Material and Methods

### Regulatory statement

Work on USH pigs has been conducted under the supervision of the responsible regulatory authorities: the Regierung von Oberbayern has approved animal experiments involving somatic cell nuclear transfer, maintenance of pigs, and longitudinal monitoring at LMU Munich under the file number AZ 02‐17‐136. The State Veterinary Administration of the Czech Republic approved animal experiments on maintenance, sub‐retinal intervention and longitudinal monitoring at IAPG Libechov under the experimental protocol number 75/2019. Human primary cells were provided by University of Tuebingen, Germany, work on the cells was carried under approval of the ethics committee of the Landesaerztekammer Rheinland‐Pfalz under the file number 837.413.17. Informed consent was obtained from all subjects providing skin biopsies. The experiments on human fibroblasts conformed to the principles set out in the WMA Declaration of Helsinki and the Department of Health and Human Services Belmont Report.

### Antibodies, oligos, and gene synthesis

see Supplemental Materials and Methods.

### Generating USH1C pigs

#### Bioinformatics evaluation

*USH1C* loci were identified in macaque, marmoset, cattle, sheep, pig, horse, cat, dog, rabbit, mouse, and rat by BLAT using human exonic sequences and extracted from the respective reference genomes (www.ensembl.org). Multi‐species alignments were done by a combination of DiAlign and CHAOS (Brudno *et al*, 2004). Proposed regulatory elements were extracted from the USCS Genome browser (genome.ucsc.edu) as DNase sensitive elements (Thurman *et al*, 2012), ENCODE ChIP‐Chip (Wang *et al*, 2013), FANTOM5 enhancer elements (Andersson *et al*, 2014), GeneHancer elements (Fishilevich *et al*, 2017), FAIRE ENCODE regions (Giresi *et al*, 2007) and PreMod (Ferretti *et al*, 2007). Any identified regulatory element was localized within the alignment in BioEdit (https://bioedit.software.informer.com/7.2/) and a density plot was created. Both, a homology plot at nucleic acid level and a protein conservation plot were calculated in BioEdit on the basis of the nucleotide summary assessment and entropy calculation functions, respectively. Coverage maps of the augmented alignment were plotted by JalView (Waterhouse *et al*, 2009).

#### Personalized R31X allele

For introducing a patient‐specific segment, we analyzed a 1.6 kb fragment from an individual carrying an USH1C^R31X^mutation. The PCR product was amplified with the primers hush1c_2f and hush1c_2r, cloned into a plasmid vector and sequenced. For avoiding the misinterpretation of potential PCR mistakes, the sequence of the R31X‐allele was based on 6 distinct clones and the other allele on 3 distinct clones. Consistently appearing polymorphisms were defined as SNPs.

#### Targeting construct

The porcine USH1C gene has been annotated to chr2:42Mb, with the exons largely corresponding to the human USH1C transcript variant b3, GenBank no. NM_153676.4 (Appendix Fig S1). Distinct porcine primary cell lines (Richter *et al*, 2012) were examined for SNPs in the target region and 3 distinct gRNAs, rk1, rk3, and rk4 that were not affected by cell line‐specific SNPs were examined for their power to introduce NHEJ‐based mutations in pig primary cells lines; for this, cells were nucleofected and after 48 h the target locus was amplified with the primers ushwt1f and ushwt1r, cloned into plasmids and sequenced. Targeting experiments were based on rk4 as it proved superior (12% mutation rate) to rk1 (6%) and rk3 (10%). BACs CH242‐515C3 and CH242‐199J14, were identified to completely carry the porcine *USH1C* locus via the Sscrofa 10.2 reference genome and the PigPre BAC map (www.ensemlb.org) and purchased from CHORI BACPAC Resources Center (BACPAC Genomics, Emeryville, CA, USA). A sequence comprising a 278 bp 5′‐arm, the 1,524 bp humanized fragment including the TGA stop codon in exon 2 and 3 intronic SNPs as well as a 278 bp 3′‐arm were commercially synthesized and provided in a pUC plasmid derivate by GeneArt (Invitrogen by Thermo Fisher Scientific, Carlsbad, CA, USA). A cassette with a PGK/EM7 driven *neo* gene, providing neomycin resistance in mammalian cells and kanamycin resistance in bacteria (Klymiuk *et al*, 2012), was cloned into the plasmid via the *Not*I/*Xho*I sites located between the 5´‐arm and the humanized fragment. The entire fragment was excised from the backbone via *Asc*I and introduced into the CH242‐515C3 BAC by bacterial recombineering, according Vochozkova *et al* (2019) using the SW106 *E*. *coli* strain (Warming *et al*, 2005). Correct modification of the BAC was proven by PCR for recombination at the 5′‐ and 3′‐ends with the primer pairs 5arm1f – 5arm1r and 3armwf – 3arm2r, and for the removal of the corresponding porcine segment with the primer pair ushwt1f – ushwt1r. Further, the overall integrity of the BAC and changes in the restriction enzyme (RE) recognition sites that arise from to the modification were confirmed by the RE pattern after digestion with *Xba*I (Fig EV1C) and *Spe*I (Appendix Fig S1D).

#### Generating pigs

Plasmids carrying the modified BAC, the gRNA rk4 and Cas9 were prepared endotoxin‐freely and transfected into pig primary cells PKCf and PKCm by nucleofection (Amaxa by Lonza, Basel, Switzerland). Single cell clones were generated as described (Richter *et al*, 2012) and propagated toward a 2 × 96‐well scale. One aliquot was used for analysis by the loss‐of‐wild‐type‐allele approach (LOWA) (Vochozkova *et al*, 2019) and the other served as a backup for potential SCNT. qPCR was carried out on a LightCycler96 (Roche Life Science, Basel, Switzerland) using FastStart Essential DNA Green Master (Roche Life Science) and primer pair ush1c_qf1 – ush1c_qr1 for the target site as well as the primer pair o4_qf2 – o4_qr1 for a reference site in the *POU5F1* gene and ng_qf6 – ng_qr4 for a second reference in the *NANOG* gene. Verified single cell clones were used as donors in SCNT and embryos were transferred to synchronized gilts, according standard procedures (Kurome *et al*, 2015). Birth was introduced in pregnant foster mothers by Estrumate at day 115. (MSD Animal Health, Merck, Kenilworth, NJ; USA).

#### Tissue sampling

For isolation of pig primary kidney cells and sampling of tissue, animals were sedated with ketamine 100 mg/ml (Ursotamin^®^, Serumwerk Bernburg, Germany) and azaperone 40 mg/ml (Stresnil^®^, Elanco Animal Health, Bad Homburg, Germany), according to the manufacturer’s specifications. Fully anesthetized animals were euthanized by intravenous injection of T61^®^ (MSD Animal Health). Pig primary kidney cells were isolated according to standard procedures (Richter *et al*, 2012) (see below). Tissue samples for histology, electron microscopy, and molecular analyses were collected according to established sampling guidelines adapted to porcine biomedical models (Albl *et al*, 2016). Samples for molecular analysis were frozen on dry ice and stored at −80°C. Tissue was powdered using hammer and anvil first and pestle and mortar afterward. All instruments and samples were cooled in liquid nitrogen to avoid thawing of the samples during processing. Powdered tissue was transferred to a pre‐cooled tube and stored at −80°C.

#### Breeding

After reaching fertility and stabilization of cycle, USH1C F0 sows were inseminated with WT sperm. When pregnant, sows were trained and reassured intensively for farrowing by regular physical contact with caretaker. F1 offspring were genotyped (see below) and raised for breeding purposes. After reaching fertility, F1 boars were mated to their mothers and sisters for producing USH1C‐null pigs and with wild‐type sows to broaden the genetic background of the future breeding herd. Some litters from hom x het matings were with their mother for suckling, but piglets were kept in a separate box otherwise. Upon calling by the mother, the barrier between the boxes was opened and the time until piglets passed the gate was determined. Non‐responding piglets were nudged by the caretaker after one minute to avoid impaired nurturing.

### Molecular analysis

#### Genomic level

Genomic DNA was isolated from tail biopsies by using the Easy DNA kit (Invitrogen), DNAeasy kit (Qiagen, Hildesheim, Germany) or Nexttec kit (Nexttec GmbH, Leverkusen, Germany). For verifying the abundance of the modification element, amplicons generated with the primer pair hush_for3 – hush_rev3 were sequenced. The presence of the *neo* selection cassette was detected by endpoint PCR with usharm2f – ush5arm2r and sequencing of the amplicon while Cre‐mediated excision of *neo* was detected with neoFOR3 – neoREV3 and sequencing of the amplicon.

#### Transcriptional analysis

RNA was isolated from powdered tissue samples using TRIzol (Invitrogen). Approximately 100 mg of powdered tissue was grinded up in 1 ml of TRIzol by Polytron PT2500E (Kinematica, Luzern, Switzerland) in a pulsatile manner to avoid overheating. Further steps were carried out as suggested by the TRIzol protocol and RNA was stored at −80°C. For cDNA synthesis, samples were treated first with DNase I (Invitrogen) to remove a possible contamination with genomic DNA. Then, RNA was reversely transcribed into cDNA with SuperScriptTM III Reverse Transcriptase (Invitrogen). For determining USH1C transcripts, cDNA was used in RT–PCRs with the primer pair ushrt2f – ushrt2r or ushrt2f – urt4r, located in the consistently transcribed exons 1 and 8 or 6, for detecting all splice variants of the gene. Primer pairs ushrt11f – ushrt11r and ushrt13f – ushrt13r were used to amplify *USH1C_a* and *USH1C_c* variants (pig specific primers binding on exons according the splice pattern of human GenBank entries NM_005709.4 and NM_001297764.2, respectively). Primer pairs ushrt16f – ushrt16r and ushrt18f – ushrt18r were used to amplify *USH_b* splice variants (pig specific primers binding on exons according the splice pattern of human GenBank entry NM_153676.4). Afterward, Sanger sequencing was performed on PCR amplicons.

#### Protein analysis

Tissue was powdered and then processed as previously described (Sedmak & Wolfrum, 2011). In brief, retinal tissues were lysed in modified RIPA buffer (50 mM Tris–HCl, 150 mM NaCl, 0.1% SDS, 2 mM EDTA, 1% NP‐40, 0.5% sodium‐deoxycholate, 1 mM sodiumvanadate, 30 mM sodium‐pyrophosphate, pH 7.4). Protein lysates were separated by SDS–PAGE gel electrophoresis, followed by semi‐dry Western blotting as previously described in (Overlack *et al*, 2011). Western blots were analyzed and quantified in an Odyssey infrared imaging system (LI‐COR Biosciences, Lincoln, NE, USA).

### Morphology & histology

#### Immunohistochemistry

Porcine eyes were fixated in melting isopentane for cryosectioning as described (Overlack *et al*, 2011). Samples were sectioned with a MICROM HM 560 Cryo‐Star cryostat (Fisher Scientific by Thermo Fisher Scientific, Waltham, MA, USA) and placed on poly‐L‐lysine‐precoated coverslips. After drying, samples were incubated with 0.01% Tween 20 PBS, then washed with PBS and covered with blocking solution (0.5% cold‐water fish gelatin, 0.1% ovalbumin in PBS) for a minimum of 30 min followed by overnight incubation at 4°C with primary antibodies (for details on antibodies and fluorescent dyes see below). For GFAP staining samples were post‐fixated with 4% paraformaldehyde (PFA) in PBS. Cryosections were washed repeatedly and incubated with secondary antibodies in blocking solution containing DAPI (Sigma‐Aldrich, St Louis, MO, USA) for 1 h at room temperature. After washing, sections were mounted in Mowiol (Roth, Karlsruhe, Germany). Slides were analyzed on a Leica DM6000B microscope (Leica, Bensheim, Germany); images were processed with LAS‐AF Leica imaging software and ImageJ/Fiji software (Schindelin *et al*, 2015) or Adobe Photoshop CS (Adobe Systems, San Jose, CA, USA).

Quantitative analyses of connecting ciliary length and cone synaptic width: For measurements of connecting cilia length and width of cone pedicle synapses we stained cryosections through pig retinae with either GT335, a marker for tubulin glutamylation in the connecting cilium or FITC‐PNA, a marker for the extracelluar matrix of cones, respectively. For the image analysis, we developed Fiji scripts by ImageJ/Fiji software for automatic analysis of the fluorescent images.

The script for measuring connecting cilium length is:

run("8‐bit");
run("Smooth");
run("Auto Threshold", "method=Yen ignore_black white");
run("Analyze Particles...", "size=10–75 pixel display add").

The script for measuring synapse width is:

run("8‐bit");
run("Mean...", "radius=2 stack");
run("Subtract Background...", "rolling=50 stack");
//setThreshold(0, 11);
run("Convert to Mask", "method=Otsu background=Light black");
run(“Measure”).

#### Scanning electron microscopy (SEM)

The temporal bones of the pigs were dissected to expose the middle and inner ears. Their cochleae were dissected immediately and the organ of Corti was exposed. The tectorial membrane was removed prior to fixation. Dissected cochleae were fixated in 2,5% glutaraldehyde, 4% PFA in 0.1 M Sörensen’s phosphate buffer. After several washing steps with 0.1 M Sörensen’s phosphate buffer, cochleae were dehydrated in ethanol, critical‐point dried, and gold sputtered in an argon atmosphere. Specimen were imaged with a Philips ESEM XL30 scanning electron microscope (Philips, Eindhoven, Netherlands).

#### Transmission electron microscopy (TEM)

For conventional TEM dissected eyeballs were pre‐fixated for 2 h in buffered 2.5% glutaraldehyde containing sucrose and post‐fixated in buffered 2% OsO4 as previously described (Karlstetter *et al*, 2014). After dehydration in ethanol and passaging through propylenoxid as an intermedium, samples were embedded in Renlam^®^ M‐1 and polymerized at 60°C. For the pre‐embedding labeling, we followed a previously established protocol (Sedmak & Wolfrum, 2010). In brief, eyes were pre‐fixated in buffered 4% PFA, dissected, infiltrated with 30% buffered sucrose, and cracked by repeated freeze–thawing, followed by embedding in buffered 2% Agar (Sigma‐Aldrich). Agar blocks were sliced with a VT1000 S vibratome (Leica). Endogenous peroxidase activity in vibratome sections was suppressed by incubation with H_2_O_2_. Sections were incubated with primary antibodies for 4 days and overnight with biotinylated secondary antibody and were visualized with a Vectastain ABC‐Kit (Vector Laboratories, Burlingame, CA, USA).

Retina sections were first post‐fixated in 2.5% glutaraldehyde buffer and second in 0.5% OsO4 buffer. After dehydration, sections were flatmounted between ACLAR^®^‐films (Ted Pella Inc., Redding, CA, USA) in Renlam^®^ M‐1 resin. Flatmount samples were heat‐polymerized and glued on the top of empty Araldit blocks. Ultrathin sections of the embedded specimens were prepared with an Ultracut S ultramicrotome (Leica) and collected on Formvar‐coated copper or nickel grids. Sections were counter‐stained with heavy metals before imaging and analyzing in a Tecnai 12 BioTwin TEM (FEI by Thermo Fisher Scientific, Hillsboro, OR, USA), equipped with a SIS Mega‐View3 CCD camera (EMSIS, Münster, Germany) was used. The images were processed using Adobe Photoshop CS (Adobe Systems). Connecting cilia length or synapse width were analyzed directly in the TEM using the measuring tool of the Olympus SIS image analysis system (Soft Imaging Systems, Muenster, Germany). Quantification steps have been performed with Fiji/ImageJ.

#### Primary cell culture

Pig primary kidney cells and primary dermal fibroblasts were isolated according to standard procedures (Richter *et al*, 2012). In brief, kidneys were taken from euthanized animals, washed in PBS containing Penicillin/Strepomycin and minced. Tissue was treated with collagenase and filtered cells were cultivated in DMEM. Pieces of porcine skin were disinfected in 98% Ethanol and after short washing in PBS placed in 10% Betaisodona solution. After repeated washing steps in PBS, skin was minced. Kidney or skin samples were transferred to cell culture dishes and completely covered with MEM containing 10% heat‐inactivated fetal calf serum (FCS) and 1% penicillin‐streptomycin (PS) and incubated at 37°C and 5% CO_2_. 5 days later 10% FCS were added. Outgrown cells were transferred to new complete medium for further culturing. For ciliogenesis experiments, 5 × 10^5^ cells were seeded per well (in medium containing 10% FCS and 5% PS) and 24 h later cultured in OPTI‐MEM reduced‐serum medium (Invitrogen by Thermo Fisher Scientific). After 48 h of starvation, cells were washed in PBS and fixed with 2% PFA in PBS. After washing and permeabilization with 0.1% Triton‐X in PBS, cells were blocked with 0.5% cold‐water fish gelatin, 0.1% ovalbumin in PBS followed by incubation with primary antibodies overnight at 4°C. After washing, coverslips were incubated with secondary antibodies for 1 h at room temperature. Samples were washed and then mounted in with Mowiol (Roth). The primary ciliary length and number of ciliated fibroblasts were quantified by using Fiji. Lengths were measured directly in immunostained images taken with a 63‐x objective. For this purpose, the Arl13b‐positive axoneme was measured from the tip to the cilia base, which was labeled for pericentrin, a common marker for the ciliary base.

### Clinical examination

#### Anesthesia

For examination with ERG, mfERG, OCT, AF, FA, and ABR on 3‐week old animals were anesthetized by intramuscular injection of 2 mg/kg azaperone (Stresnil^®^, Elanco, Germany), 0.02 mg/kg atropine sulfate (B. Braun, Germany) and 20 mg/kg ketamine (Ursotamin^®^, Serumwerke Bernburg, Germany). Anesthesia was continued by intravenous injection of propofol (Fresenius, Germany) according to effect. After endotracheal intubation, pigs were mechanically ventilated and cardiovascular function was monitored throughout the procedures. To exclude eyeball movement during examinations, a peripheral muscle relaxant (Rocuronium, Inresa, Germany) was applied. For ABR on older animals, animals were starved overnight before intramuscular application of TKX (tiletamine 4 mg/kg, zolazepam 4 mg/kg (Zoletil 100; Virbac), ketamine 5 mg/kg (Narketan 10; Chassot), and xylazine 1 mg/kg (Rometar 2%; Spofa).

#### Auditory brain stem response (ABR)

In 3‐week‐old USH1C and WT piglets, ABR was recorded with standard electrodiagnostic equipment (Viking Quest^®^; Natus, Planegg, Germany) and Natus^®^ TIP‐300 insert earphones (AF). Recording stainless steel needle electrodes were positioned subcutaneously ipsilateral to the stimulated ear over the mastoid at the base of the ear (−) and at the vertex in the midline of the skull corresponding to Cz (+). A ground electrode was positioned in the neck. Electrode impedance was < 2 kOhm. Click stimuli (100 µs, alternating polarity, 11.1 Hz) were delivered at supramaximal stimulation intensity (100 dBSPL) to one ear and masking noise was applied to the contralateral ear (−40 dBSPL). Filter settings were 100 Hz for high pass and 3 kHz for low pass filters. The ABR represented the averaged signal of 1,000–1,500 10 ms recordings. ABR was recorded twice for each ear to ensure reproducibility of the ABR peaks in WT animals (*n* = 3) or absence of recognizable peaks in USH1C‐null animals (*n* = 3), respectively. If there was no recordable ABR at 100 dBSPL in USH1C‐null animals, ABR was also conducted at 120 dBSPL (twice for each ear). Additional testing comprised ABR at decreasing steps of 10 dB in WT piglets. Hearing threshold was determined as the minimal click intensity that still evoked a noticeable potential negative peak after peak V. ABR for other time points were conducted with four subcutaneous needles placed in vertex, forehead, and mastoids with specialized audiometric equipment. 8 weeks and 2 years old animals were examined with eABRUSB (eABR–BioMed Jena GmbH, calibrated by standard norm IEC 318‐4) and headphones (Sennheiser Momentum M2 In‐Ear G Black‐Red, 18Ω) were used on the 8 weeks old piglets. Click stimuli (duration 100 µs, steps 10 to 5 dB) and tone pips (duration 100 µs, frequencies 2, 4, 8, 16 kHz, steps 10 to 5 dB) were applied. The evoked signal was filtered by band‐pass filter of range from 300 Hz to 3 kHz. All the responses were averaged 200–400 times for each intensity and analyzed with eAudio software. On 3 months, 6 months and 2 years old pigs, acoustic stimuli were generated with an evostar 2 system (ERA system: Evoselect/Evostar 2, Pilot Blankenfelde medizinisch elektronische Geräte GmbH) and presented to the animal via headphones (beyerdynamic, DT48, 5Ω, Germany). Click stimuli (duration of electric pulse 150 µs) and the tone pips (duration 2 ms, frequencies of 0.5, 1, 2, 3, 4 kHz, intensity steps from 10 to 5 dB) were applied. The signal from an electrode was filtered by band‐pass filter over the range of 100 Hz to 2.5 kHz. The response was averaged 200–500 times on each intensity. This signal was processed with and analyzed using Evostar software. The threshold response to each frequency and click was determined as the minimal tone/click intensity that still evoked a noticeable potential peak in the expected time window of the recorded signal. This evaluation process was the same for both systems.

#### Electroretinography (ERG)

Full‐field ERG was recorded with a corneal Kooijman electrode (Roland Consult, Brandenburg an der Havel, Germany). Platinum needles were placed subcutaneously at the temple and top of the head between the ears as reference and ground electrodes respectively. Stimuli were brief white flashes (4 ms) delivered from a light source within the Kooijman electrode (Kooijman & Damhof, 1986). The RETImap system (Roland Consult) was used for stimulus generation and data acquisition. The recording protocol used was based on, and extended from, the International Society for Clinical Electrophysiology of Vision standard for human clinical full‐field ERG. A band‐pass filter was used to remove frequencies outside 1–300 Hz. An interstimulus interval (ISI) of 1 s was used with 20 responses recorded and averaged for each single flash protocol. For scotopic ERG, the protocol consisted of 30 min dark‐adaptation, followed by recording of a dark‐adapted single flash series with increasing luminance from −3.0 log cd*s*m^−2^ to 2.0 log cd*s*m^−2^. After completion of the dark‐adapted intensity series, animals were light adapted for 10 min to a steady white background of 30 cd*s*m^−2^ with the same light source. Light‐adapted standard flash was recorded by using white flash stimuli at 0 log cd*s*m^−2^. The same luminance was used to record a 28 Hz flicker response for 300 ms. For multifocal ERG, the mfERG was recorded using the same RETImap system (Roland Consult) using a 61‐hexagon stimulus and according to the standard of the International Society for Clinical Electrophysiology of Vision (Hood *et al*, 2012). A 61‐hexagon stimulus was presented on a 51‐cm cathode ray tube monitor with a frame frequency of 60 Hz. It encompassed the central visual field of 44° horizontally and vertically. The scaled‐size hexagons (distortion factor 4) were light‐modulated according to a binary pseudorandom m‐sequence. The light state had a luminance of 120 cd/m^−2^ and the dark state of 1 cd/m^−2^. Refractive errors were not corrected. Pupil dilation was achieved with one drop of tropicamide–phenylephrine mixture into the conjunctival sac of each eye about 15 min before the exam. Contact lens electrodes (ERG‐jet^®^, CareFusion, San Diego, USA) were applied. Amplifier gain was set to 50,000 and the band‐pass from 5 to 100 Hz. There were eight recording cycles of 47 s each. The 61 traces are displayed separately in a false‐color‐coded amplitude map identifying P1 amplitude (measured from the N1 trough to the following maximum positive peak; N1 amplitude was measured from the electroneutral starting point to the first negative deflection). All traces were also grouped into one response value and the averaged first‐order kernel function displayed as single trace. Luminance levels in the pig eye were calculated from the light intensity measured at the floor. Given the complex physical correlation between light source, reflection, and sensing in an indoor 3D space, it is challenging to determine corresponding luminance levels in the pig eye, but considering the general reflexion parameter ρ as 0.2 seems a fair approximation.

#### Optical coherence tomography (OCT)

Spectral‐domain OCT B‐scans of central retina in animals were obtained using a Spectralis HRA + OCT device (Heidelberg Engineering, Heidelberg, Germany) under general anesthesia and during full pupil dilation. During the acquisition, the corneal surface was protected with methylcellulose eye drops while lids were held open using lid specula. After 30deg and 55deg infrared and red‐free images were recorded using scanning laser ophthalmoscopy (SLO), animal received a flush of approximately 5 mg/kg fluorescein and the angiography was recorded using the 55 degree lens at multiple time points (0.5, 1, and 5 min).

### Behavior testing

#### Barrier course

The design was adapted from a previous publication (Barone *et al*, 2018). Ten barriers (boards of 94 × 76 cm) were placed in a distance of one meter in a straight aisle (12 × 2.2 m) alternating in the middle, the left or right positions. There were four different arrangements of barriers, which were used alternatingly (Appendix Fig S4). Pigs were trained in the course until they walked straight through the course without pausing or hesitating for at least two times in a row. After a training period of four weeks, the animals were tested in the course weekly. The pigs entered the course individually in random order, they had to pass the barriers, and at the end, they were rewarded with fruit juice in a food bowl. The test was performed before feeding to ensure a higher motivation in the pigs. After some runs in the light, the test was conducted alternately in the light (average of 135 lux) and in the dark (average of 2.9 lux). Illumination level was measured at three different sites in the course (start, middle and end) in animals’ head height. Each run was documented by video. The pigs were marked with numbers on their backs to facilitate recognition. The times pigs took for passing the course, from snout crossing the start line until the pig was touching the food bowl, were measured. All barrier contacts by the pigs were counted. Contacts were divided into two subgroups. Frontal contacts mean the pig was touching the barrier with its snout or head, while contacts were called lateral when the pig was touching the obstacle barrier with its shoulders or hips. Each turning around or walking back before reaching the end was counted. It was distinguished between turns in front of the course and turns within the course. Such runs were excluded. After turning around, animals were sent back to the course, and the new run was documented with time and contacts when they reached the end. Walking in circles was counted as a separate event as well. As long as they were just circling and not walking back, the run was not excluded, time measurement was continuing. When the pig was circling in front of the start line, those circles were also counted, but time measurement only started when the animal was crossing the start line. Pigs were excluded from parcour testing when they were lame or when they had already been outside of their pen on the experiment day for another reason. When a pig was turning around or circling so often that it needed longer than two minutes to walk to the endpoint, the run was also excluded. The numbers of turns/circles within those two minutes were nonetheless counted. Pigs were not included in the study when motivation was lacking, for example, in the case a pig did not head to the endpoint reliably after six weeks of training or when it seemed more interested in playing with the barriers than walking straight to the food bowl.

#### Obstacle course

An obstacle course to test the visual capacity of USH1C pigs and WT pigs was conducted. The course was 6.15 m long, the width was 1.58 m in the beginning and 2.48 m in the end. Most obstacles were built from obstacles used for equestrian sport, other obstacles were a stepboard and barrels. Obstacles were rearranged for almost each test day (Appendix Fig S5).

Pigs were trained until they walked straight through the obstacle course to the food trough without stopping or hesitating. After a training period of three weeks, the pigs were performing the course once a week. The pigs entered the course individually in random order, they had to pass the obstacles and at the end they were rewarded with a cookie in a food trough. The test was conducted under light and dim conditions. Each run was documented by video. The pigs were marked with numbers to facilitate recognition. The times the pigs needed to pass the course were measured. All contacts with the obstacles or the wall were counted. When a pig nudged one obstacle more than once in a playful way in the same area, this was counted as one touch. When a pig touched one obstacle at different sites, each contact was counted separately. When a pig swept along an obstacle with its snout, this was counted as two touches. When a pig turned around before reaching the end or when a pig was lame, the pig was excluded for the test day. For analyzing the trajectory, the dimensions of the course and the gaps of the slatted floor were measured and scaled at 1:100 (Macromedia FreeHand MX, STUDIOMX 2004). Using the slatted floor as a ruler, each footstep of each animal in each qualified run were plotted in the map. X and Y coordinates of footsteps as well as the corresponding times when the foot touched the ground were transferred to an excel file. Trajectories were analyzed from these data sets with the R package trajr. Briefly, we first imported and smoothed all trajectories to avoid any source of noise by applying a Savitzky‐Golay smoothing filter (Savitzky & Golay, 1964; Luo *et al*, 2005). Then, we called all the functions of the trajr packages (McLean & Skowron Volponi, 2018) to characterize the trajectories into measures of speed, length, distance, duration and measures of straightness or tortuosity. The trajectory significance was tested using Kruskal–Wallis test. For statistical evaluation, data were checked for consistency and normality. Fisher’s exact test or Pearson’s test were used to analyze cross tabulations. Generalized linear models with Poisson distribution, Median tests, bootstrap‐t tests based on 5000 Monte Carlo simulations, *t*‐tests with and without the assumption of homogeneity, Mann–Whitney U‐tests were used to test continuously distributed variables. All reported tests were two‐sided, and *P* < 0.05 were considered statistically significant. Analyses were performed by use of NCSS_10 (NCSS LLC, Kaysville, UT, USA), STATISTICA_13 (Statsoft, Tulsa, OK, USA), MATHEMATICA_12 (Wolfram Research, Hanborough, UK), Champaign, IL (2018) and PASW 24 (SPSS by IBM Corp, Armonk, NY, USA), and Prism_5 (GraphPad Software, San Diego CA, USA).

### Therapy approaches

#### AAV transduction of retinal layers

Distinct AAVs (AAV8.CMV.eGFP, AAV9.CMV.eGFP, and Anc80.CMV.eGFP) were generated by the viral core facility at Boston Children’s Hospital Viral Core at the Massachusetts Eye and Ear Infirmary. AAVs were injected subretinally into the eyes of WT pig at an age of 30 months using an ophthalmologic surgery microscope (Hi‐R NEO 900A, Haag‐Streit). Using inhalation anesthesia (Morpheus E, Siare), morphology of the retina was examined via OCT iVue (Optovue, Fremont, CA, USA) on both eyes before the injection. 200 µl of AAVs (2 × 10^10^ AAV particle (vg)) were applied in the subretinal space with Extendible 41G subretinal injection needle (23 gauge/0.6 mm) (DORC, Zuidland, Netherlands). After 5 weeks injection sites were evaluated by OCT (see above) and funduscopy, confirming the concise injection sites. Following enucleation, the eyecup was dissected and fixed with 4% PFA in PBS. Flattened whole mount preparations of the eyecup were analyzed by epifluorescence using a handhold device (Bluestar Flashlight, Nightsea, Lexington, MA, USA). Samples were processed for cryosectioning and analysis as described above.

#### Gene repair *in vitro*

Different constellations of the gRNAs urg1 and urg2 with correcting ssODN urt1, urt2, urt1.1, urt1.2, urt1.3, urt1.4, and urt1.5 were tested in primary fibroblasts. For this, plasmids encoding Cas9 and the respective gRNA as well as correcting oligonucleotides were nucleofected under standard conditions (Richter *et al*, 2012). After 24–48 h of cultivation, DNA was isolated from mixed cell batches and the efficacy of NHEJ and HDR were determined by examination of Sanger electropherograms of amplicons generated by PCR with the primer pair huUSH2f – huUSH2r and sequenced with primer ush1s in the web‐based ICE v2 CRISPR Analysis Tool (https://www.synthego.com/products/bioinformatics/crispr‐analysis by Synthego, Menlo Park, CA, USA). For examination of specific allelic constellations, cells nucleofected with urg1 and urt1.3 were seeded onto 96‐well plates and single cell clones were produced. DNA was isolated from independent clones and PCR amplicons were generated by primer pair ushHS1f – huUSH1r, sequenced with primer ush1s, and analyzed by Sanger sequencing.

#### Gene therapy *in vitro*

For restoring gene expression, 2.5 × 10^5^ primary porcine fibroblasts were transfected with 0.4 µg of an endotoxin‐free pDest_Harm_a1_S/F plasmid (Invitrogen), expressing splice form a1 of the human USH1C gene under the control of the CMV promoter. Transfection was performed with the 4D‐NucleofectorTM X Unit (Lonza), using the P2 Primary Cell 4D‐NucleofectorTM X Kit S and program FS113, according to the manufacturer’s protocol. After transfection, cells were seeded in 10% FCS and analyzed for ciliogenesis as described above.

#### Gene therapy *in vivo*

1 × 10^11^ vg Anc80.CMV.harmonin_a1 in 200 µl PBS (manufactured by Boston Children's Hospital Viral Core) was injected subretinally into the right eye, while the left eye was sham‐treated with 200 µl PBS vehicle, according to the procedures described for the AAV transduction experiment (see above). Animal #10056 was injected at an age of 2.5 years and experiment was terminated after 12 months after gfERG examination. Eyes were collected from the vesicular and non‐vesicular areas of the retina for immunofluorescence and Western blot analyses. Animal #10054 was injected at an age of 3.5 years. After intermediate gfERG examination 3 months after injection, the experiment is ongoing.

## Author contributions

**Sophia Grotz:** Validation; Investigation; Visualization; Methodology; Writing—original draft; Writing—review and editing. **Jessica Schäfer:** Validation; Investigation; Visualization; Methodology; Writing—original draft; Writing—review and editing. **Kirsten A Wunderlich:** Investigation; Methodology. **Zdenka Ellederova:** Methodology; Project administration. **Hannah Auch:** Validation; Investigation; Methodology. **Andrea Bähr:** Supervision; Investigation; Methodology. **Petra Runa‐Vochozkova:** Supervision; Investigation; Methodology. **Janet Fadl:** Investigation. **Vanessa Arnold:** Investigation. **Taras Ardan:** Investigation; Methodology. **Miroslav Veith:** Investigation; Methodology. **Gianluca Santamaria:** Software; Formal analysis; Methodology. **Georg Dhom:** Investigation. **Wolfgang Hitzl:** Formal analysis; Methodology. **Barbara Kessler:** Methodology. **Christian Eckardt:** Investigation. **Joshua Klein:** Investigation. **Anna Brymova:** Investigation. **Joshua Linnert:** Investigation. **Mayuko Kurome:** Methodology. **Valeri Zakharchenko:** Methodology. **Andrea Fischer:** Supervision; Investigation; Methodology; Writing—review and editing. **Andreas Blutke:** Investigation; Methodology; Writing—review and editing. **Anna Döring:** Investigation. **Stepanka Suchankova:** Investigation; Methodology. **Jiri Popelar:** Supervision. **Eduardo Rodríguez‐Bocanegra:** Validation. **Julia Dlugaiczyk:** Supervision. **Hans Straka:** Formal analysis. **Helen May‐Simera:** Conceptualization; Investigation; Methodology; Writing—review and editing. **Weiwei Wang:** Resources. **Karl‐Ludwig Laugwitz:** Conceptualization; Methodology; Writing—review and editing. **Luk H Vandenberghe:** Resources. **Eckhard Wolf:** Conceptualization; Methodology; Writing—review and editing. **Kerstin Nagel‐Wolfrum:** Conceptualization; Validation; Investigation; Visualization; Methodology; Writing—original draft; Writing—review and editing. **Tobias Peters:** Formal analysis. **Jan Motlik:** Supervision; Methodology; Project administration; Writing—review and editing. **M Dominik Fischer:** Supervision; Validation; Visualization; Methodology; Writing—original draft; Writing—review and editing. **Uwe Wolfrum:** Conceptualization; Supervision; Funding acquisition; Validation; Investigation; Visualization; Methodology; Writing—original draft; Writing—review and editing. **Nikolai Klymiuk:** Conceptualization; Supervision; Funding acquisition; Validation; Investigation; Visualization; Methodology; Writing—original draft; Project administration; Writing—review and editing.

In addition to the CRediT author contributions listed above, the contributions in detail are:

SG contributed to molecular analysis and breeding of animals, designed and performed behavior tests and contributed to writing of the manuscript. JS contributed to molecular and morphological analysis, and ciliation analysis in primary fibroblasts. KAW contributed to tissue sampling and processing, imaging (IF and SEM), data analysis and interpretation. ZE contributed to breeding attempts sub‐retinal intervention and ABR measurements. HA evaluated Gene Repair. ABä managed intervention on pigs and anesthesia. PR‐V contributed to Gene Repair study. JF contributed to genetic modification. VA contributed to quantitative analysis of the synaptic phenotype in cones. TA managed intervention on pigs. MV performed subretinal injection. GS performed trajectory analysis. GD contributed to animal management and tissue sampling. WH performed data analysis. BK contributed to Somatic Cell Nuclear Transfer and performed Embryo Transfer. CE performed RT–PCR on USH1C transcripts. JK participated in the molecular and morphologic analysis of eyes after USH1C gene therapy. ABr contributed to ERG measurements. JL performed ciliation analysis in primary fibroblasts. MK and VZ performed Somatic Cell Nuclear Transfer. AF, SS, and JP performed ABR measurement. ER‐B participated in ophthalmologic analysis. ABl conducted pathological examinations and tissue preparation. AD contributed to ERG measurement. JD and HS contributed to the analysis of the vestibular phenotype. HM‐S assisted with tissue dissection and cochlear preparation. K‐LL and EW contributed to the design of the study and the writing of the manuscript. WW and LHV provided AAV vectors. KN‐W contributed to the design of the study, interpretation of data and writing of the manuscript. TP performed ERG and OCT measurements. JM contributed to the design of the study, orchestrated breeding attempts, sub‐retinal intervention and ABR measurements. MDF led the ophthalmological characterization of animals, performed the vitreoretinal surgery and contributed writing of the manuscript. UW designed and coordinated the study, performed molecular and morphological analysis, interpreted data and wrote the manuscript. NK designed and coordinated the study, performed bio‐informatic and molecular analysis, interpreted data and wrote the manuscript. ER‐B contributed to analysis of ERG and OCT measurements.

## Supporting information



Appendix

Expanded View Figures PDF

Movie EV1

Movie EV2

Movie EV3

Movie EV4

Movie EV5

Movie EV6

Movie EV7

Source Data for Figure 1

## Data Availability

This study includes no data deposited in external repositories.
