# Supplementary material for: Early disruption of photoreceptor cell architecture and loss of vision in a humanized pig model of usher syndromes
Source: EMBO Mol Med. 2022 Mar 7;14(4):e14817. doi: 10.15252/emmm.202114817 (PMC8988205; doi:10.15252/emmm.202114817)
Supplement: Supplementary file 1 — Appendix [file EMMM-14-e14817-s004.pdf]

Appendix to “Early Disruption of Photoreceptor Cell Architecture and Loss of Vision in a Humanized Pig Model of Usher Syndrome” by *Grotz et al.*

Content:

Supplementary Materials and Methods 2

Appendix Table S1: Experimental animals. 5

Appendix Figure S1: Modification strategy. 6

Appendix Figure S2: Breeding of USH1C pigs. 7

Appendix Figure S3: Transcriptional evaluation of heterozygous F1 animals. 8

Appendix Figure S4: Extended examination of barrier course (Figure 2A). 9

Appendix Figure S5: Set-up of runs in obstacle course (Figure 2B). 10

Appendix Figure S6: In-depth statistical analysis of obstacle course (Figure 2B). 11

Appendix Figure S7: Individual variability of pigs in obstacle courses. 11

Appendix Figure S8: ERG data of USH1C and WT pigs at 1 year of age. 12

Appendix Figure S9: Intact retinal and choroidal vasculature. 13

Appendix Figure S10: USH1C splice variants. 14

Appendix Figure S11: GFP-expression in cells of the retinal pigment epithelium (RPE). 15

Appendix Figure S12: USH protein conservation. 16

Supplemental materials & methods

Antibodies & Fluorescent dyes:

The following primary antibodies were used in this study:

- rabbit polyclonal antibody against GFAP (#ZO334, purchased from DAKO by Agilent, Santa Clara, CA, USA); WB 1:10000, IF 1:1000
- affinity-purified rabbit polyclonal antibody against harmonin (H3) (homemade as in (Reiners et al., 2003)); WB 1:1000, IF 1:500
- rabbit polyclonal anti-Arl13b (#PTG 17711-1-AP, purchased from Acris by Origene, Rockville, MD, USA); IF 1:400
- goat polyclonal anti-pericentrin 2 (#sc-28145, purchased from Santa Cruz Biotechnology, Dallas TX, USA); IF 1:200
- mouse monoclonal antibody against Cent3 (homemade as in (Trojan et al., 2008); IF 1:100
- guinea-pig polyclonal anti-whirlin (homemade as in (van Wijk et al., 2006)); WB 1:5000
- rabbit polyclonal antibody against SANS (homemade as in (Sorusch et al., 2017)); WB 1:500
- rabbit polyclonal anti-myosinVIIa (#PTS-25-6790-CO50, purchased from Axxora by Enzo Life Sciences, Farmingdale, NY, USA); WB 1:1000
- mouse monoclonal antibody against GT335 (AdipoGen Life Sciences, (SanDiego, CA, USA); IF 1:1000
- mouse monoclonal anti-actin (#Sct MA5-11869, purchased from Invitrogen); WB 1:2000
- secondary antibodies used in this study were conjugated to Alexa488, Alexa 555, Alexa568, or Alexa 647, purchased from Invitrogen or Rockland Immunochemicals (Pottstown, PA; USA). For staining of cone pedicle synapses, lectin PNA (Peanut agglutinin) was coupled to Alexa 568. Nuclear DNA was stained with DAPI (4',6-diamidino-2-phenylindole) (1 mg/ml) (Sigma-Aldrich).

Oligo & Gene Synthesis:

Primers (with RE recognition sites underlined):

Genotyping:

- hush1c_2f: 5´-CTTAGGATCCAGGGAAGGAGGTACTGTCAGATCTAGG-3´
- hush1c_2r: 5´-TAAGCTCGAGACCACAAGTTACTCAAGGAAAGA-3´
- ushwt1f: 5'-TTCCCTCCTCTTCTCACAACCAT-3'
- ushwt1r: 5'-AGAGCCACGCACAGCACCTCT-3'
- 5arm1f: 5´-GGCAAGCTGTCCAGGTAGA-3´
- 5arm1r: 5´-TGCTTCACTGCTCCCAGAT-3´
- 3arm2f: 5´-TGTTGCCGTGAGCTGTG-3´
- 3arm2r: 5´-GACGCCCTAACACTGTGC-3´
- hush_for3: 5´-TAGTTGCCAGCCATCTGTTGT-3´
- hush_rev3: 5´-CTGAAGCCAGACAGGAAACCT-3´
- ushwt2f: 5'-CATGGTCGGCTCATGT-3'
- ushwt2r: 5'-TTGAACCTCTCGGACTATCTG-3'
- neoFOR3: 5'-GCAAGCAAGGCAGGAT-3'
- neoREV3: 5'-GTATCCCAAGGGTGGAAG-3'
- ush5arm2f: 5'-GGGTTCTGTTGGTGAG-3'
- ush5arm2r: 5'-CTAGCTTGGCCGTAAA-3'

qPCR:

- o4_qf2: 5´-CAGGTAGGTTAGGCTGATAG-3´
- o4_qr1: 5´-CAAGTTCATGAACGGCAGAAC-3´
- ng_qf6: 5´-GAGGCTTCACTTGTTAAGGG-3´
- ng_qr4: 5´-CTGAGATCAGGGTAGACATAC-3´
- ush1c_q1f: 5'-CTTCTTGGAGCAGATGGGATAAA-3'
- ush1c_q1r: 5'-GAATGCAGATTTCTGGTTTCCAC-3'

RT-PCR:
- ushrt2f: 5´-CGGAGCCTGAAGGAGCGAG-3’
- ushrt2r: 5´-TTGTTTTCCCGACTGCCAGA-3’
- urt4r: 5’-ACAGGGATCAGGCCAATGTG-3’
-ushrt11f: 5’-GGCATATTCATCAGCCAGGT-3’
-ushrt11r: 5’-GACGGCAATAACAAGGTCAATC-3’
-ushrt13f: 5’-GACAGGGGACCAGATTGTCG-3’
-ushrt13r: 5’-CCTGCTTTAGGGCAAGGGAG-3’
-ushrt16f: 5’-GAGAACTGGGAGGCAAAGAA-3’
-ushrt16r: 5’-ACAGAGTCAGAGGAAGGACA-3’
-ushrt18f: 5’-CATGAGTGCCTCCTCTTCGC-3’
-ushrt18r: 5’-GTTCTCTGGCTTTTAGGGGTGA-3’

Gene Repair:
- ushHS1f: 5’- CCATGAAACTGACTAGTGG -3’
- huUSH2f: 5’- CCTTGCTCTGTTACCCGTTC -3’
- huUSH2r: 5’- GTTCTGTCCCAACAATCATGC -3’
- huUSH1r: 5‘- CTGGCATTTCTTGTTCTGT -3‘

(internal) sequencing primers:
- ushrt2f: 5´-CGGAGCCTGAAGGAGCGAG-3’
- ushrt2r: 5´-TTGTTTTCCCGACTGCCAGA-3’
- ushrt1s: 5´-GGTGTCAGCTGGTCGTACTCC-3’
- Ss9for: 5´- CTCAACTAATCGTGGCCTAGTG-3´
- ss11for: 5´- AGACAAGGAACTCTAATGCAAGT-3´
- ush1s: 5’- GTGCCTGGCCACATCTGGA -3’
- ush1s: 5’- AGATACCGGAAGGAGATG-3’

gRNAs (PAM underlined, cutting site in capital letters):
rk1: GagacatattcactaacTGtgGGG
rk3: GggaagtgacaggtgaTCtgGGG
rk4: GaggccctgatcctaccAAggAGG
urg1: GgacccagcacacttaCTggTGG
urg2: GtaccaccagtaagtgTGctGGG

Gene synthesis (bold capital letters are added recognition sites for *Asc*I, *Not*I, *Xho*I, *Mun*I restriction enzymes; the exon 2 coding sequence is in capital letters with the nonsense causing T shaded in gray):

GGCGCGCCgctttgataagttaaggcagggaatttggatttgaattttgttttaagtccagtgagaatctactaagcaagcaagggacaggat
ccaatttatttaaatatttctgggccctgatcctagggaacttcagattgtttttcctgctcgaactcctccgccacaaccaaatgcaggcagctcag
gcctgtttgagtgcgattcctcacacttctcgcacaggcccaggcaaatccaggctgggtcaccatcgagatggcttttggaggtg**GCGGCCG**
Cgcaaca**CTCGAG**agggaaggaggtactgtcagatctaggccagaaatctgcattctgtacccccctgctcaggccagaaatcccaagggctg
ggcccagcatgtcccctctgtggtgggacggacagactgccccggtcttccagaacccttgggatacccacagaaagaggtaacgctgctctggc
cctcttctgaggacgagtcagtggagagcatgcagcttccagctgcagcctctctatgaagggctgaggccctgggccgggaggctggaggaga
gagggacccagtgacccccccaagcttccaccttgctctgttacccgttcttgggctgaagagagacccaaaaatacagtgtagagattcacactg
aggtaactcagggagtggaattcagggcctcccgctgggattgaggtgctaatgacacaactcctgaacctgaccttagagtgccagccattgac
gtcaacaaagttgaaatgatgtaacctgacgctccccctgcggggcttgtgcaggggcctggggagggggaaggagtggccatgaaactgacta
gtggacagaacccagctaaggtcaggacaagacagagtgaaggtcccctggcactgatgttacagaagaattcggtggtaaggggcttctgga
gagtggcatgtgctatctaagcgagtggcccaaatccttcctgaaagcatttatccggcactacagccaccatcaggtaagacagtgggcttcttc
tggccatggatgacacagccatgggggtgagcagcagcactgccatggcagcgtgtcactgtcacatggggattcacatatgtacctatgtgtgtt
catccccgtgtgtgcacatattgccccacctggggacaaagggtgcctggccacatctggaggggcagcggtactcctgtggccacgttggggtg
gtctgcataggtctgatgcattggggtcagaggggcagcctggcctgtggctcctcttctctcctcacaactccagccctgaaaagctgctgggga
ggcccttggggatgacctctcctccctgaggtctgctatgggggcgggtgctgagcctggagctgtgattctgctattggattttccagGTGGAT
TTTCTGATTGAAAATGATGCAGAGAAGGACTATCTCTATGATGTGCTG**T**GAATGTACCACCAgtaagtgtgctgggtc

cagctcttgtgggccacttgggttcctttgtcttcagggagccctgggatgggttgttctgagacagaggagctcagagggtggatgctcacggctc
ctggaaatcaaatggacataccattcactcatttcagcaactatttacacaagtactttgtacttggctttgtactaggggctgggtatagttgtgag
ccagacagattggtctctgttttcaggttgctcacagtctgatggaggaggctgtctagtagccagatagattctatagagcatgattgttgggaca
gaacaagaaatgccagctggccacagcccttgcatcagatgtctccgatcacccacttgctttttga**CAATTG**gatgggccaagagtgggcaa
gtcagctggcaggtagagaagtgccagctgcagatgttgagggtcttaatgatgtgaagctgaggctggtgggagattgtgtgagggtgggattt
gccgagctccactgcagctcagacgcaggaaccaaggggatgagagcattcaccaccctctcgcagaggtttcctgtctggcttcagaactaggc
agagctgagtttgaatcctggcttgatttcatctgcagatttccttgggatacttaaac**GGCGCGCC**

Therapeutic ssODN (with the correcting nucleotide shaded in grey and a blocking nucleotide, preventing repeated cutting by the gRNA in underlined capitals):

urt1: (anti-sense orientation):
5´- caacccatcccagggctccctgaagacaaaggaacccaagtggcccacaagagctggacccagcacacttactggtgAtacattc**G**cag
cacatcatagagatagtccttctctgcatcattttcaatcagaaaatccacctggaaaatccaatagcagaatcacagctccaggctcagcaccc

urt2: (sense-orientation):
5´- ggattttccaggtggattttctgattgaaaatgatgcagagaaggactatctctatgatgtgctg**C**gaatgtaccaccagtaagtgtgctg
gCtccagctcttgtgggccacttgggttcctttgtcttcagggagccctgggatgggttgttctgagacagaggagctcagagggtgg

urt1.1: (sense-orientation)
5´-tctcctccctgaggtctgctatgggtgggggtgctgagcctggagctgtgattctgctattggattttccaggtggattttctgattgaaaat
gatgcagagaaggactatctctatgatgtgctg**C**gaatgtaTcaccagtaagtgtgctgggtccagctcttgtgggccacttgggtt

urt1.2: (sense-orientation)
5´- atgggtgggggtgctgagcctggagctgtgattctgctattggattttccaggtggattttctgattgaaaatgatgcagagaaggactatc
tctatgatgtgctg**C**gaatgtaTcaccagtaagtgtgctgggtccagctcttgtgggccacttgggttcctttgtcttcagggagccc

urt1.3: (sense-orientation)
5´- tctgattgaaaatgatgcagagaaggactatctctatgatgtgctg**C**gaatgtaTcaccagtaagtgtgctgggtccagctcttgtgggc
cacttgggttcctttgtcttcagggagccctgggatgggttgttctgagacagaggagctcagagggtggatgctcacggctcctggaaa

urt1.4: (anti-sense-orientation)
5´- aacccaagtggcccacaagagctggacccagcacacttactggtgAtacattc**G**cagcacatcatagagatagtccttctctgcatcatt
ttcaatcagaaaatccacctggaaaatccaatagcagaatcacagctccaggctcagcacccccacccatagcagacctcagggaggaga

urt1.5: (anti-sense-orientation)
5´- tttccaggagccgtgagcatccaccctctgagctcctctgtctcagaacaacccatcccagggctccctgaagacaaaggaacccaagtg
gcccacaagagctggacccagcacacttactggtgAtacattc**G**cagcacatcatagagatagtccttctctgcatcattttcaatcaga

Appendix Table S1: Experimental animals

pig #	decendence	sex	USH1C status	purpose
**5613**	clone of SSCs 1519	fem	**hom** - *R31X / del*	primary cell culture, ABR, tissue sampling 3 weeks
**5614**	clone of SSCs 1519	fem	**hom** - *R31X / del*	ABR, tissue sampling 3 weeks
**10051**	clone of SSCs 1535 or 1494	fem	**hom** - *R31X / del*	obstacle course, breeding
**10053**	clone of SSCs 1535 or 1494	fem	**hom** - *R31X / del*	obstacle course, clinical examination, tissue sampling 12 months
**10054**	clone of SSCs 1535 or 1494	fem	**hom** - *R31X / del*	obstacle course, breeding, AAV therapy 3.5 years
**10056**	clone of SSCs 1535 or 1494	fem	**hom** - *R31X / del*	obstacle course, AAV therapy 2.5 years
**10057**	clone of SSCs 1535 or 1494	fem	**hom** - *R31X / del*	primary cell culture, ABR, tissue sampling tree weeks
**10058**	clone of SSCs 1535 or 1494	fem	**hom** - *R31X / del*	obstacle course, clinical examination, tissue sampling 12 months
**10439**	re-clone of 5613	fem	**hom** - *R31X / del*	breeding, barrier test
**10618**	clone of SSCs 1535 or 1494	fem	**hom** - *R31X / del*	breeding, barrier test
**AM2**	10051 (hom) x K91 (WT)	male	**het** - *R31X / WT*	breeding
**AM3**	10051 (hom) x K91 (WT)	male	**het** - *R31X / WT*	breeding
**AM11**	10051 (hom) x K91 (WT)	fem	**het** - *R31X / WT*	breeding
**AM28**	AM11 (het) x AM3 (het)	male	**het** - *R31X / WT*	ABR
**AM29**	AM11 (het) x AM3 (het)	male	**het** - *R31X / WT*	ABR
**AM30**	AM11 (het) x AM3 (het)	male	*WT/WT*	ABR
**AM31**	AM11 (het) x AM3 (het)	fem	**hom** - *R31X / R31X*	ABR
**AM32**	AM11 (het) x AM3 (het)	fem	**het** - *R31X / WT*	ABR
**AM33**	AM11 (het) x AM3 (het)	fem	**het** - *R31X / WT*	ABR
**AM34**	AM11 (het) x AM3 (het)	fem	*WT/WT*	ABR
**AM35**	AM11 (het) x AM3 (het)	fem	*WT/WT*	ABR
**AM43**	10051 (hom) x AM2 (het)	fem	**hom** - *R31X / del*	nurturing call, ABR
**AM44**	10051 (hom) x AM2 (het)	fem	**hom** - *R31X / del*	nurturing call, ABR
**AM45**	10051 (hom) x AM2 (het)	fem	**het** - *del / WT*	nurturing call, ABR
**AM46**	10051 (hom) x AM2 (het)	male	**het** - *del / WT*	nurturing call, ABR
**AM47**	10051 (hom) x AM2 (het)	male	**het** - *del / WT*	nurturing call, ABR
**AM48**	10051 (hom) x AM2 (het)	male	**het** - *del / WT*	nurturing call, ABR
**AM49**	10051 (hom) x AM2 (het)	male	**hom** - *R31X / del*	nurturing call, ABR
**AM50**	10051 (hom) x AM2 (het)	male	**hom** - *R31X / del*	nurturing call, ABR
**AM51**	10051 (hom) x AM2 (het)	male	**het** - *del / WT*	nurturing call, ABR
**AM52**	10051 (hom) x AM2 (het)	male	**hom** - *R31X / R31X*	nurturing call, ABR
**AM53**	10051 (hom) x AM2 (het)	male	**het** - *R31X / WT*	nurturing call, ABR

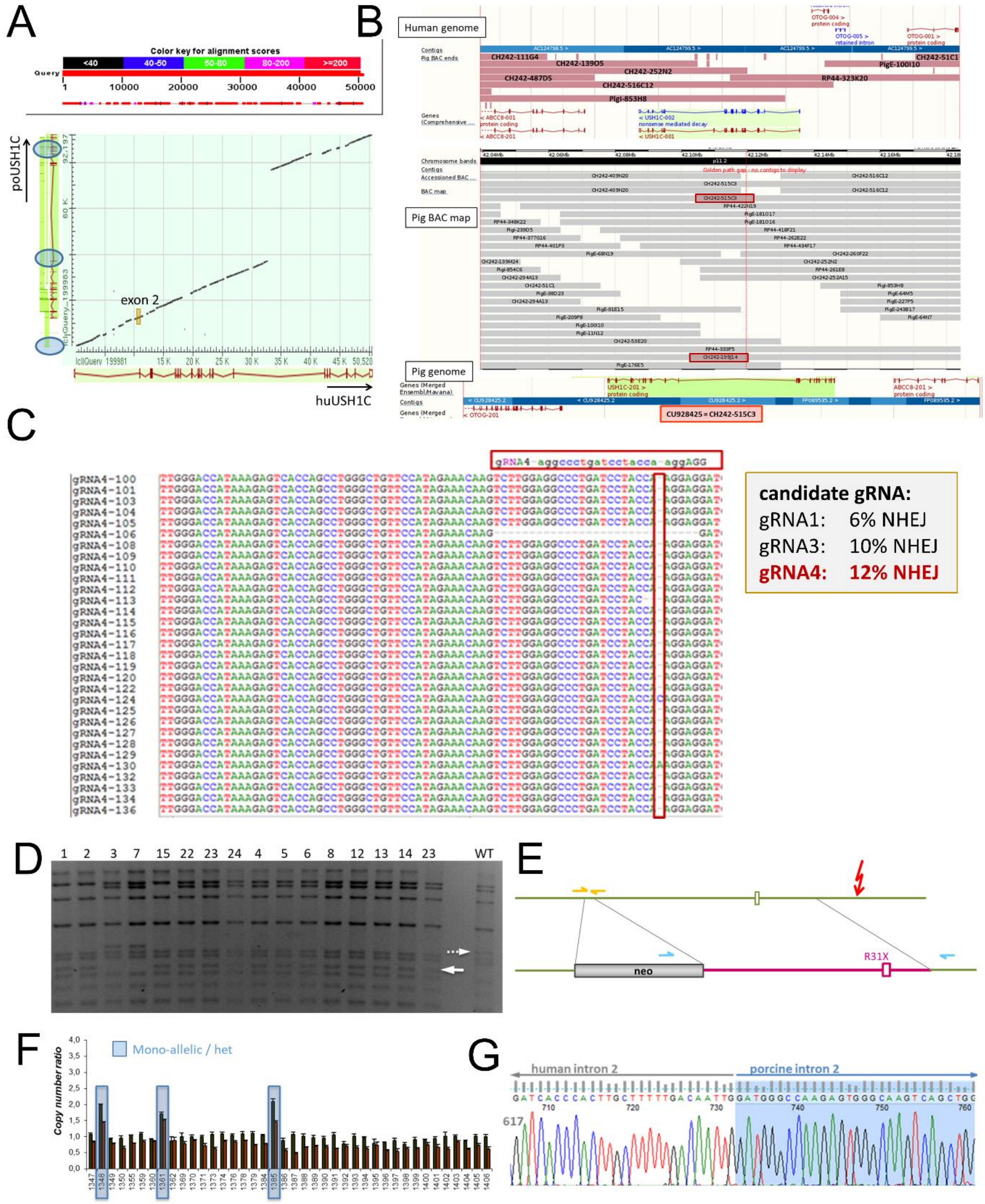

Appendix Figure S1. Modification strategy. (A) The annotation of the porcine *USH1C* gene was grossly confirmed by BLAST search with the b3-splice variant of human USH1C mRNA (GenBank no. NM_153676.4), including exon 2 (yellow box). Exons not correctly defined in the pig genome are marked in blue circles. **(B)** Based on the matches of pig BAC end-sequences to the human genome and the pig reference genome Sscrofa 10.2, the *USH1C* locus was defined in the Pig-Pre BAC map and BAC covering the entire USH1C locus were purchased (red boxes). Clone CH242-515C3 was confirmed by BAC end-sequencing to represent the GenBank no. CU928425. **(C)** Distinct gRNA located within USH1C intron 2 were tested for their ability to introduce NHEJ-based mutations and identified the most potent gRNA4. **(D)** *Spe*I digest of BAC clones confirmed overall integrity of the clone and verified and the appearance of a 6668bp band (arrow) and a 2096bp band (too faint to detect) as well as the disappearance of an 8260bp band (dotted arrow). **(E)** For screening single cell clones (SSCs), primers for the qPCR-based loss-of-wild-type allele approach (yellow arrows) were located opposite of the gRNA cutting site (red lightning). **(F)** In a male cell line, targeting the porcine *USH1C* locus was less efficient than in the female cell line that was used for generating USH1C pigs. **(G)** Sanger sequencing of the modified clones confirmed correct transition from the humanized fragment to the porcine sequence in *USH1C* intron 2.

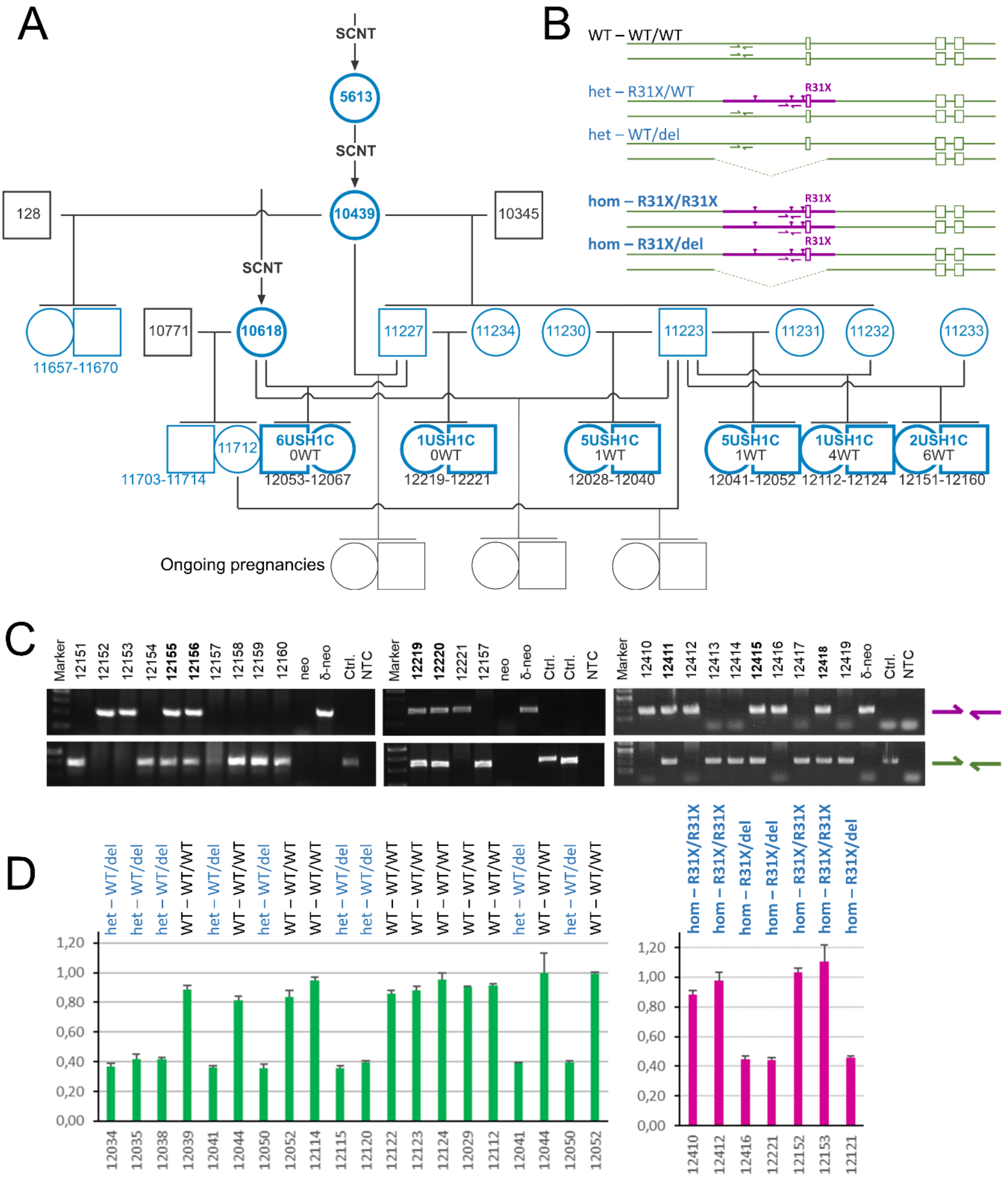

Appendix Figure S2. Breeding of USH1C pigs. (A) Cloned or re-cloned homozygous female USH1C founder animals were mated with unmodified WT boars. Heterozygous F1 animals were either mated with their homozygous ancestors or with heterozygous animals to produce homozygous USH1C pigs in F2. Circles represent female, boxes are male. Black is WT, light blue is heterozygous, dark blue is homozygous USH1C. All founder animals have been verified to have excised the neo cassette (δ-neo) after lipofection with Cre before SCNT. **(B)** Genetic constellations of porcine WT/WT, heterozygous (het) R31X/WT and WT/del and homozygous (hom) R31X/R31X and R31X/del allelic constellations. Positioning of specific primers for the porcine WT (green) and humanized (magenta) alleles are indicated. **(C)** Representative end-point genotyping for porcine WT and humanized alleles in F2 litters. Upper row: R31X fragment (magenta arrows), lower row: WT fragment (green arrows.) Animals that appear positive in both PCR are evidently het R31X/WT. **(D)** Quantitative qPCR-based copy-number detection in F2 litters for the porcine WT alleles in R31X-negative piglets (green) and of humanized alleles in WT-negative piglets (magenta) alleles. Reference qPCR was done with primers binding to the porcine *NANOG* locus. Genotypes confirm segregation according Mendelian rules: 9 WT/del vs 11 WT/WT in non-R31X animals and 4 R31X/R31X vs 3 R31X/del in non WT-animals.

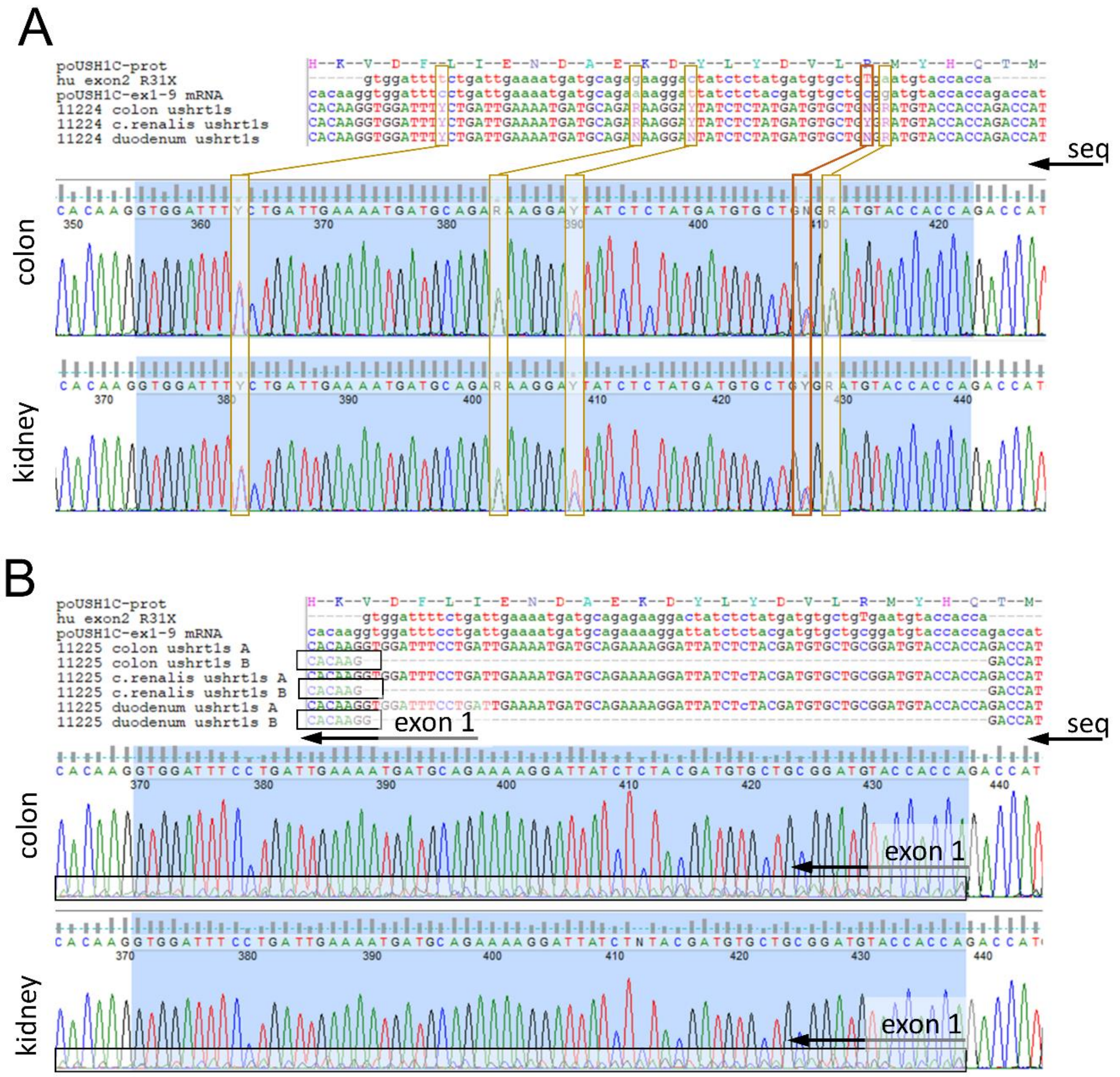

Appendix Figure S3. Transcriptional evaluation of heterozygous F1 animals. RT-PCR spanning porcine USH1C exon1-9 were used to examine transcriptional splicing of exon2. Representative electropherograms were generated by a reverse primer. The resulting electorpherogram was then transformed into the virtual reverse complementary orientation to facilitate comparison to USH1C transcripts in 5´-3´-orientation. **(A)** A het R31X/WT constellation. 4 synonymous nt differences between porcine und human sequence in USH1C exon2 (yellow boxes) and the causative c.C91T polymorphism (orange box) are clearly indicated. The pattern occurred in 2 of 4 sacrificied het animals. **(B)** The preferential abundance of porcine exon2 and a small bystanding sequence correlating to a direct splicing of exon1 to exon3 (black box) indicate a WT/del constellation that appeared in 2 out of 4 animals.

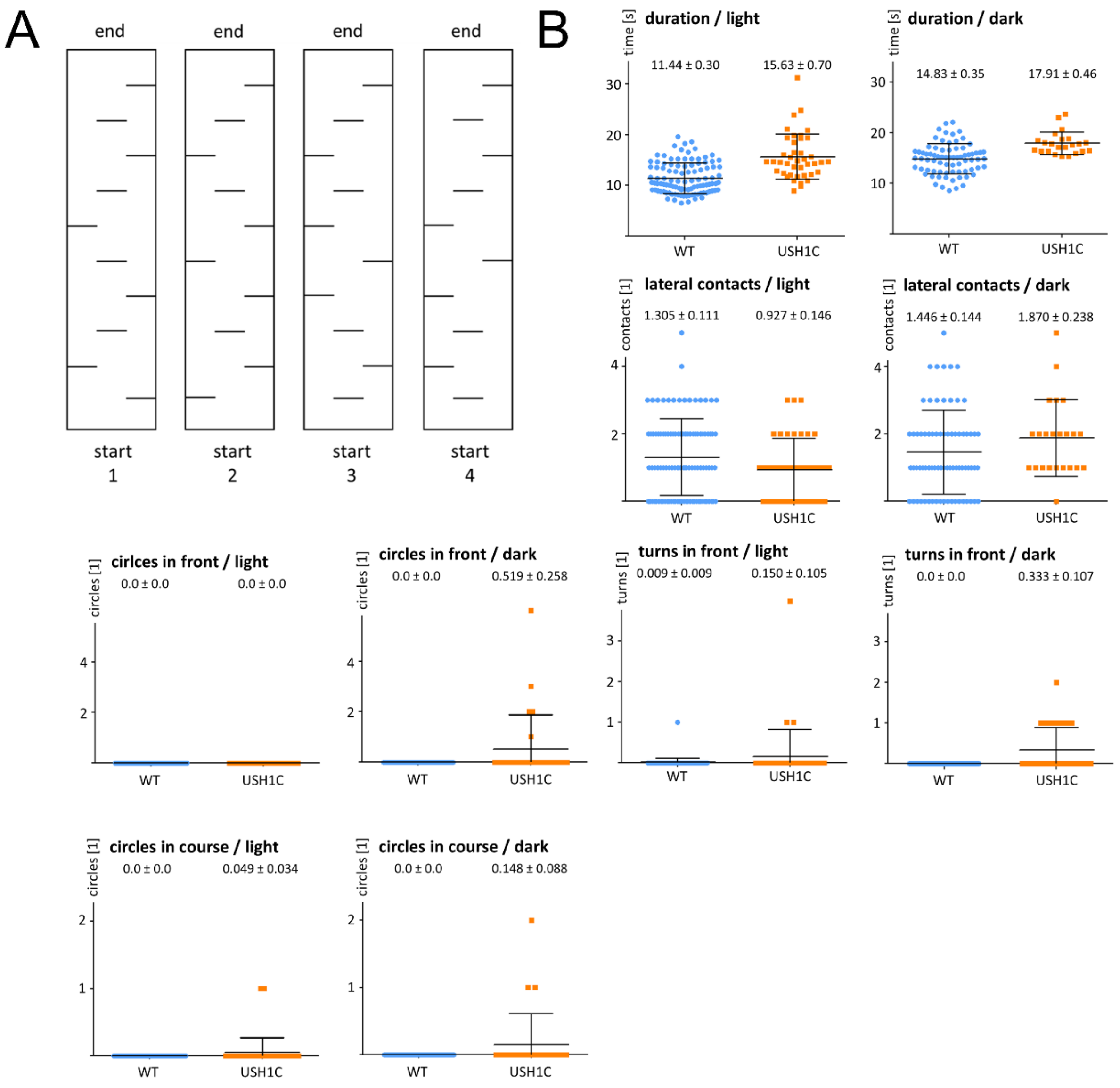

Appendix Figure S4. Extended examination of barrier course (Figure 2A). (A) Shields were placed in consistent distance, but in a different order, either on the left, the right or in the center of the course. **(B)** Indicative parameters were evaluated for USH1C pigs vs WT controls, either in the dark (average of 2,9 lux) or under normal light conditions (average of 136 lux).

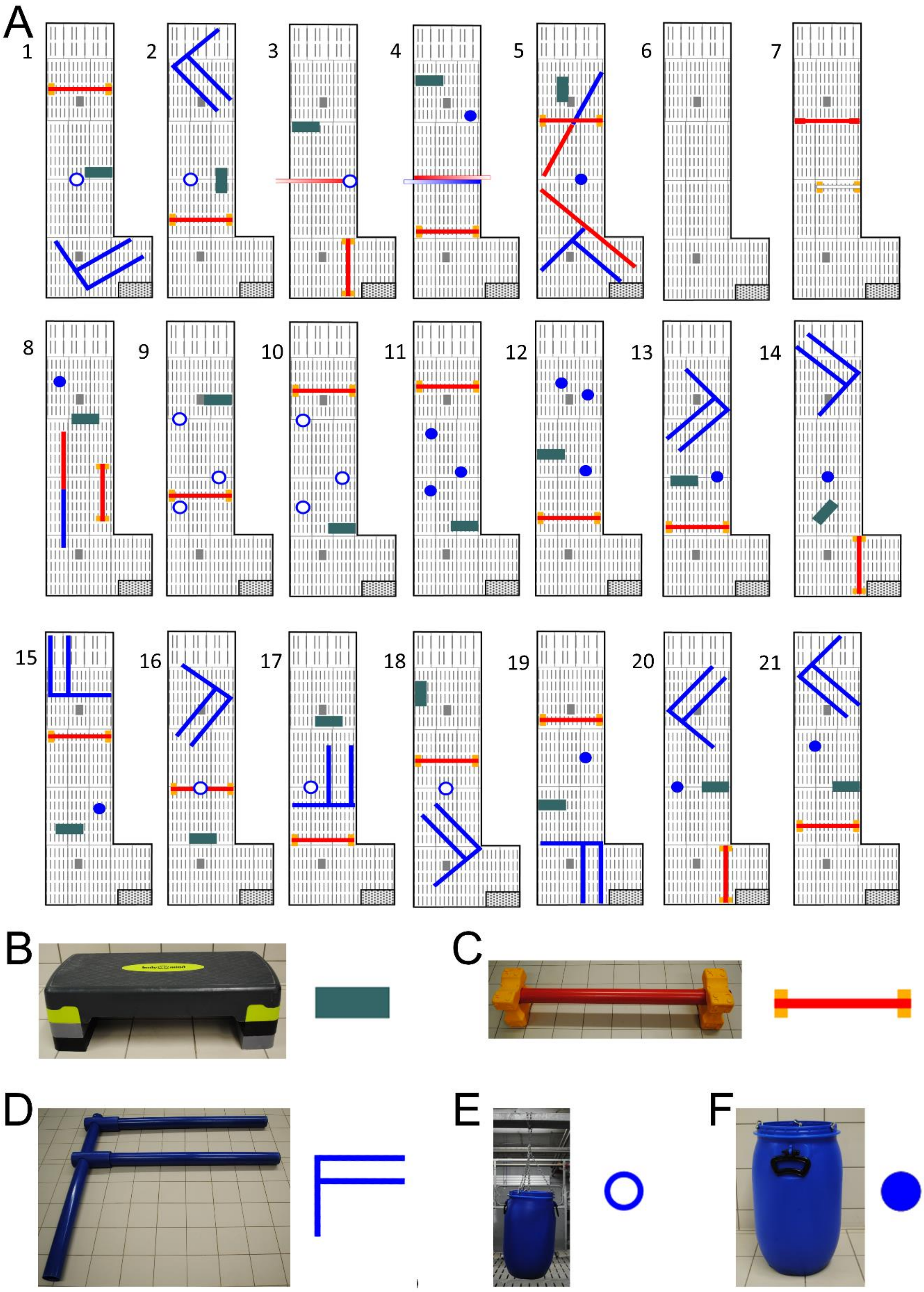

Appendix Figure S5: Set-up of runs in obstacle course (Figure 2B). (A) obstacles were used in different constellation and different order at 28 test days. **(B)** - **(F)** photographs and symbols of most frequently used obstacles: step board **(B)**, cavaletti **(C)**, “F“ formed out of poles **(D)**, barrel hanging from the ceiling **(E)**, barrel placed on the floor **(F)**.

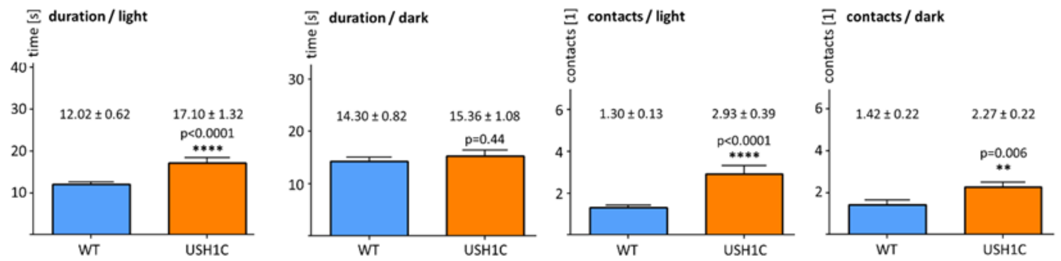

Appendix Figure S6: In-depth statistical analysis of obstacle course (Figure 2B). Data were checked for consistency and normality. Data points are given as mean value ± standard error. Fisher's Exact test or Pearson's test were used to analyze cross tabulations. Generalized linear models with Poission distribution, Median tests, bootstrap-t tests based on 5000 Monte Carlo simulations, t-tests with and without the assumption of homogeneity, Mann-Whitney U tests were used to test continuously distributed variables. All reported tests were two-sided, and p-values < 0.05 were considered statistically significant.

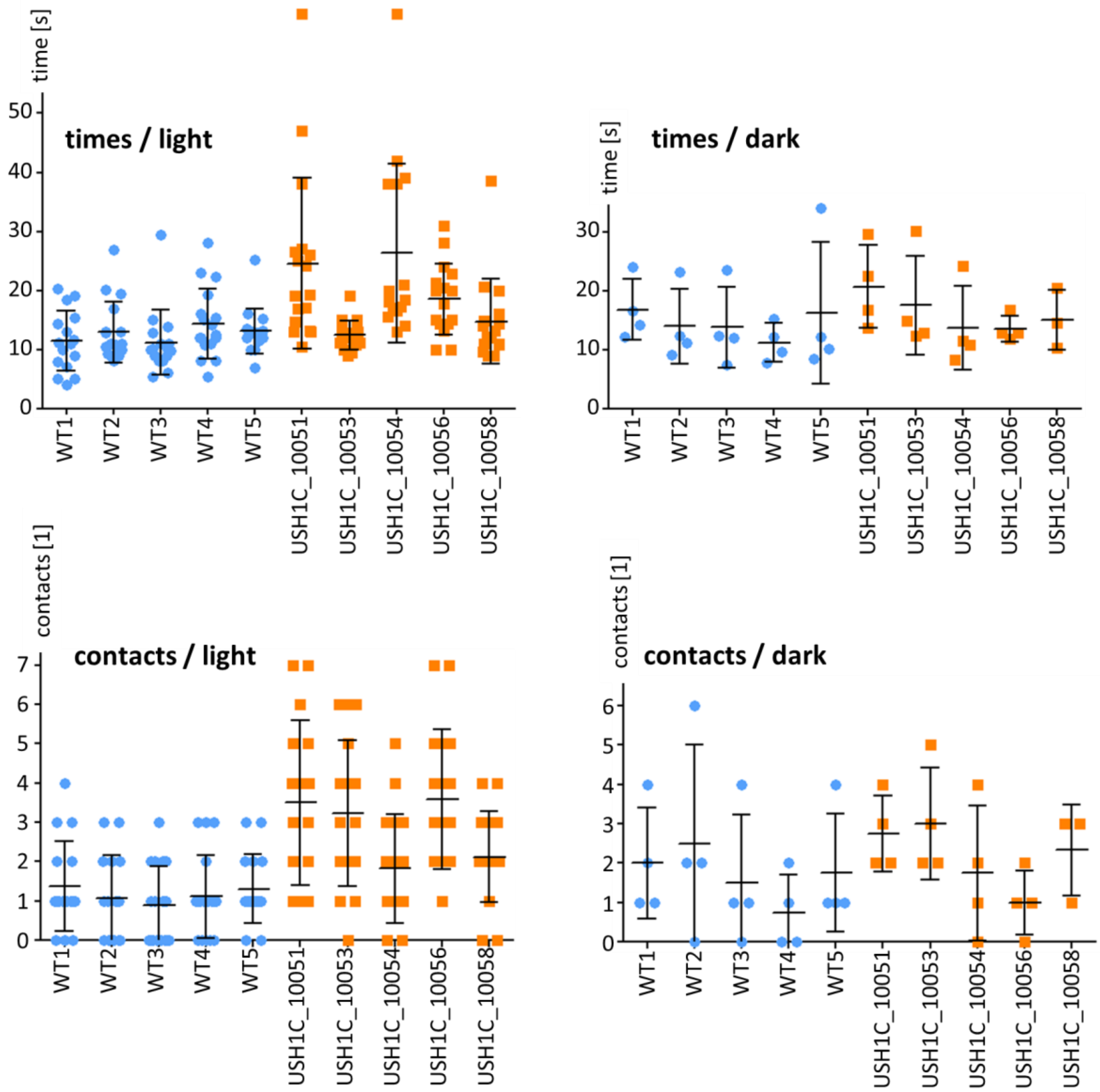

Appendix Figure S7: Individual variability of pigs in obstacle courses. Data from experiments cumulatively shown in Figure 2B were separately examined for the 5 WT and 5 USH1C pigs to estimate the individual variability. Data are given as mean value (MV) ± standard deviation (SD).

Animal	stimulus	right eye							
58USH1C	[cd*s/m2]	a [ms]	b [ms]	a [µV]	b [µV]	a [ms]	b [ms]	a [µV]	b [µV]
DA SF	0.03	20.5	49.9	1.2	20.2	-	-	-	-
	3.0	13.5	34.9	8.9	52.9	-	-	-	-
LA SF	3.0	10.0	25.5	9.9	52.0	-	-	-	-
DA OP	3.0	N2 [ms]	P2 [ms]	OS2 [µV]		-	-	-	-
		15.0	22.9	5.3		-	-	-	-
LA flicker	3.0 (30Hz)	N1 [ms]	P1 [ms]	N1-P1 [µV]		-	-	-	-
		12.9	24.7	44.1		-	-	-	-

Animal	stimulus	right eye							
53USH1C	[cd*s/m2]	a [ms]	b [ms]	a [µV]	b [µV]	a [ms]	b [ms]	a [µV]	b [µV]
DA SF	0.03	18.5	68.4	2.8	33.3	-	-	-	-
	3.0	15.9	35.5	33.9	97.6	-	-	-	-
LA SF	3.0	11.7	26.1	9.8	120.9	-	-	-	-
DA OP	3.0	N2 [ms]	P2 [ms]	OS2 [µV]		-	-	-	-
		15.0	23.2	17.6		-	-	-	-
LA flicker	3.0 (30Hz)	N1 [ms]	P1 [ms]	N1-P1 [µV]		-	-	-	-
		12.3	24.7	113.9		-	-	-	-

Animal	stimulus	right eye							
98_WT	[cd*s/m2]	a [ms]	b [ms]	a [µV]	b [µV]	a [ms]	b [ms]	a [µV]	b [µV]
DA SF	0.03	19.1	41.1	6.7	51.0	-	-	-	-
	3.0	13.2	25.2	78.2	126.7	-	-	-	-
LA SF	3.0	10.9	25.2	16.4	179.5	-	-	-	-
DA OP	3.0	N2 [ms]	P2 [ms]	OS2 [µV]		-	-	-	-
		20.0	22.6	14.2		-	-	-	-
LA flicker	3.0 (30Hz)	N1 [ms]	P1 [ms]	N1-P1 [µV]		-	-	-	-
		11.7	24.7	190.9		-	-	-	-

Animal	stimulus	right eye				left eye			
97_WT	[cd*s/m2]	a [ms]	b [ms]	a [µV]	b [µV]	a [ms]	b [ms]	a [µV]	b [µV]
DA SF	0.03	-	-	-	-	-	-	-	-
	3.0	13.2	26.7	66.3	184.7	13.2	26.7	80.4	181.9
LA SF	3.0	12.9	25.5	23.8	249.8	9.7	25.5	15.6	246.7
DA OP	3.0	N2 [ms]	P2 [ms]	OS2 [µV]		-	-	-	-
		-	-	-	-	-	-	-	-
LA flicker	3.0 (30Hz)	N1 [ms]	P1 [ms]	N1-P1 [µV]		-	-	-	-
		12.3	25.2	243.1		-	-	-	-

Appendix Figure S8: ERG data of USH1C and WT pigs at 1 year of age. Data were collected by RETImap animal as described in the Materials and Methods section on 2 USH1C pigs and 2 age-matched WT control animals.

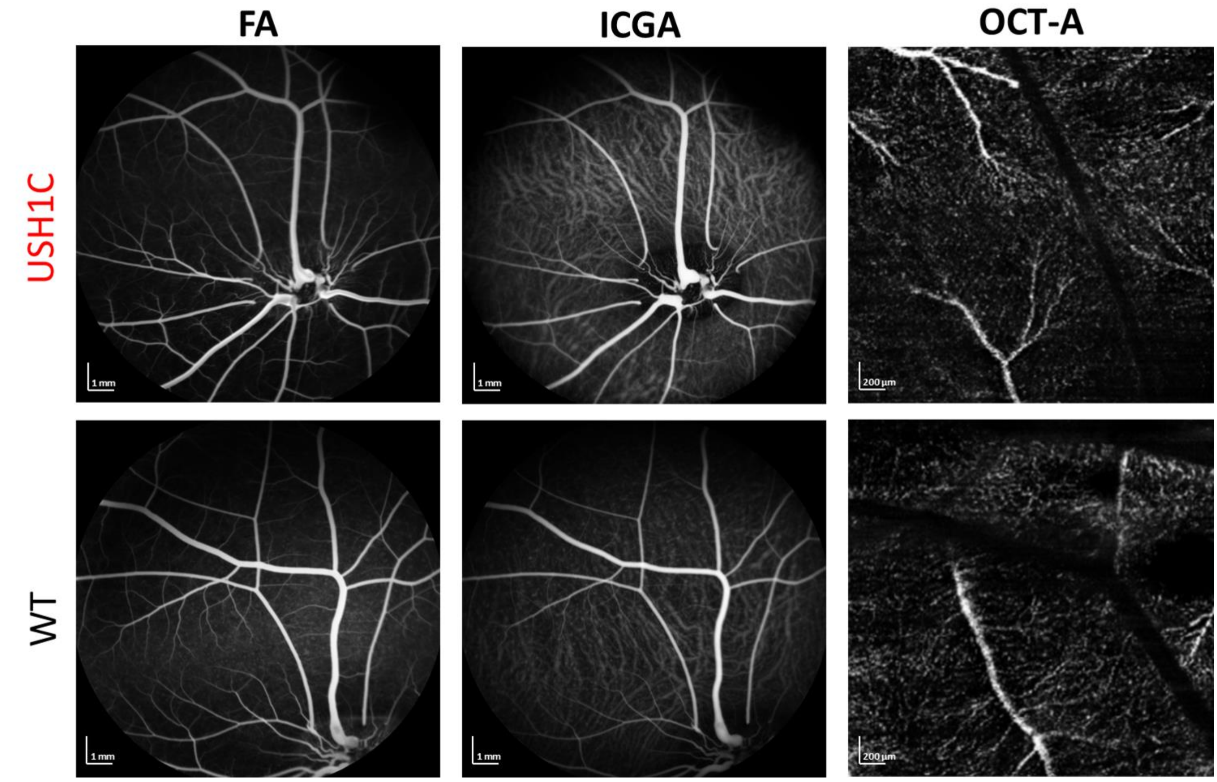

Appendix Figure S9: Intact retinal and choroidal vasculature. *In vivo* retinal imaging in USH1C (top panel) and WT (lower panel) pigs at one year of age using confocal scanning laser ophthalmoscopy and optical coherence tomography (OCT) based angiography demonstrated no qualitative change in retinal or choroidal perfusion. No abnormality at the level of the retinal or choroidal vasculature or associated tissues was evident in fluorescein angiography (FA, left), indocyanine green angiography (ICGA, middle), or high resolution OCT angiography (OCT-A, right).

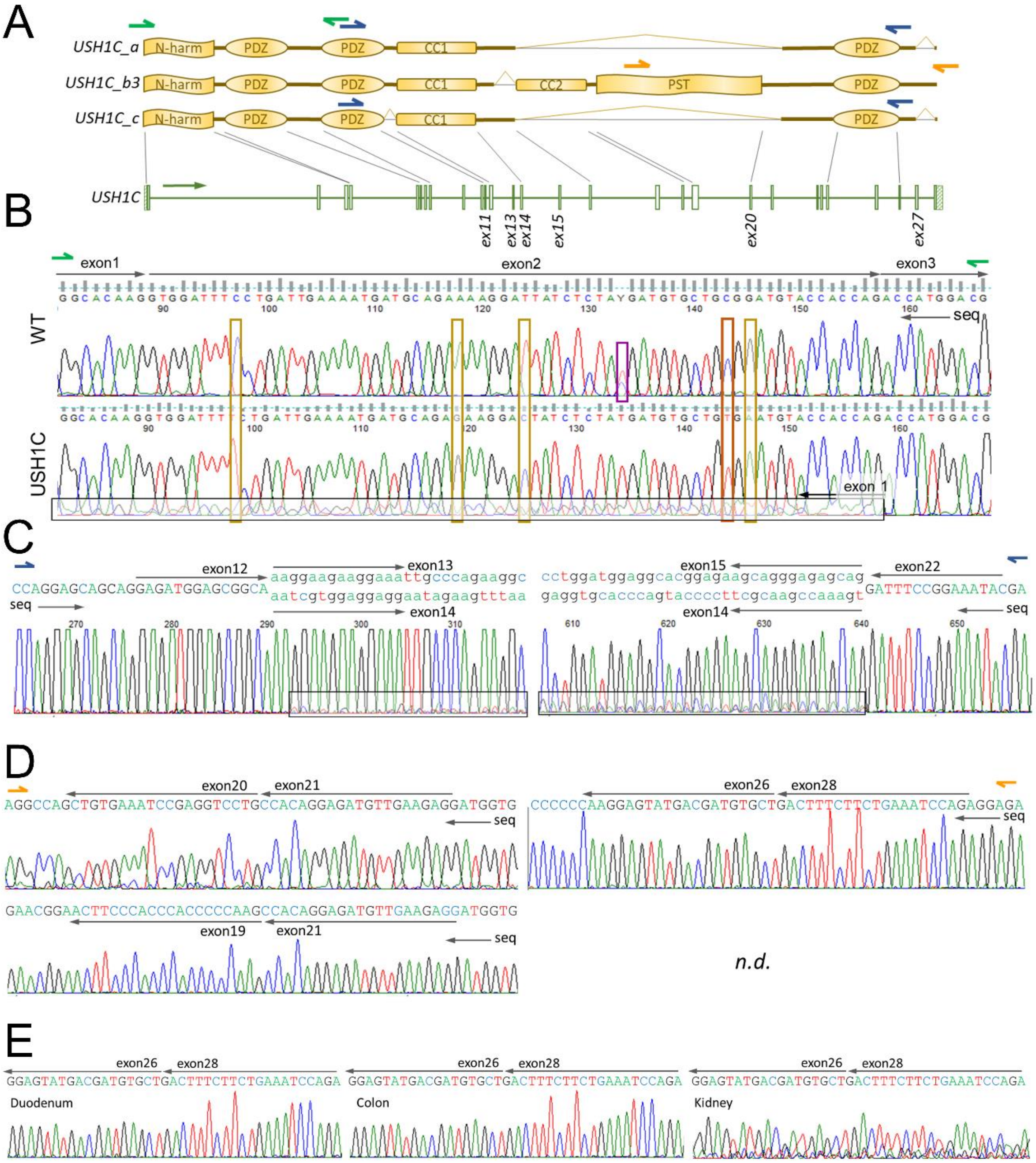

Appendix Figure S10: USH1C splice variants. (A) RT-PCR was used to detect any *USH1C* transcripts (green arrows) and for discriminating *USH1C_a* and *_c* splice variants (blue arrows) from *USH1C_b3* (orange arrows). **(B)** Electropherograms of WT and USH1C pigs show correct splicing of exon 2 into *USH1C* transcripts. Yellow boxes indicate synonymous polymorphisms in exon 2 between porcine and human sequence. The brown box indicated the causative c.C91T / p.R31X mutation. A further synonymous polymorphism between the two alleles of the porcine *USH1C* is enboxed in magenta. The bystanding sequence in the USH1C pig indicates direct splicing of exon 1 to exon 3, confirming the R31X/del allelic constellation of the animal (see Figure S3). **(C)** Amplicons generated with the *USH1C_a / _c* specific RT-PCR (blue arrows) indicate the alternative splicing of exon 13 and exon 15, albeit at low level, in the retina. **(D)** Sequencing of 2 separate RT-PCR fragments amplified by *USH1C_b* specific primers (orange arrows) from retina cDNA indicate alternative splicing of exon 20 (left electropherograms). Exon 27 is lacking in the larger retinal *USH1C_b* fragment (right). **(E)** Duodenum, colon and kidney did also not show evidence of exon 27 in *USH1C_b* transcripts.

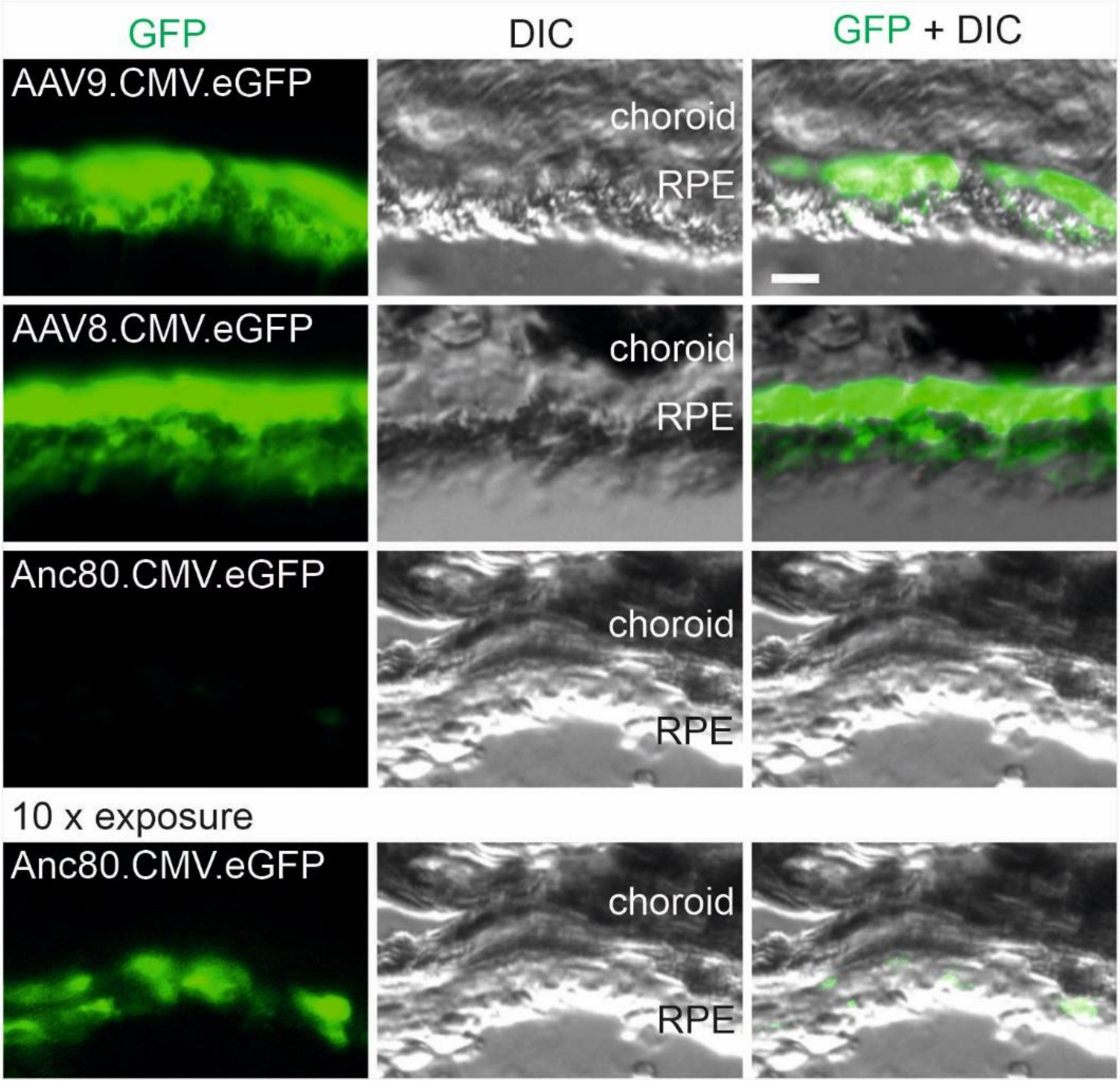

Appendix Figure S11: GFP-expression in cells of the retinal pigment epithelium (RPE) after subretinal injection of AAV8, AAV9 and Anc80 encoding eGFP under the CMV promoter into the eyes of mature WT pigs in vivo. Epifluorescence images of longitudinal sections through the RPE and the attached choroid revealed eGFP expression high GFP expression in RPE cells for AAV8 and AAV9. For Anc80, eGFP expression could be demonstrated only after increased exposure (10 x). Scale bar, 10 µm.

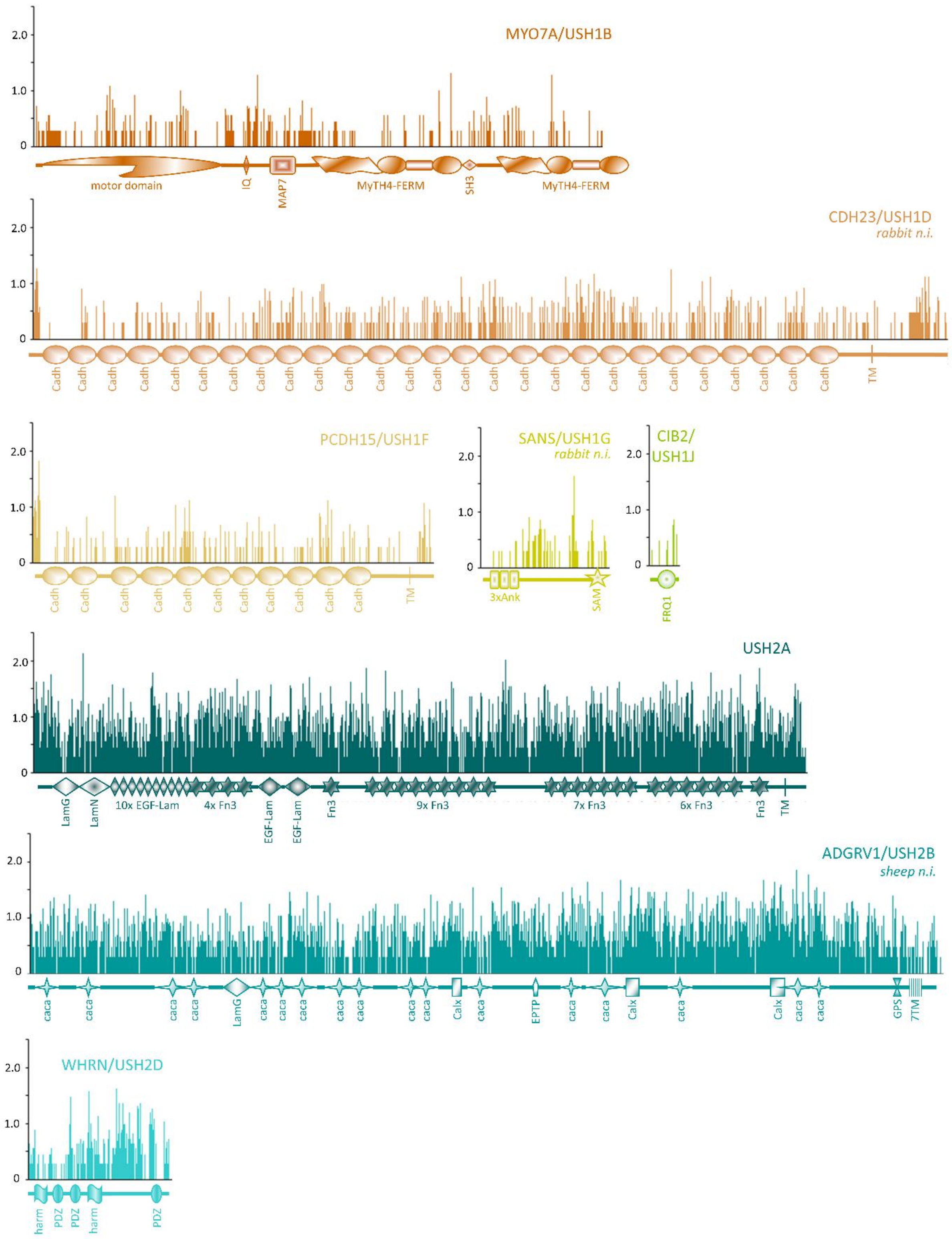

Appendix Figure S12: USH protein conservation. The protein sequences for USH1 and USH2 components from 12 species (human, macaque, marmoset, pig, cattle, sheep, horse, cat, dog, rabbit, mouse, rat) were fetched from the GenBank database, aligned and used for generation of Stewart entropy plots. If not available from the database, specific species were not included (n.i.) for given proteins. The principle structure of protein domains were taken from the respective protein descriptions in the GenBank entry or revealed by the search-for-conserved-domain algorithm (both at https://www.ncbi.nlm.nih.gov/) and indicated below the entropy plot. USH1 components appear substantially more conserved than USH2 components.
