# Supplementary material for: Early disruption of photoreceptor cell architecture and loss of vision in a humanized pig model of usher syndromes
Source: EMBO Mol Med. 2022 Mar 7;14(4):e14817. doi: 10.15252/emmm.202114817 (PMC8988205; doi:10.15252/emmm.202114817)
Supplement: Supplementary file 2 — Expanded View Figures PDF [file EMMM-14-e14817-s011.pdf]

Expanded View Figures

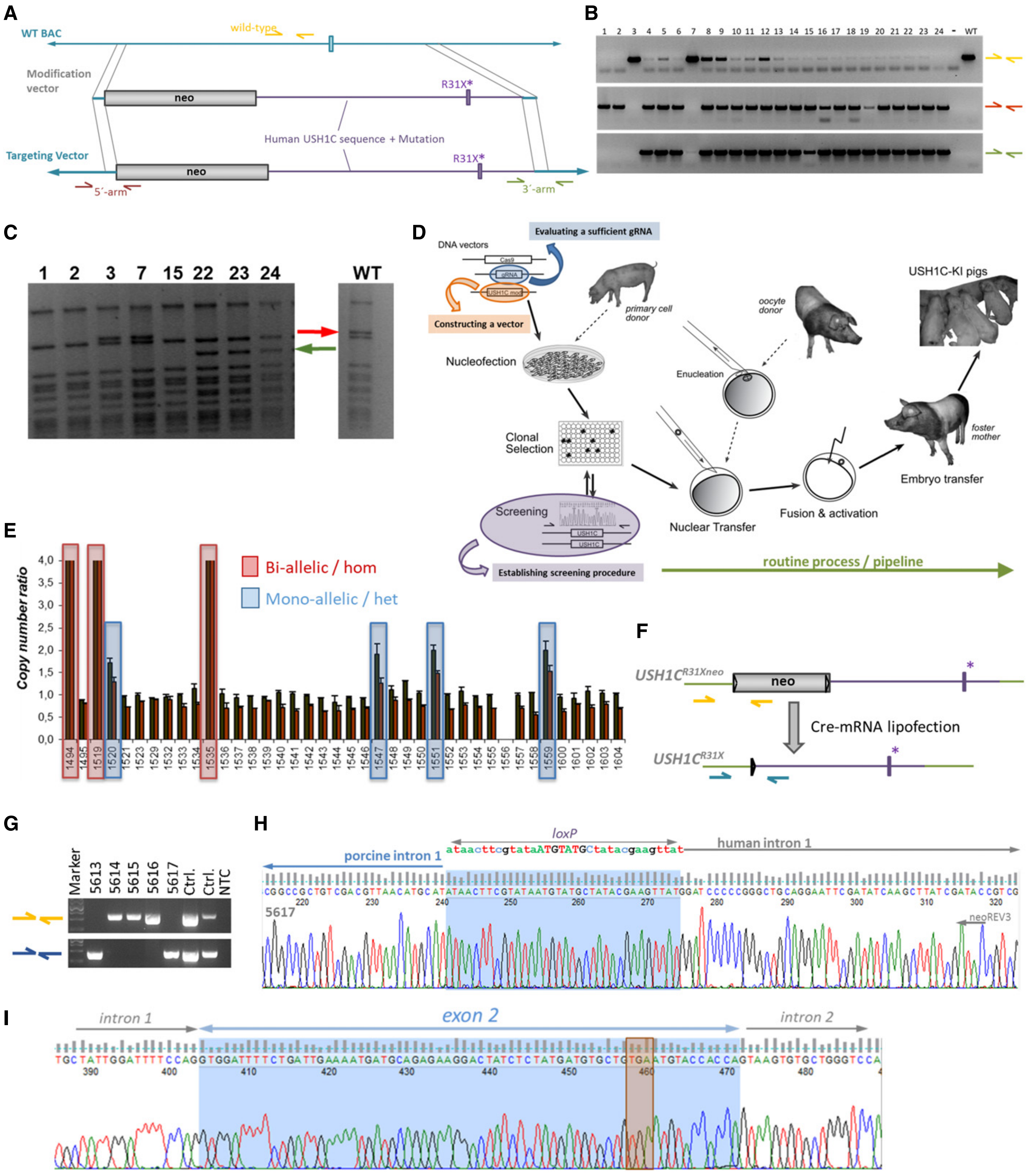

Figure EV1.

© 2022 The Authors EMBO Molecular Medicine 14: e14817 | 2022 25 of 24

◀ **Figure EV1. Modification process.**

A BAC clone CH242-515C3 was modified by bacterial recombineering with a modification vector comprising the intended 1.5 kb human USH^{R31X} segment (magenta) and a floxed *neo* selection cassette. Arrows represent primer for endpoint PCR to screen for correct recombination.

B Correctly modified BAC clones are characterized by negative wild-type PCR (yellow) and positive PCR spanning across the 5′-arm (brown) and 3′-arm (green) of homology.

C *Xba*I digest of BAC clones for confirming integrity of the clone and verified the disappearance of an 18,779bp band (red arrow) and the appearance of a 14,194 bp band (green arrow) and a 5,020 bp band (too faint for detection).

D Modified BAC vectors were co-transfected with plasmids expressing Cas9 and gRNA4 into pig cells. Clonal selection for neomycin-resistance delivered single cell clones (SSCs) which were expanded for screening and subsequent usage in SCNT.

E SSCs were screened for modification by a qPCR-based loss-of-wild-type-allele approach. Inverse copy number ratios of the *USH1C* locus vs reference sites in the *POU5F1* and *NANOG* genes indicated SSCs with bi-allelic (red boxes) and mono-allelic (blue boxes) modification. Each qPCR was run in a duplicate, facilitating the calculation of 4 quotients of reference site : target site copy numbers for each sample. The mean value of these quotients are given ± SD. For ensuring characterization of SSCs, 2 independent reference sites were used and SSCs that appeared modified in the first screening were used in an independent experiment for confirmation.

F Before SCNT, SSCs were lipofected with Cre-encoding mRNA to remove the *neo* selection cassette. Primer sets were designed for discriminating founder animals with (yellow arrows) and without (blue arrows) *neo*.

G Representative genotyping of a founder litter, indicating that animals 5613 and 5617 have sufficiently excised *neo*. For controls (Ctrl.), WT genomic DNA was mixed with modified BAC for the neo-PCR (yellow) and mixed with a modified BAC that had been treated with Cre for the delta-neo PCR (blue).

H Representative electropherogram of PCR sequencing, confirming correct excision of *neo* in 5617. As a consequence of Cre-mediated excision, a single lox-site remains at the junction between porcine and human sequence.

I Sanger sequencing confirmed also abundance of a correctly modified humanized exon 2. The orange box indicates the TGA-nonsense codon causing the R31X mutation.

© 2022 The Authors

© 2022 The Authors

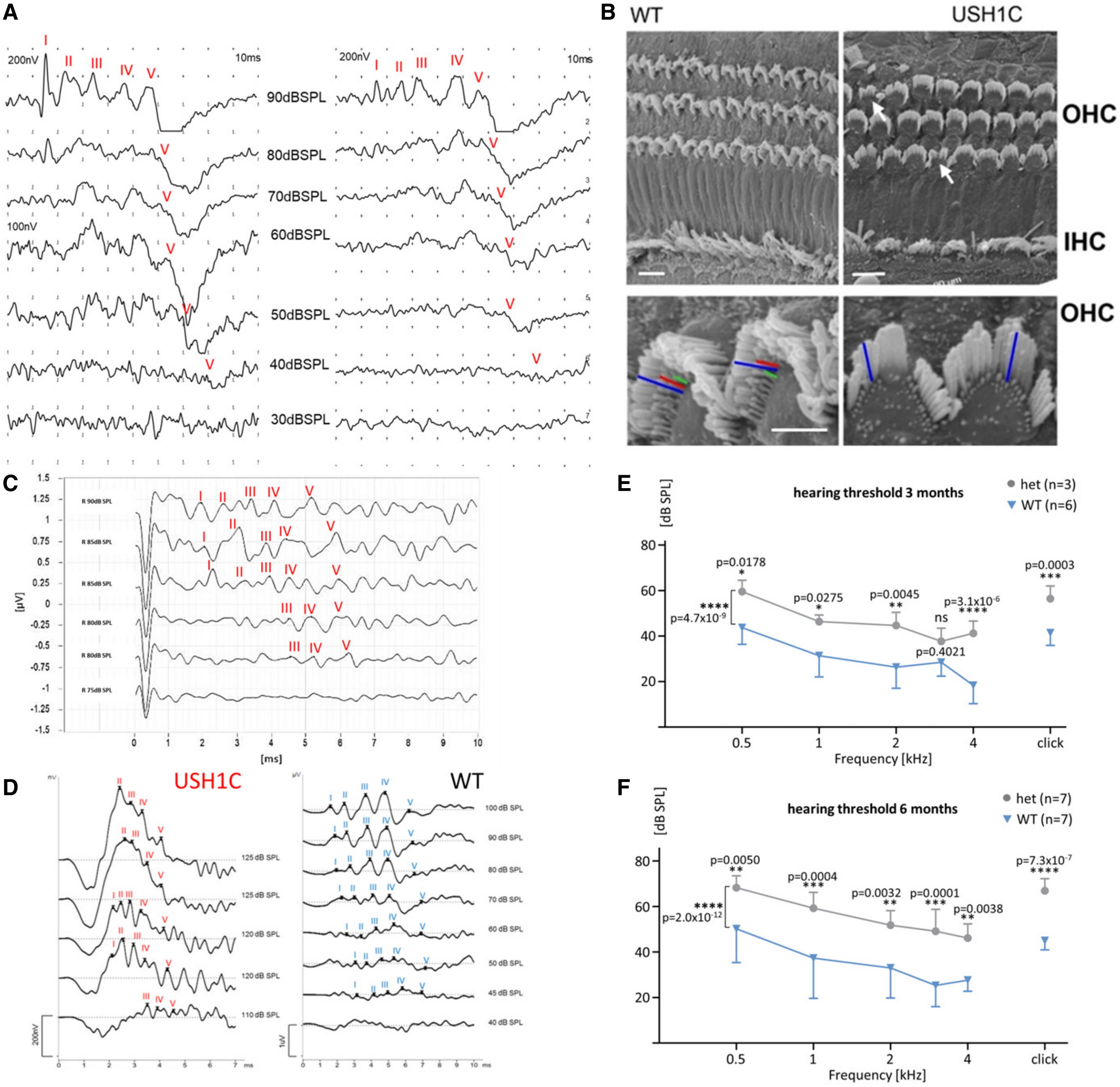

Figure EV2. Impaired auditory system in USH1C pigs.

A ABR measurements were conducted on two WT pigs to determine the threshold for sufficient detection of response peak V to click stimuli.

B Scanning electron microscopy of the cochlear sensory epithelium in 3-week old WT and USH1C pigs reveals alterations in the hair bundle arrangements of outer (OHC) and inner cochlear hair cells (IHC) (arrows). USH1C OHC lack stereocilia rows in the hair cell bundles, indicated by colored lines at higher magnification. Scale bars, upper panel 10 µm; lower panel: 5 µm.

C, D (C) and (D) show exemplary response curves for determining hearing thresholds (dB SPL) by click ABR in an 8-week-old USH1C animal (C) as well as 2-year-old USH1C and WT pigs (D).

E, F Frequency-specific ABR of animals at an age of 3 months (E) and 6 months (F) indicate an increased threshold for heterozygous USH1C$^{+/-}$ pigs, compared to WT littermate controls. Data points are presented as mean value (MV) ± SD. Statistical examination was carried out by *t*-test for each frequency, a 2-side ANOVA test was performed over all frequencies. **P* < 0.05, ***P* < 0.01, ****P* < 0.001, *****P* < 0.0001.

© 2022 The Authors EMBO Molecular Medicine 14: e14817 | 2022 27 of 24

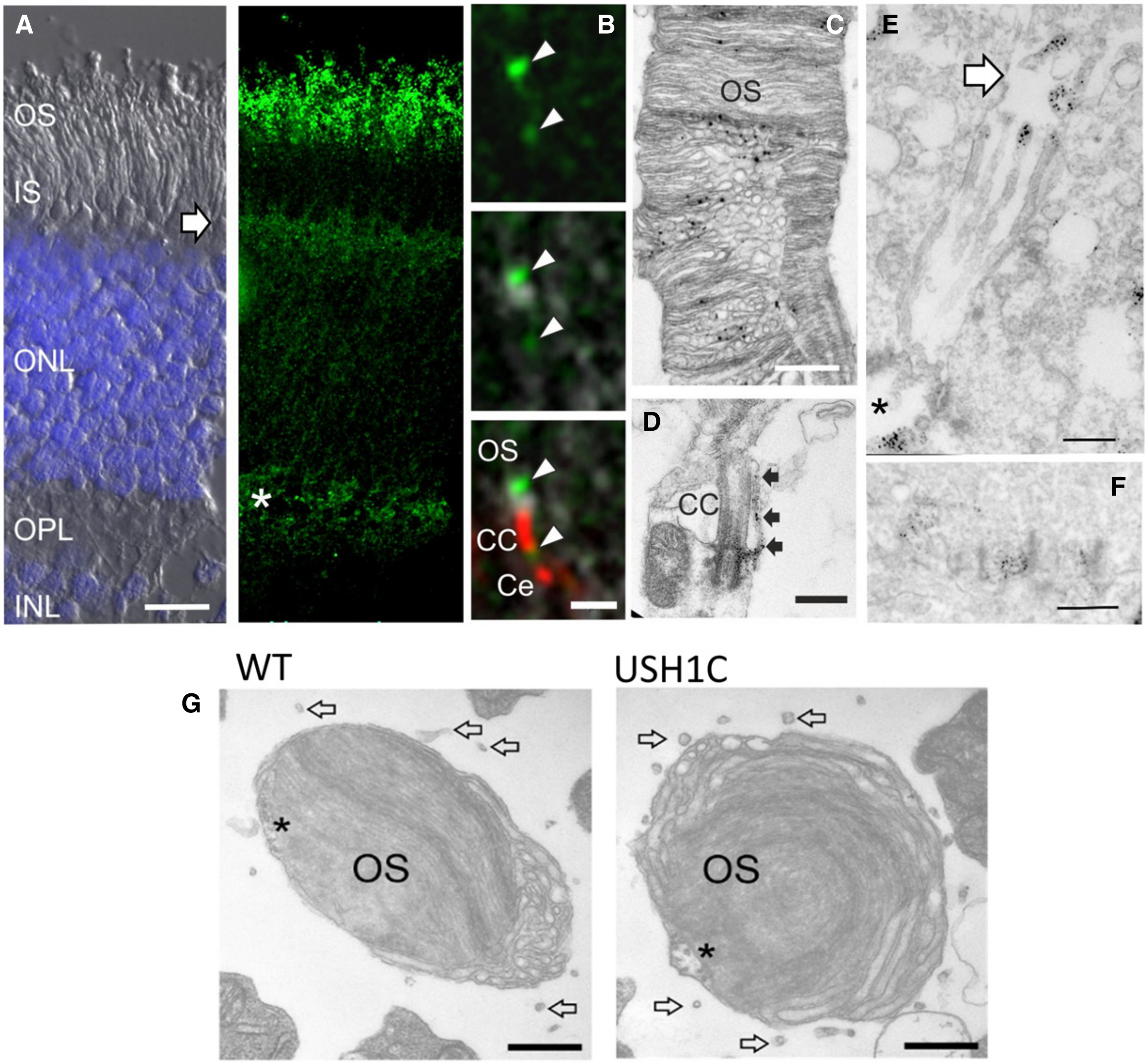

Figure EV3. Localization of harmonin in the WT retina.

Representative staining of longitudinal sections from porcine WT retina (2 pigs, 1 retina each, 4 TR).

A Harmonin (green) is present in the layer of PRC outer segment (OS), the outer limiting membrane (arrow) and in the outer plexiform layer (OPL, asterisk), where PRC synapses are located. Left image: DIC image super-exposed with DAPI for nuclei in outer nuclear layer (ONL) and inner nuclear layer (INL). Right image: Immunohistological staining with anti-harmonin antibody. IS, PRC inner segment. Scale bar 10 μm.

B Immunohistological co-localization of harmonin (green) and the connecting cilia (CC)/centriole (Ce) marker centrin (red) at the OS base (arrowheads, scale bar 1 μm).

C, D (C) Immunoelectron microscopy detects harmonin at PRC OS discs (scale bar 500 nm) as well as (D) at the base of connecting cilia (CC, scale bar 500 nm) and in calyceal processes (arrows).

E, F (E) In Müller glia cells, harmonin is localized at microvilli tips (arrow, scale bar 500 nm), at the cell adhesion region (asterisk) as well as (F) in cone synaptic pedicles (scale bar 500 nm).

G Horizontal cross sections through cone OS reveal persistence of calyceal processes in USH1C pig (arrows, scale bar 400 nm). Asterisks indicate axoneme projections into the OS.

 © 2022 The Authors
 © 2022 The Authors

Figure EV4. CRISPR/Cas-mediated gene repair.

A In an initial approach the 2 oppositely oriented gRNA1 and gRNA2 and repair oligonucleotides urt1 and urt2 were tested for their efficacy to introduce NHEJ-mediated indel formation. Exon 2 is marked by a pink box. The cutting sites of the Cas9 are shown as red arrows and the distinct positions at which the respective oligo-nucleotides should introduce blocking mutations are indicated by magenta boxes. The position of T91 and its corrected variant C91 is indicated by a blue box.

B Sanger sequencing electropherograms were used to estimate efficacy of HDR after co-transfection of plasmids expressing Cas9 and a gRNA and commercially synthesized ssODN repair templates into primary cells from USH1C pigs. PCR products from mixed cell clones were analyzed for NHEJ and HDR by the ICE CRISPR Analysis Tool.

C Optimization was performed with gRNA urt1 and five distinct repair oligo-nucleotides in three independent experiments. The rate of HDR and NHEJ was determined as in (B).

D Single cells clones were generated from the pool nucleofected with gRNA1 and urt1.3 and analyzed by Sanger sequencing of PCR products. A diverse pattern of distinct modifications was observed in 68 examined single cell clones (SSCs). Representative electropherograms from designated SSCs are shown with the frequency of the respective pattern indicated at the right side. The cutting site of Cas9 as well as the correcting and blocking mutation are indicated as in (A). “non mod.” indicates SSCs without changes at the target site. NHEJ appeared either as deletions or insertions. HDR events were mostly restricted to the correct transformation of the correcting and blocking mutations. Some SSCs, however, showed accompanying mutations in the intronic (int SNP) or exonic (ex SNP) regions, with the latter potentially causing amino acid exchanges. Occasionally as well, HDR occurred only at the blocking mutation site, which is located closer to the cutting site than the correcting mutation site.

© 2022 The Authors

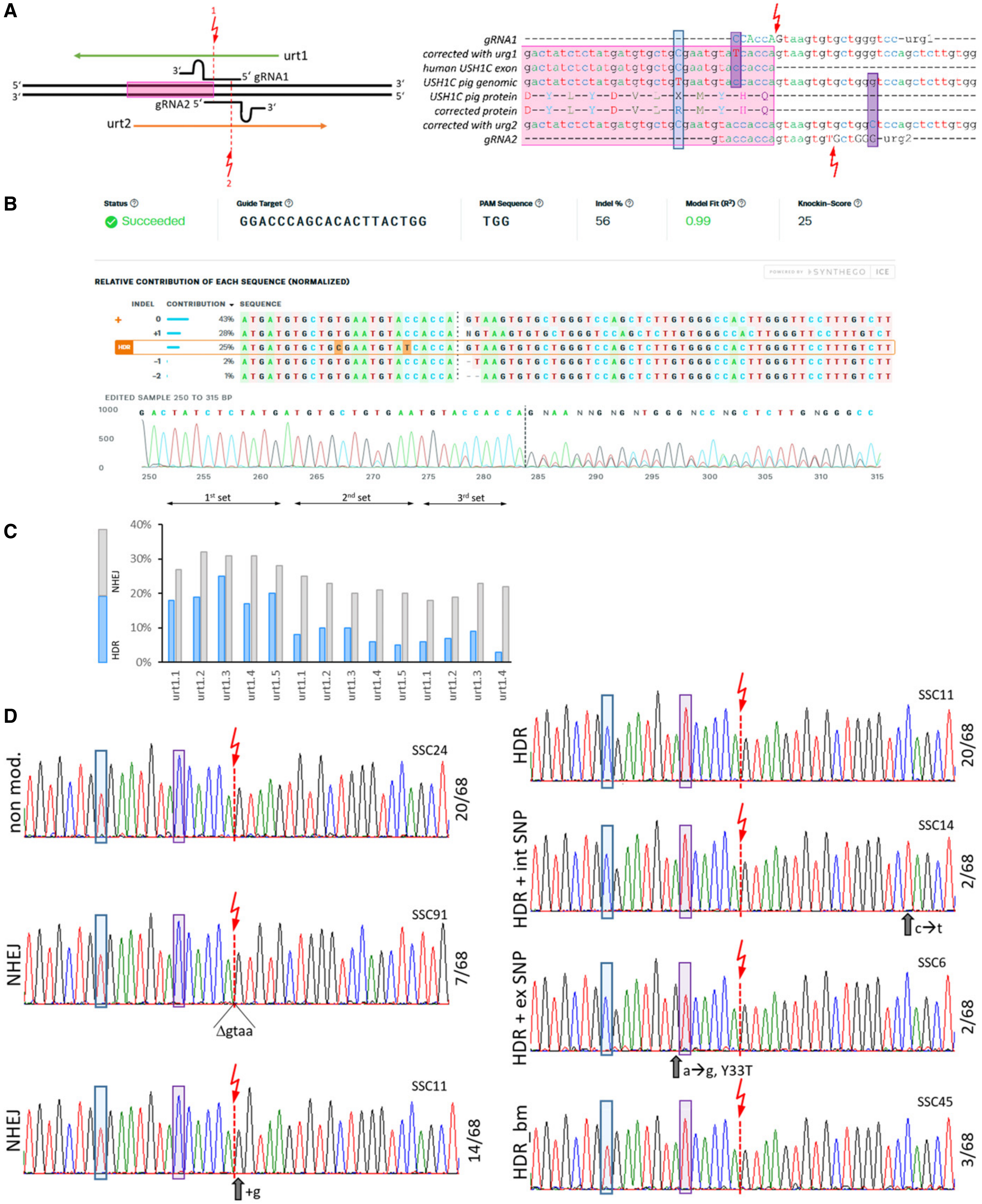

Figure EV4.

 © 2022 The Authors

A

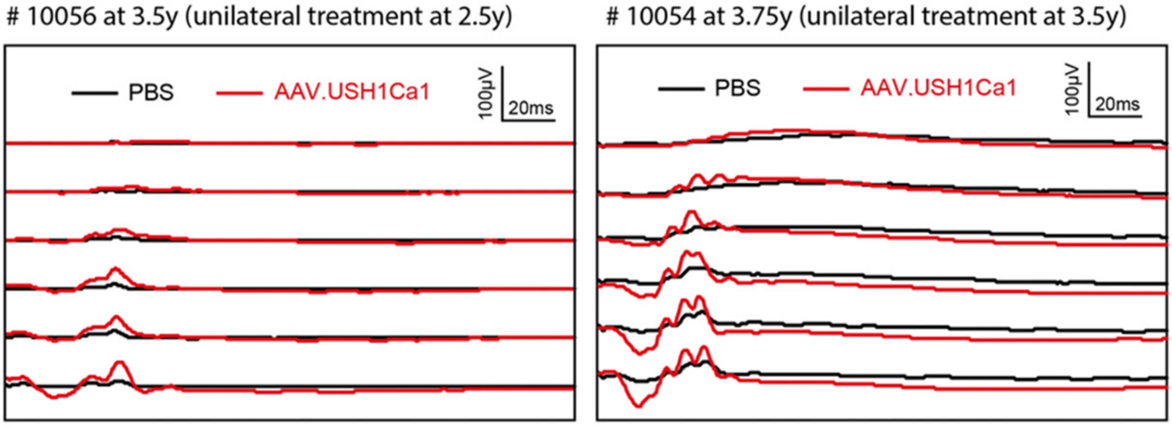

B

Animal	stimulus	left eye, PBS treated				right eye, AAV treated			
10056	[cd*s/m2]	a [ms]	b [ms]	a [µV]	b [µV]	a [ms]	b [ms]	a [µV]	b [µV]
dark adapted single flash intensity series	0.01	26.6	39.1	1.8	10.1	22.7	46.5	0.1	13.0
	0.03	18.0	36.7	0.9	8.3	19.9	44.9	5.8	30.4
	0.1	15.6	38.7	1.5	15.5	18.8	39.8	12.3	60.4
	0.3	16.0	37.9	3.9	28.2	17.2	38.7	29.9	104.0
	1.0	15.6	38.3	6.6	34.2	17.6	38.7	29.7	103.5
	3.0	15.6	39.8	9.1	29.3	16.8	40.2	79.7	150.4
LA SF	3.0	14.8	27.3	2.7	89.8	15.2	27.7	20.1	174.0
LA flicker	3.0 (30Hz)	15.6	27.3	15.2	64.5	15.2	27.7	29.1	123.8

Animal	stimulus	left eye, PBS treated				right eye, AAV treated			
10054	[cd*s/m2]	a [ms]	b [ms]	a [µV]	b [µV]	a [ms]	b [ms]	a [µV]	b [µV]
dark adapted single flash intensity series	0.01	23.4	50.0	9.4	25.7	18.0	47.7	1.7	62.3
	0.03	14.1	45.7	1.4	49.0	17.6	42.6	7.9	94.6
	0.1	19.9	35.2	3.6	57.3	15.6	32.8	20.7	147.5
	0.3	16.8	33.2	3.1	74.6	15.2	31.3	54.2	191.0
	1.0	14.8	36.3	5.1	90.3	14.5	35.5	109.9	244.1
	3.0	14.8	37.9	20.3	89.3	14.1	37.1	144.9	249.1
LA SF	3.0	14.8	28.5	1.6	102.1	12.9	28.1	37.9	297.5
LA flicker	3.0 (30Hz)	14.1	28.1	13.1	74.0	12.9	27.3	56.4	237.5

Figure EV5. Efficacy of AAV gene therapy in USH1C pigs.

2 USH1C pigs underwent sub-retinal injection of an Anc80-capsid expressing harmonin_a1 under control of a CMV promoter in the right eye and of PBS in the left eye. Pig 10056 was injected at an age of 2.5 years (2.5 y) and terminated after 12 months with ERG evaluation and tissue sampling for molecular analysis. Animal 10054 was injected at an age 3.5 y with the same setting and monitored intermittently after 3 months.

A Response to single flashes after dark adaptation in the AAV-treated eye (red) and in the sham-treated eye (black).

B Quantification of response times and amplitudes of a- and b-waves after dark in two treated USH1C pigs. LA SF, light adapted single flash. LA flicker, light adapted flicker.
