# Supplementary material for: Early disruption of photoreceptor cell architecture and loss of vision in a humanized pig model of usher syndromes
Source: EMBO Mol Med. 2022 Mar 7;14(4):e14817. doi: 10.15252/emmm.202114817 (PMC8988205; doi:10.15252/emmm.202114817)
Supplement: Supplementary file 3 — Movie EV1 [file EMMM-14-e14817-s003.zip › EMM-2021-14817-V3-Movie_EV1.docx]

**Movie EV1: post-natal vestibular dysfunction in USH1C piglets.** The movie shows the circling of an USH1C piglet in a rescue deck unit. The movement is consistent with that of USH mouse models suffering a pronounced inner ear defect and indicative for vestibular dysfunction. USH1C pigs were not raised in a common box with their mother to avoid crushing by the sow as consequence of the uncontrolled movement of the piglets.
