# Supplementary material for: Early disruption of photoreceptor cell architecture and loss of vision in a humanized pig model of usher syndromes
Source: EMBO Mol Med. 2022 Mar 7;14(4):e14817. doi: 10.15252/emmm.202114817 (PMC8988205; doi:10.15252/emmm.202114817)
Supplement: Supplementary file 4 — Movie EV2 [file EMMM-14-e14817-s002.zip › EMM-2021-14817-V3-Movie_EV2.docx]

**Movie EV2: Spontaneous nystagmus.** The movie shows uncontrolled and uncommon movement of the eye in juvenile USH1C pigs, presumably caused by an asymmetrical tonic firing of vestibular afferents from the right and left labyrinth.
