# Supplementary material for: Early disruption of photoreceptor cell architecture and loss of vision in a humanized pig model of usher syndromes
Source: EMBO Mol Med. 2022 Mar 7;14(4):e14817. doi: 10.15252/emmm.202114817 (PMC8988205; doi:10.15252/emmm.202114817)
Supplement: Supplementary file 5 — Movie EV3 [file EMMM-14-e14817-s007.zip › EMM-2021-14817-V3-Movie_EV3.docx]

**Movie EV3: movement of a WT pig in a barrier test.** The movie shows movement of a WT pig in a barrier course in which pigs have to move past barrier shields to reach the fodder barn for reward. This representative movie shows a foresighted locomotion and smooth trajectory typical for WT pigs
