# Supplementary material for: Early disruption of photoreceptor cell architecture and loss of vision in a humanized pig model of usher syndromes
Source: EMBO Mol Med. 2022 Mar 7;14(4):e14817. doi: 10.15252/emmm.202114817 (PMC8988205; doi:10.15252/emmm.202114817)
Supplement: Supplementary file 8 — Movie EV6 [file EMMM-14-e14817-s008.zip › EMM-2021-14817-V3-Movie_EV6.docx]

**Movie EV6: movement of an USH1C pig in an obstacle parcour.** The movie shows movement of a WT pig in an obstacle course in which pigs have to step over or move past obstacles to reach the fodder barn for reward. This representative movie shows a slow and disoriented locomotion with generally straight direction towards the finish with abrupt changes of direction in front of a barrier.
