# Supplementary material for: Early disruption of photoreceptor cell architecture and loss of vision in a humanized pig model of usher syndromes
Source: EMBO Mol Med. 2022 Mar 7;14(4):e14817. doi: 10.15252/emmm.202114817 (PMC8988205; doi:10.15252/emmm.202114817)
Supplement: Supplementary file 9 — Movie EV7 [file EMMM-14-e14817-s005.zip › EMM-2021-14817-V3-Movie_EV7.docx]

**Movie EV7: clinical hearing test for USH1C pigs.** The movie shows reaction of piglets in a litter from a homozygous USH1C sow with a heterozygous USH1C boar to the nurturing call of their mother. For the vestibular dysfunction of homozygous USH1C animals, piglets are kept in a separate compartment, linked to the box of the mother sow by a lockable door. Upon opening of the door, heterozygous piglets, recognizable by their straight movement, immediately wake up and run for feeding. Homozygous piglets, recognizable by the instable movement due to vestibular dysfunction, are rarely stimulated by their heterozygous littermates, but mostly continue sleeping until they are stimulated by staff.
