# Supplementary figures and images for: Early disruption of photoreceptor cell architecture and loss of vision in a humanized pig model of usher syndromes
Source: EMBO Mol Med. 2022 Mar 7;14(4):e14817. doi: 10.15252/emmm.202114817 (PMC8988205; doi:10.15252/emmm.202114817)

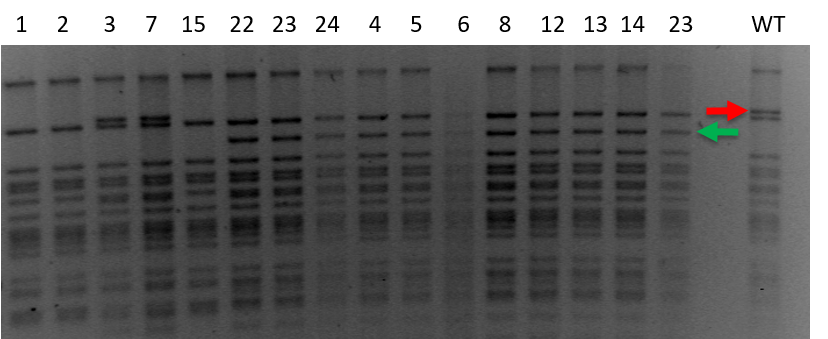

Supplement: Supplementary file 10 — Source Data for Figure 1 [file EMMM-14-e14817-s010.tif]
