# Supplementary material for: Early disruption of photoreceptor cell architecture and loss of vision in a humanized pig model of usher syndromes
Source: EMBO Mol Med. 2022 Mar 7;14(4):e14817. doi: 10.15252/emmm.202114817 (PMC8988205; doi:10.15252/emmm.202114817)
Supplement: Supplementary file 11 [file EMMM-14-e14817-s001.pdf]

EMBO Molecular Medicine

# Early Disruption of Photoreceptor Cell Architecture and Loss of Vision in a Humanized Pig Model of Usher Syndrome

Sophia Grotz, Jessica Schäfer, Kirsten Wunderlich, Zdenka Ellederova, Hannah Auch, Andrea Bähr, Petra Runa-Vochozkova, Janet Fadl, Vanessa Arnold, Taras Ardan, Miroslav Veith, Gianluca Santamaria, Georg Dhom, Wolfgang Hitzl, Barbara Kessler, Christian Eckardt, Joshua Klein, Anna Brymova, Joshua Linnert, Mayuko Kurome, Valeri Zakhartchenko, Andrea Fischer, Andreas Blutke, Anna Döring, Stepanka Suchankova, Jiri Popelar, Eduardo Rodriguez-Bocanegra, Julia Dlugaiczyk, Hans Straka, Helen May-Simera, Weiwei Wang, Karl-Ludwig Laugwitz, Luk Vandenberghe, Eckhard Wolf, Kerstin Nagel-Wolfrum, Tobias Peters, Jan Motlik, M Fischer, Uwe Wolfrum, and Nikolai Klymiuk
**DOI: 10.15252/emmm.202114817**

*Corresponding authors: Nikolai Klymiuk (n.klymiuk@tum.de) , Uwe Wolfrum (wolfrum@uni-mainz.de)*

---

## Review Timeline:

| | |
|---|---|
| Submission Date: | 9th Jul 21 |
| Editorial Decision: | 4th Aug 21 |
| Author Correspondence: | 9th Aug 21 |
| Editor Correspondence: | 12th Aug 21 |
| Revision Received: | 26th Nov 21 |
| Editorial Decision: | 16th Dec 21 |
| Revision Received: | 13th Jan 22 |
| Editorial Decision: | 25th Jan 22 |
| Revision Received: | 4th Feb 22 |
| Accepted: | 9th Feb 22 |

---

*Editor: Lise Roth*

## Transaction Report:

(Note: With the exception of the correction of typographical or spelling errors that could be a source of ambiguity, letters and reports are not edited. Depending on transfer agreements, referee reports obtained elsewhere may or may not be included in this compilation. Referee reports are anonymous unless the Referee chooses to sign their reports.)

4th Aug 2021

Dear Dr. Klymiuk,

Thank you for submitting your work to EMBO Molecular Medicine. We have now heard back from the referees who agreed to evaluate your manuscript. As you will see below, the reviewers raise substantial concerns on your work, which unfortunately preclude its publication in EMBO Molecular Medicine in its current form.

The reviewers acknowledge the interest of the model established in the study, but they also raise a number of issues, pertaining mostly to the lack of information and lack of care in assembling the manuscript.
Another major point relates to the therapeutic angle, which has not been investigated in sufficient depth. While referee #2 initially suggested keeping these data out, the referees then agreed (as we do) that they add translational value to the manuscript, and we would therefore encourage you to strengthen this part.

If you feel you can satisfactorily address these points and those listed by the referees, you may wish to submit a revised version of your manuscript. Please attach a covering letter giving details of the way in which you have handled each of the points raised by the referees. A revised manuscript will once again be subject to review and we cannot guarantee at this stage that the eventual outcome will be favorable.

Revised manuscripts should be submitted within three months of a request for revision; they will otherwise be treated as new submissions, except under exceptional circumstances in which a short extension is obtained from the editor.

When submitting your revised manuscript, please carefully review the instructions that follow below. We perform an initial quality control of all revised manuscripts before re-review; failure to include requested items will delay the evaluation of your revision.

We require:

1) A .docx formatted version of the manuscript text (including legends for main figures, EV figures and tables). Please make sure that the changes are highlighted to be clearly visible.

2) Individual production quality figure files as .eps, .tif, .jpg (one file per figure). For guidance, download the 'Figure Guide PDF' (https://www.embopress.org/page/journal/17574684/authorguide#figureformat).

3) A .docx formatted letter INCLUDING the reviewers' reports and your detailed point-by-point responses to their comments. As part of the EMBO Press transparent editorial process, the point-by-point response is part of the Review Process File (RPF), which will be published alongside your paper.

4) A complete author checklist, which you can download from our author guidelines (https://www.embopress.org/page/journal/17574684/authorguide#submissionofrevisions). Please insert information in the checklist that is also reflected in the manuscript. The completed author checklist will also be part of the RPF.

5) It is mandatory to include a 'Data Availability' section after the Materials and Methods. Before submitting your revision, primary datasets produced in this study need to be deposited in an appropriate public database, and the accession numbers and database listed under 'Data Availability'. Please remember to provide a reviewer password if the datasets are not yet public (see https://www.embopress.org/page/journal/17574684/authorguide#dataavailability).

In case you have no data that requires deposition in a public database, please state so in this section. Note that the Data Availability Section is restricted to new primary data that are part of this study.

6) For data quantification: please specify the name of the statistical test used to generate error bars and P values, the number (n) of independent experiments (specify technical or biological replicates) underlying each data point and the test used to calculate p-values in each figure legend. The figure legends should contain a basic description of n, P and the test applied. Graphs must include a description of the bars and the error bars (s.d., s.e.m.).

7) We would also encourage you to include the source data for figure panels that show essential data. Numerical data should be provided as individual .xls or .csv files (including a tab describing the data). For blots or microscopy, uncropped images should be submitted (using a zip archive if multiple images need to be supplied for one panel). Additional information on source data and instruction on how to label the files are available at
.

8) Our journal encourages inclusion of *data citations in the reference list* to directly cite datasets that were re-used and

obtained from public databases. Data citations in the article text are distinct from normal bibliographical citations and should directly link to the database records from which the data can be accessed. In the main text, data citations are formatted as follows: "Data ref: Smith et al, 2001" or "Data ref: NCBI Sequence Read Archive PRJNA342805, 2017". In the Reference list, data citations must be labeled with "[DATASET]". A data reference must provide the database name, accession number/identifiers and a resolvable link to the landing page from which the data can be accessed at the end of the reference. Further instructions are available at .

9) We replaced Supplementary Information with Expanded View (EV) Figures and Tables that are collapsible/expandable online. A maximum of 5 EV Figures can be typeset. EV Figures should be cited as 'Figure EV1, Figure EV2" etc... in the text and their respective legends should be included in the main text after the legends of regular figures.

- For the figures that you do NOT wish to display as Expanded View figures, they should be bundled together with their legends in a single PDF file called *Appendix*, which should start with a short Table of Content. Appendix figures should be referred to in the main text as: "Appendix Figure S1, Appendix Figure S2" etc.

- Additional Tables/Datasets should be labeled and referred to as Table EV1, Dataset EV1, etc. Legends have to be provided in a separate tab in case of .xls files. Alternatively, the legend can be supplied as a separate text file (README) and zipped together with the Table/Dataset file.

See detailed instructions here:
.

10) For more information: There is space at the end of each article to list relevant web links for further consultation by our readers. Could you identify some relevant ones and provide such information as well? Some examples are patient associations, relevant databases, OMIM/proteins/genes links, author's websites, etc...

11) Every published paper now includes a 'Synopsis' to further enhance discoverability. Synopses are displayed on the journal webpage and are freely accessible to all readers. They include a short stand first (maximum of 300 characters, including space) as well as 2-5 one-sentences bullet points that summarizes the paper. Please write the bullet points to summarize the key NEW findings. They should be designed to be complementary to the abstract - i.e. not repeat the same text. We encourage inclusion of key acronyms and quantitative information (maximum of 30 words / bullet point). Please use the passive voice. Please attach these in a separate file or send them by email, we will incorporate them accordingly.

Please also suggest a striking image or visual abstract to illustrate your article as a PNG file 550 px wide x 300-600 px high.

12) As part of the EMBO Publications transparent editorial process initiative (see our Editorial at http://embomolmed.embopress.org/content/2/9/329), EMBO Molecular Medicine will publish online a Review Process File (RPF) to accompany accepted manuscripts.
In the event of acceptance, this file will be published in conjunction with your paper and will include the anonymous referee reports, your point-by-point response and all pertinent correspondence relating to the manuscript. Let us know whether you agree with the publication of the RPF and as here, if you want to remove or not any figures from it prior to publication.
Please note that the Authors checklist will be published at the end of the RPF.

EMBO Molecular Medicine has a "scooping protection" policy, whereby similar findings that are published by others during review or revision are not a criterion for rejection. Should you decide to submit a revised version, I do ask that you get in touch after three months if you have not completed it, to update us on the status.

I look forward to seeing a revised form of your manuscript as soon as possible. Please use this link to login to the manuscript system and submit your revision: in ot A i e

Yours sincerely,

Lise Roth

Lise Roth, Ph.D
Editor
EMBO Molecular Medicine

***** Reviewer's comments *****

Referee #1 (Remarks for Author):

In this paper the authors describe the generation of a pig model of USH1C through insertion of a human mutation in the pig USH1C gene which encodes for harmonin. Mouse models of Usher syndrome which phenocopy the retinitis pigmentosa observed in patients are lacking, presumably due to the absence of calyceal processes in mice that are present in pigs. Therefore, such large animal model responds to an important unmet need which limits in particular the development of novel therapies. The authors performed a detailed characterization of the phenotype developed by USH1C pigs and, interestingly, they observed both retinal and hearing defects, some of them already present at young ages. Although no evident loss of photoreceptor cells has been observed, rearrangements of the OS structure and increased length of the connecting cilium, in line with the proposed retinal function of harmonin, have been identified. The characterization the authors have performed is very comprehensive and the data is sound.
The manuscript could be improved by taking into accounts the comments below:
- The manuscript suffers from lack of information on the actual number of eyes analyzed. Please clarify in the legend to the Figures 3, 4 and 5 the number of pigs/photoreceptor analysed for each panel. Similarly, in page 4 the authors state "we occasionally observed nystagmus"; please clarify if occasionally refers to the number of animals or to the timing of the observations. Lastly, from the legend to Fig.2 it appears that each dot in the graph represents a run; if this is correct, please clarify from how many animals these runs have been obtained.
- Fig 3B-D. ERG data from all the analysed pigs should be shown to better understand the variability of the phenotype. Accordingly, data reported in the text in page 6 (Sentence: "USH1C animals showed > 70% reduction... and a 60% reduction in the respective b-wave amplitude (86{plus minus}49 µV in USH1C vs. 215{plus minus}50 µV in WT animals")) should be shown in a dot plot graph where data from each animal can be separately appreciated.
- Fig.EV7B-C the differences are significant yet very subtle, is this biologically relevant?
- Although the characterization of the animal model is very comprehensive and solid, potential therapeutic approaches have been evaluated only ex vivo. Further in vivo data would definitively strengthen the paper. Along a similar line please comment why genome editing has been selected rather than AAV-mediate delivery of wild-type harmonin.

Additional minor criticism:
- Supplementary figures are sometimes erroneously referred to as "Fig. S" instead of "Fig. EV"

Referee #2 (Remarks for Author):

The manuscript "Early disruption of photoreceptor cell architecture and loss of vision in a humanized pig model of Usher syndrome" from Grotz et al presents results of the retina and inner ear phenotype in the USH1C pig model. Usher syndrome (USH) is a major cause of deaf-blindness in humans, affecting ~400,000 patients worldwide. Three clinical subtypes, USH1-3, have been defined, with 10 USH genes identified so far, with USH1 being the most severe. Usher type 1 patients can benefit from cochlear implants, however, there are currently no biological treatments available for visual dysfunction. A good animal model for testing retinal therapy would be of value.

In this study, the authors introduced a human mutation into the harmonin-encoding USH1C gene in pigs and generated the first translational animal model for USH type 1 which potentially mimics the full syndromic phenotype in human USH1 patients (characterized by profound hearing loss from birth, vestibular dysfunction, and retinitis pigmentosa). The authors investigated the retinal phenotype of the transgenic USH1CR31X pig analyzing the disease progression in relation to the morphology, the molecular changes and the retinal function.

Mayor comments:

1. The transgenic USH1C pig that has been generated is a unique animal model that potentially develops a phenotype similar to that in patients and is beneficial for examining gene therapy treatments of USH1C. The amount of generated data are impressive. However, the manuscript is not well written, and some results are not clearly presented and not convincing, or not fully described. I would recommend authors rewrite the manuscript and put extra effort into organizing data and making clear figures.

2. The manuscript lacks an overview of the most relevant research that has been conducted in the inner ear and retina. I recommend giving a more thorough intro to the USH1C gene and harmonin (size, localization, and function, known splice forms, expression profile during the development in the inner ear and retina, mouse models if any); the R31X patient-specific mutation, patient visual phenotype and etc. Support the background information with relevant references.
3. Page 3, "A patient-specific disease-causing segment in USH1C leads to Usher syndrome in pigs". The title does not represent the information described in the paragraph. I would change it since the authors were focused more on generating a pig model in this section rather than providing evidence of developing Usher syndrome in USH1C pig.
4. It is not clear from the methods section how F1, F2 het/homo offspring were genotyped. Provide the detailed method with representative gels.

5. Figure 1 is largely illegible even when viewed with a magnifying glass. A significant fraction of the figure should go to a supplement, where there is room for 2-3 panels per page.
6. Figure 2 and EV2. Be consistent with the graph's style. For example, some are with the absolute value on the figure and some are not. There are no "y" axis labels in the series of graphs, the labels indicated significance are also absent on some of them. There is no need to put the absolute p-value-the power of significance can be presented with asterisks and their meaning in the legend. Most lettering is too small.
7. Figure 3. Lettering in panel A is too small, about right in B and D, and too big in panel C.
8. Figure 4. Lettering is hugely inconsistent (compare size in A to that in G). The lack of care in figure preparation unfortunately reflects the lack of care in manuscript preparation.
9. Page 5 and Figure 3A, EV5E, F Authors presented a significant difference between wild-type animals and heterozygotes animals in ABR; also both wild-type animals and heterozygous had progressive hearing loss at 3 and 6 months through all tested frequencies and click data. How do authors explain this? Were the same animals tested at given time points? It is not clear from the figure legend whether "mean values" are presented with standard deviation or standard error?
10. Figure 3A. Either standard deviation or standard error (not clear from the legend) is quite large in USH1C; does it mean that some animals could hear at the age of 8 weeks? Were the same animals tested at 3 weeks? Was ABR performed across all frequencies?
11. Figure 3. The presented data on Figures 3C and 3D are confusing; the legend does not fully describe the figure. Do traces represent the mean, or this is individual recordings? Specify "n" in the legend. The left panel in D has three traces, which are very different. How do authors explain this variability? What does "dB" mean in the ERG figure? The same questions for figure D right panel. The authors also provided some statistical analysis of Ganzfeld ERG in the text, however, they did not clarify whether the results were statistically significant, and there are no relevant figures.
There are also many other questions related to ERG data: what statistical analysis was applied? How many animals were analyzed? Would be nice to see data not only for "a and b" wave amplitude but also the implicit times for peaks of "a and b" waves. Since the OCT does not show phenotype in USH1C, it would be good to see fundus microscopy of the central and peripheral retina, to evaluate the presence of any peripheral degeneration.
12. Page 6, Page 16, Authors refer to data that are "not shown". 'Data not shown' is not permitted: all significant data should be displayed in the main figures or Expanded View information.
13. Page 6. "In line with data from previous studies on other species (Reiners et al., 2003; Williams et al, 2009), WT pigs revealed harmonin abundance in the PRC layer, the outer limiting membrane, and outer plexiform layer (Figure EV6)." Unfortunately, the results from referenced studies in mice are not in the line with each other or with the current study. Williams et al, 2009: harmonin was found in the rod synaptic terminal, but also in the processes of the second-order neurons that extended into the invagination of each rod spherule. Indeed, the bipolar cell process contained more than twice the label counted in the rod spherule. Reiners et al., 2003: splice variants of harmonin were differentially expressed in the photoreceptor cell compartments. Where harmonin b isoforms were restricted to the light-sensitive outer segment, the harmonin a and c isoforms were more ubiquitously distributed in the photoreceptors. At the synaptic terminal of photoreceptor cells, harmonin a and c colocalized with myosin VIIa and cadherin 23.
11. Figure 4D. Please give a detailed protocol of how the analysis has been performed. How many replicates and animals underwent the analysis. This should be clearly indicated in the legend. The graph on the right shows no error bars. Was this measurement from one gel? I think it is very speculative to make a conclusion from n=1.
12. Page 8. The authors used human USH1C splice variant a1 for rescue experiments. Did this splice variant is most abundant in the pig's retina? Would be nice to see rtPCR data from the pig's retina that support the choice of isoform for gene therapy.
13. The whole section on testing therapeutic approaches, including Figure 6, should probably be removed. These tests are mostly not directly related to the animal model, and they are not related to each other. Save them for a paper that treats the pig model.

Other comments:
13. Page 2, "with at least 12 genes assigned to three clinical USH types". I would recommend change 12 to 10. There are five USH1 (myosin VIIa, harmonin, cadherin-23, protocadherin-15, and Sans), a putative USH1 (CIB2), three USH2 (usherin, ADGRV1, whirlin), and one USH3a (clarin-1) proteins (Géléoc G, El-Amraoui A, 2020). CIB2 is referred to as atypical USH as, unlike for all other 5 USH1 genes, several loss of function pathogenic variants in the CIB2 gene in both humans and mice were shown to cause the DFNB48 non-syndromic hearing loss, without loss of balance or vision (Booth et al., 2018; Michel et al., 2017).

14. Page 2, "The most severe of them is USH1, characterized by profound hearing loss from birth on, vestibular areflexia, and pre-pubertal onset of Retinitis pigmentosa (RP)". Change "Retinitis pigmentosa" to retinitis pigmentosa.

15. Page 3 "extended CTCF/cohesin-binding","3 intronic SNPs identified on the disease-allele". It is usually recommended to introduce every acronym before using it alone in the text.
16. Figure 1. Incorporate into Figure 1D the list of examined species to the right or left of the sequence data. Rephrase the 1D legend since it is not accurate. The gel on Figure1H represents the excision of the selection cassette. Specify in the legend the meaning of the grey boxes. What template was used as a control in this experiment?
17. Page 4 "(Figure EV1A-F)" Is this an independent sentence? There is no explanation in the text relevant to the Figure.
18. Page 4 "At an age of 3-6 months, we occasionally observed nystagmus (Movie 2), a common phenomenon of vestibular dysfunction and vertigo (Dougherty et al, 2020) and previously described in USH patients (Bonneau et al, 1993; Pieke-Dahl et al,

2000; Puffenberger et al, 2012)". The cited references (Pieke-Dahl et al., 2000; Puffenberger et al., 2012) do not accurately represent the statement in the text; I would recommend replacing them with more suitable references. Tiny point: "et alia" is abbreviated in citations as "et al." with a period. Even though the abbreviation is rather silly as it only saves one character, we have to do it correctly.

19. Page 4 and Figure 2A. Indicate in the text or the legend the age and number of animals that were tested, the frequency of the test for each animal.
20. Figure legend 2. "Impaired movement of USH pigs in 3-dimensional space". The title of the figure is not accurate. The legend also has references to non-existent figures such as "see Figure S2, Figure S3, S4".
21. Figure legend 2 "Mean values {plus minus} standard deviation are given by lines, mean values {plus minus} standard error are given by numbers, p-values for Mann-Whitney U-tests are indicated. Analysis included all runs, for normalized data sets, see Figure S4". Is this standard deviation or standard error? Please correct
22. FigureEV5 C. What genotype/age represents the figure?
23. Page 6 "Interestingly, spectral domain optical coherence tomography (Figure 3E) revealed only minor differences in retinal architecture between WT and USH1C pigs with good preservation of outer retinal layers containing the PRCs, suggesting a large therapeutic window". What age was tested? How many animals? It is good to see average thickness across multiple scans in both USH1C and WT
24. Figure 4C. There is some signal present in the panel with harmonin staining. Is this a real signal or background? It would be good to see co-staining with nuclei markers and photoreceptor markers to better see architecture of the retina and evaluate the quality of the sections.
25. Figure EV 6A the outer limiting membrane (arrow). There is no arrow on the figure.
26. Page 7. "While the structure and arrangement of PRCs appeared grossly intact in USH1C pigs (Figures 4e, S7a), subcellular analysis revealed specific disruptions in the architecture of compartments comprising harmonin." This statement is very speculative. No data that support this statement. Also, Figure S7a is not found. Probably "containing" is more appropriate than "comprising."
27. Figure 5 C, E and the relevant legend is incomplete. Was mean presented with SD or SE? It is hard to believe just looking into the graph that the difference is significant. The data has to be reanalyzed and properly presented. The same problem is with FigureEV7 B and C (right panel). Authors also should provide the detailed protocol of quantitative fluorescent microscopic analysis and transmission microscopic analyses that they performed, otherwise, it is not possible to evaluate the methodology.
28. Figure EV7 C. Would it be possible that the cone synaptic pedicles is being wider is an artifact (if the sectioning was more tangentially oriented then they could appear longer when they are not) ?
29. Page 7. Specify in the text or in the legend the dose/volume that was injected for each vector. Were samples immunostained to amplify GFP signal? It is always beneficial to co-stain with DAPI and other markers to evaluate retina architecture.

Referee #3 (Remarks for Author):

Review of the manuscript "Early Disruption of Photoreceptor Cell Architecture and Loss of Vision in a Humanized Pig Model of Usher Syndrome" by Dr. Klymiuk and colleagues [EMM-2021-14817]
General comments:
The authors generated a pig model for Usher Sydrome type 1C by introducing a human mutation into the harmonin-encoding gene. The study describes in detail the phenotype of the USH1C pigs that reflects quite well the syndromic disease of human patients: hearing defects, vestibular dysfunction and visual impairment. The model and the results are convincing and well described.
However, while this USH1C pig model is certainly very interesting for future pre-clinical studies, I don't think that the data so far presented in this study have substantially improved our molecular understanding of the USH1C disease as claimed in the last paragraph of the Discussion. The study is essentially based on visual and motion behavior. The therapeutic approaches described in the manuscript either with AAV strategy or CRISPR Cas9 technology also appear to have been treated rather superficially in cell culture and require further in-depth investigation in vivo.
Specific comments:
1) A single allele insertion of the human mutation gave a progressive "dominant-negative" phenotype in Heterozygote pigs as indicated by the age-dependent increase of the hearing thresholds (Figs3A and EV5). Could you please discuss this point in regards to the human disease and to the development of future genetic therapy.
2) It would have been interesting to plot and compare the amplitude of the ABR waves I (hair cell synapses) and V (central synapses) as a function of sound intensity to determine whether the phenotype affects all sound intensities, in particular in the Hetero at various age. Indeed, since genetic deletion of harmonin has been shown to also affect the hair cell ribbon synapse in mice, analysis of the ABR wave amplitudes could have been informative regarding a possible progressive hair cell synaptopathy in pigs. Also, recordings of the DPOAEs, reflecting the mechanical amplification by the OHCs could have been interesting in order to get a more complete assessment of the auditory function.
3) Throughout the text and figures, it is often difficult to know the exact number of animal studied. Please give the N number in the text for each test - such as for those described page 6 and Fig3.
4) Page 8: Citing Nagel-Wolfrum et al. as unpublished data is insufficient, please give a more detailed description of AAV vector

encoding USH1C variant a1 and the reasons of choosing this variant.
5) Give a more precise description of how the length of the primary cilium in primary skin fibroblasts was determined ? The use of this single parameter as a marker of restoration after gene therapy seems not convincing.
Minor points:
6) Page 6, in the text top paragraph, give the SD value of the a-wave amplitude with a decimal 10{plus minus}0.X?
7) page 5, second para, replace "sensoneural" by "sensorineural"

Klinikum rechts der Isar
Technische Universität München
DIE DEUTSCHEN UNIVERSITÄTSKLINIKA®

**Klinikum rechts der Isar**
**Anstalt des öffentlichen Rechts**

**Klinik und Poliklinik für**
**Innere Medizin I**

**Univ.-Prof. Dr. med.**
**Karl-Ludwig Laugwitz**
Director

**Prof. Dr. Nikolai Klymiuk**
Head of the TUM Chair for Large Animal Models in Cardiovascular Research

**Dr. rer. nat. Christiane Schmautz**
Scientific Coordinator

Ismaninger Straße 22
81675 München

E-Mail: n.klymiuk@tum.de

Tel: +49-151-522-95-921
www.mri.tum.de

Klinikum rechts der Isar – TUM – (Med I) – 81675 München

To

Prof. Lise Roth

EMBO Molecular Medicine´s Editor

München, 09.08.2021

Dear Prof. Roth,

Referring to the reviewer´s comments on our manuscript EMM-2021-14817 **"Early Disruption of Photoreceptor Cell Architecture and Loss of Vision in a Pig Model of Usher Syndrome"** we wanted to discuss some essential aspects of our work and their relevance for the manuscript. Most of all, it is essential to consider that we are dealing with a large animal model. The value of such models for translational research is meanwhile (mostly) out of debate, and some ground breaking studies have been published by our group and other labs. Still, the effort of conducting such experiments is often underestimated, influenced by the circumstances under which studies in mice are conducted.

Regarding the USH1C pig model, we are on our way to establish routinely producing breeding herds to face the demands for pre-clinical therapy studies. In the past months, more than 15 USH1C piglets have been born and first therapy studies will be initiated by end of September. These experiments will be done within a project aiming at developing gene therapy for USH1C patients. We have to take into account an observation period of ≥ 6 months as a pre-requisite of the Food and Drug Administration for accepting such studies for pre-clinical research and further consideration of future clinical trials. Considering a further 3-4 months for evaluating these data, a consistent result from the first gene therapy study will be available not earlier than by fall 2022, even under best circumstances. For this reason, we can only provide evidence that the USH1C pig model will be a valuable model for future pre-clinical studies, as we have done in the manuscript (Figure 6). In the revised version we provide additional data, generated by sub-retinal injection of USH1C_a1 expressing AAV vectors into one eye of a single 2.5 years-old USH1C sow while the other eye remains an untreated control. After an observation period of 12 months, we observed a significantly improved ERG signal (120µV vs 30µV), abundance of harmonin in the treated eye, but not in the control eye and also morphological exploration. The question remains if this is interesting enough for illustrating the translational potential of the model or if this is some preliminary data that should be spared for a future publication. From our side

as responsible authors, we are convinced that those data should not be hidden for months (or years), but we propose that the reviewers and the editors of EMBO Mol Med can estimate much better whether it is worth including this part of the manuscript and whether the manuscript is valuable for publication in the journal.
The fact that only small animal numbers were available until breeding programs are now taking off, dictated also our ambitions on the phenotypical characterization of the model. For this reason, we put our main effort in examinations that required robust statistical evaluation, such as the behavior tests (n=5 WT and n=5 USH1C animals, each in 20 runs) or the hearing threshold (n=6 homUSH1C, hetUSH1C, WT) while many of the other experiments have been done at lower resolution (n=4 eyes from n=2 animals in WT vs USH1C pigs), preventing "true" statistical examination. We are convinced that the comprehensive and multi-disciplinary evaluation of this small pool of animals prevents any misinterpretation of the phenotype in USH1C pigs.
Finally, we are eager to overcome concerns about the extent of the manuscript. Depending on EMBO Mol Med´s editorials´ opinion we can easily re-write the introduction section and describe the size, role and localization of harmonin in more detail and point to the specific R31X mutation in patients, as referee #2 expected. Further, the relevance of nystagmus or the findings on a presumably increased hearing threshold in heterozygous carriers can be discussed comprehensively in the discussion section.

Please let us know, if addressing these points would meet the editorial boards´ expections and promote our ambitions on publishing at EMBO Mol Med.

Sincerely yours,

Nikolai Klymiuk
(on behalf of the co-authors)

Vorstand:
Prof. Dr. Markus Schwaiger
(Ärztlicher Direktor, Vorsitzender)
Dr. Elke Frank
(Kaufmännische Direktorin)
Silke Großmann
(Pflegedirektorin)
Prof. Dr. Bernhard Hemmer
(Dekan)

Bankverbindung:
Bayer. Landesbank Girozentrale

BIC: BYLADEMM
IBAN: DE82 7005 0000 0000 0202 72
USt-IdNr. DE 129 52 3996

Dear Nikolai,

I have now received feedback from the three referees:
They replied:

Referee #1:
we appreciate the author concerns related to working with large animal models. Therefore, if the authors address our concerns (including experimental) which do not involve injection of additional animals this would be fine with us.

Referee #2:
I understand the authors' ambitions to publish the animal model and a therapeutic approach together. However, the therapeutic results included in the initial submission are mostly not directly related to the animal model, and they are not related to each other. It only confuses the readers, in my opinion.
The data that they could add to the paper is based on one eye and is preliminary. With n=1 the study is underpowered, it will be statistically inconclusive and may make the whole conclusion wrong. Hence, I think it is not sufficient for publication in EMBO Molecular Medicine.

Referee #3:
If the authors can provide additional data generated by sub-retinal injection/ /in vivo/ of USH1C_a1 expressing AAV vectors, I think that the revised study becomes interesting enough for showing the translational clinical potential of their model. We could then potentially accept a revised version of the study.

Based on these comments and after discussion with my colleagues, we agree that additional in vivo experiments will not be required for consideration in EMBO Molecular Medicine. Furthermore, we think that the additional data on sub-retinal injection of USH1C_a1 expressing AAV vectors into one eye should be included in the manuscript as a supplement, and with clear indications in the text that these are preliminary data.

I hope this helps,

With my best wishes,

Lise

-----
Lise Roth, PhD
Editor
EMBO Molecular Medicine
l.roth@embomolmed.org

General re-structuring of the manuscript: the introduction section was extended from 266 to 902 words to meet the desire for more comprehensive information on USH1C and the translated protein variants of harmonin. Further, composition and order of figures was slightly adapted to meet EMBO Mol Med´s requirements for Figures, EV Figures and Supplemental Figures. In detail, Figure 1 was reduced in its content and enlarged for better visualization and contain the former A-D subfigures. Figures 2 & 3 remained in their original composition. Figure 4 was restructured to comprise molecular analysis of USH1C pig retinas. Data on GFAP regulation were shifted to a new Figure 5 that illustrates also changes at cone synapses. Figure 6 now comprise what was formerly in Figure 5, with some adaptations according to the reviewers´ suggestions. A new Figure 7 describes therapeutic approaches, which was formerly in Figure 6, with the addition of data from an exploratory gene therapy study. Figure EV1 was complemented by former Figure 1F-H. Figures EV2-4 were moved to the supplementary material. New Figure EV2 now comprise the content of former EV5 (hearing deficit). Accordingly, new Figure EV3 is former EV6 (localization of harmonin in retinal structures). Figure EV4 is now what was Figure EV8 before (gene repair). Figure EV5 comprise new data on the gene therapy approach in vivo. The content of the former Figure EV9 has been moved to supplemental material.

***** Reviewer's comments *****

**Referee #1 (Remarks for Author):**

*In this paper the authors describe the generation of a pig model of USH1C through insertion of a human mutation in the pig USH1C gene which encodes for harmonin. Mouse models of Usher syndrome which phenocopy the retinitis pigmentosa observed in patients are lacking, presumably due to the absence of calyceal processes in mice that are present in pigs. Therefore, such large animal model responds to an important unmet need which limits in particular the development of novel therapies. The authors performed a detailed characterization of the phenotype developed by USH1C pigs and, interestingly, they observed both retinal and hearing defects, some of them already present at young ages. Although no evident loss of photoreceptor cells has been observed, rearrangements of the OS structure and increased length of the connecting cilium, in line with the proposed retinal function of harmonin, have been identified. The characterization the authors have performed is very comprehensive and the data is sound.*

*The manuscript could be improved by taking into accounts the comments below:*

*1. The manuscript suffers from lack of information on the actual number of eyes analyzed. Please clarify in the legend to the Figures 3, 4 and 5 the number of pigs/photoreceptor analysed for each panel.*

→ We recognize the shortage of the manuscript in this context, which was also criticized by referee #2 pts 11, 14, 23 and referee #3 pt3. We followed their suggestions including the number of animals, retinae, technical repeats and structures analyzed in the figure legends of the revised version of the manuscript. Due to the lack of larger animal cohorts, the development of analysis tools and – protocols as well as in the ambition to reduce numbers of terminal experiments, we depicted representative figures for illustrating the general findings of the phenotype at distinct ages. Only in cases when larger cohorts of animals were available, analysis tools could be consistently used and anesthesia was to be performed without risk, we explored pigs at numbers that facilitate sufficient statistical analysis. We clarified this in the respective figure texts: For analysis of the hearing deficit (Fig. 3A), we focused on early ages to address the congenital nature of the defect. We show representative recordings of 2 WT and 2 USH1C pigs at an age of 3 weeks, the earliest possible time point we have examined (left panel), requiring euthanasia and allowing post mortem sampling of cochleae. In older animals, intermediate

measurements facilitated the examination of larger animal numbers (indicated in the middle and right panel of Fig 3A). Clinical vision was preferentially examined at older ages, considering the progressive nature of the retinal phenotype. At 12 months of age, 2 WT and 2 USH1C animals were comprehensively examined by combined gfERG, mfERG (light and dark adapted) and OCT (Fig 3B-E). Evaluation of tissue samples from 1-2 animals allowed the documentation of absence of harmonin and presence of the USH proteins SANS, whirlin and myosin7a in Figure 4. The examination of technical replicates (TR) excluded technical artifacts. The same is true for (new) Figure 5 and Figure 6 (former Figure 5). In both Figures, examination of multiple tissue sections or cells facilitated statistical examination.

*2. Similarly, in page 4 the authors state "we occasionally observed nystagmus"; please clarify if occasionally refers to the number of animals or to the timing of the observations.*

→ The rapid eye movement was observed during routine inspection of the animals for general health conditions. We observed this finding in some, but not all animals. In the affected animals it was not a regular finding, but appeared from time to time. This is in line with the observation of rare mild spontaneous nystagmus in USH patients while both caloric and rotational testing do not evoke a nystagmus, indicating an asymmetrical bilateral vestibular hypofunction (Moeller et al 1989 doi:10.1288/00005537-198901000-00014, Baloh et al. doi: 10.1212/01.CON.0000418371.49605.19). Considering the sparse literature on the topic, we interpreted this finding as an interesting side effect in USH1C pigs, but refrained from a more systematic analysis because of the limited number of observations. From the available animal population, we cannot judge if this nystagmus followed a regular pattern or if it appeared at increased frequency from time to time. We agree with the referee that this is somehow unsatisfying from a scientific standpoint, but we found the correlation to the literature on this topic interesting enough to mention this finding in the manuscript, in particular with respect to a potential vestibular origin. In absence of more precise information we used the term "occasionally" to describe the infrequent occurrence of this pathology. We have discussed this aspect with two experts in the field of vestibular disturbance, Profs. Straka and Dlugaiczyk who have specified this problem. In the section on the nystagmus, we also considered referee #2´s pt 22 criticism and specified the respective section into: *"At an age of 3-6 months, we occasionally observed a spontaneous nystagmus of the eyes (Movie 2) in some animals. This is very likely caused by caused by an asymmetrical tonic firing of vestibular afferents from the right and left labyrinth (Baloh et al, 2012; Dougherty et al, 2020), and has been anecdotally described in USH patients (Bonneau et al, 1993; Moller et al., 1989; Shibasaki et al, 1978)."*

*Lastly, from the legend to Fig.2 it appears that each dot in the graph represents a run; if this is correct, please clarify from how many animals these runs have been obtained.*

→ This point refers also to referee #2 pts 6, 23. Indeed, each dot is single run conducted by a pig that met our inclusion criteria (see Supplementary Materials and Methods). We describe the constellation of examined groups and the number of test performed in the dark and under light conditions in the figure text. As pointed out at in our answer to referee #2 pt.6, we did not feel capable of performing serious statistical tests for significance for the barrier test (Figures 2A, S4) as we examined uneven und small groups of animals there.

*3. Fig 3B-D. ERG data from all the analysed pigs should be shown to better understand the variability of the phenotype. Accordingly, data reported in the text in page 6 (Sentence: "USH1C animals showed > 70% reduction... and a 60% reduction in the respective b-wave amplitude (86{plus minus}49 µV in USH1C vs. 215{plus minus}50 µV in WT animals")) should be shown in a dot plot graph where data from each animal can be separately appreciated.*

→ We thank the referee for his concerns, which are in line with referee #2 pt11. We decided to give the data from ERG measurements in table format in the new Figure S7 as the most comprehensive

format. We hope that referee 1 appreciates this as superior to the suggested dot plot presentation. We hope that the referee also agrees that for the limited number of animals examined (2 USH1C and 2 WT pigs), we give mean values plus/minus standard deviation, but we do not perform an further statistical analysis.

*4. Fig.EV7B-C the differences are significant yet very subtle, is this biologically relevant?*
→ We agree that the question for biological relevance of subtle differences in certain parameters is a delicate one. This is particularly true for the activity of harmonin in synapses as its activity as scaffold proteins interferes with other synaptic components, for example channels and adhesion molecules, and small changes in the synapses may therefore have substantial consequences. It can be assumed that the size of the synapses is affected by the size of the scaffold in the active zone of the synapses, which also scales with the overall probability of an evoked release of neurotransmitters in response to an action potential (discussed in Petzoldt et al. 2016 doi: 10.1016/j.conb.2016.04.009). Previous studies indicated that harmonin is part of the presynaptic scaffold of ribbon synapses (Gregory et al. 2011, doi:10.1038/nn.2895) and regulates of the function of ribbon synapses in auditory hair cells by binding/scaffolding of voltage gated $Ca^{2+}$-channels (Gregory et al., 2013, doi: 10.1113/ jphysiol.2013.254367).
As cone synapses are much larger in diameter and our previous analysis of human photoreceptor cells has revealed that harmonin is more abundant in cone pedicles, compared to rod synapses in primates (Nagel-Wolfrum et al. 2021, doi:10.1101/2021.08.27.457962), we focused our analysis on synaptic pedicles of cones by light and electron microscopy. We agree that the analysis of dimensions of structures like synapses in 60 nm ultrathin sections by TEM depends on the orientation and the profile of the sections. Therefore, we measured only the widest synaptic pedicles in the ultrathin sections and show representative images in Figure 5D. In an alternative approach, we relied on light microscopy analysis of fluorescence-stained synapses in 10 µm thick cryosections, in which the orientation of the sections does not play such a significant role for the measurements. In addition, we were able to automatically measure the dimensions of hundreds of synapses in the retinal sections using the Fiji script, which we describe in the Material and Methods section. For this reason, we are convinced that our analysis of synaptic width in USH1C reveals a novel and relevant aspect of the disease, indicating a potential explanation for the reduced signaling from the retina to the brain, as measured by ERG, although the light sensing structure appears grossly intact USH1C retina.

*5. Although the characterization of the animal model is very comprehensive and solid, potential therapeutic approaches have been evaluated only ex vivo. Further in vivo data would definitively strengthen the paper. Along a similar line please comment why genome editing has been selected rather than AAV-mediate delivery of wild-type harmonin.*
→ This point is reflected by all referee´s and the editor´s estimation and we agree that the perspective of conducting pre-clinical studies in vivo is one of the major assets our USH1C pig model. Such studies in large animal models are becoming increasingly popular in biomedical science. It must be considered, however, that they are extremely costly and time-consuming. Based on our previous experience in pre-clinical studies on pig models (Langin et al. Nature 2018 doi: 10.1038/s41586-018-0765-z, Regensburger et al. Nat Med 2019 doi: 10.1038/s41591-019-0669-y, Moretti et al. Nat Med 2020 doi: 10.1038/s41591-019-0738-2.) 2-3 years (at least) must be considered for planning, initiating, executing and comprehensively analyzing the data. Here, it was our ambition to present the availability of the first fully sufficient pig model for USH1C, and document the generation of the model, its phenotypical characterization, but we find it also important to illustrate the potential of the USH1C pig model for future studies. Regarding the latter, we show that we have mastered several essential pre-requisites: (i) we document the establishing of breeding herds by providing a pedigree genotyping data in new Figures S2. (ii) we confirm that in vivo transduction of the retina must consider the AAV serotype (new

Fig. 7A was improved according referee #2, pt33). (iii) by Sanger sequencing of RT-PCR products (according referee #2 pt12´s and referee #3 pt4´s concerns), we confirm that harmonin_a and -_b splice variants appear in all tested organs. As we show, however, in new Figures 4A-C and S9, harmonin_a1 seems to be the most abundant form of the protein. (iii) we have now also put emphasis on gene therapy by sub-retinal AAV injection in vivo and reveal restoration of harmonin expression and improvement of ERG data in treated eyes (new Figures 7D and EV5). In this context we find it remarkable and pointed out in the discussion section that classical gene therapy still has a substantial effect even when applied at a later age (2.5y, 3.5y) at a time point when vision is already significantly impaired. (iv) Although gene augmentation therapy by AAV application seems the most promising therapy for recessive retinal disorders at the present time point, we have considered the option of gene repair as an even more sustained therapy for post-mitotic cells in the retina in the design of our model (Figure 7B). We find it relevant to document the potential of this approach, as our in vitro data confirm the accessibility of the USH1C locus for CRISPR tools and indicate an outstandingly high rate of gene repair (approx. 40% of examined cells). We are aware that the non-regenerating nature of the PRC (i.e. no active cell cycle) might limit HDR in PRC, but it is not unlikely that one of the numerous attempts that are presently followed in the field will overcome this issue. Comparing some of these approaches in the USH1C pig in vivo will indeed be a valuable future study.
We are aware that these data do not meet the requirement of a comprehensive pre-clinical study, but we strongly believe that they are extremely valuable to illustrate the translational potential of the novel USH1C pig model.

*Additional minor criticism:*
*6. Supplementary figures are sometimes erroneously referred to as "Fig. S" instead of "Fig. EV"*
→ we apologize for the inconvenience. We have corrected these errors in the revised version of the manuscript.

**Referee #2 (Remarks for Author):**
*The manuscript "Early disruption of photoreceptor cell architecture and loss of vision in a humanized pig model of Usher syndrome" from Grotz et al presents results of the retina and inner ear phenotype in the USH1C pig model. Usher syndrome (USH) is a major cause of deaf-blindness in humans, affecting ~400,000 patients worldwide. Three clinical subtypes, USH1-3, have been defined, with 10 USH genes identified so far, with USH1 being the most severe. Usher type 1 patients can benefit from cochlear implants, however, there are currently no biological treatments available for visual dysfunction. A good animal model for testing retinal therapy would be of value.*
*In this study, the authors introduced a human mutation into the harmonin-encoding USH1C gene in pigs and generated the first translational animal model for USH type 1 which potentially mimics the full syndromic phenotype in human USH1 patients (characterized by profound hearing loss from birth, vestibular dysfunction, and retinitis pigmentosa). The authors investigated the retinal phenotype of the transgenic USH1CR31X pig analyzing the disease progression in relation to the morphology, the molecular changes and the retinal function.*

Mayor comments:
*1. The transgenic USH1C pig that has been generated is a unique animal model that potentially develops a phenotype similar to that in patients and is beneficial for examining gene therapy treatments of USH1C. The amount of generated data are impressive. However, the manuscript is not well written, and some results are not clearly presented and not convincing, or not fully described. I would recommend authors rewrite the manuscript and put extra effort into organizing data and making clear figures.*

→ We apologize for the inconvenience and have followed the referee´s suggestion. We kept the main structure of the manuscript, but have worked on improving the data presentation in the Figures and Figure captions, we have addressed the referees´ and the editor´s concerns in the Figure captions and gave a more comprehensive description of data in the results section.

*2. The manuscript lacks an overview of the most relevant research that has been conducted in the inner ear and retina. I recommend giving a more thorough intro to the USH1C gene and harmonin (size, localization, and function, known splice forms, expression profile during the development in the inner ear and retina, mouse models if any); the R31X patient-specific mutation, patient visual phenotype and etc. Support the background information with relevant references.*
→ We followed the referee´s suggestion and substantially expanded the introduction (increase from 266 to 902 words), providing the desired information on splice variants, protein localization, the poor relevance of mouse models for the retinal phenotype etc. We presume that referring to the distinct localization of the USH1C gene in human (chr11:17.5Mb), murine (chr7:45.9Mb) and pig USH1C (chr2:41.6Mb) along with the different biologically verified and bio-informatically predicted splice variants that are indicated for the respective species in distinct data bases and in the literature might confuse one or the other reader. This is particularly true for the partial humanization of the porcine USH1C gene. For this reason, we hope that the referee appreciates the omission of too detailed information on genomic properties and our focus on the most relevant and commonly accepted aspects of major splice variants and their biological function. We also put some more emphasis on patient-specific mutations and visual phenotype and put these aspects into the context of our own results in the discussion section.

*3. Page 3, "A patient-specific disease-causing segment in USH1C leads to Usher syndrome in pigs". The title does not represent the information described in the paragraph. I would change it since the authors were focused more on generating a pig model in this section rather than providing evidence of developing Usher syndrome in USH1C pig.*
→ we agree with the reviewer and have followed the referee´s suggestion and accordingly changed the title of the sub-section into “Genetic modification of the porcine genome by introducing a human segment carrying a patient-specific mutation into the USH1C gene:”

*4. It is not clear from the methods section how F1, F2 het/homo offspring were genotyped. Provide the detailed method with representative gels.*
→ We now include a pedigree and genotyping of F0 animals and representative litters from the F1 and F2 generations in the supplemental material (Figure S2). During breeding it became obvious that the modification of the porcine USH1C gene was distinct on the two USH1C alleles in some of the founder animals. In F1, some of the offspring carried the humanized segment of exon2 whereas other animals retained only one allele with porcine exon2, as verified by qPCR-based allele-number detection, at the genomic level. This was verified at the transcriptional level in different organs (Figures S3); the first set of animals showed a consistent splicing of the humanized exon 2 into USH1C transcripts while the other group of heterozygous animals showed a pattern of a consistent splicing of porcine WT USH1C transcripts as well as a bystander sequence that represented transcripts lacking exon 2. Both groups of animals occurred at an approximate 50:50 ratio, suggesting that the parental founder animals comprised correct recombination event on one allele (“R31X allele”), whereas the other suffered a larger NHEJ-mediated disruption, deleting a yet undefined genomic fragment, including exon 2 (“del allele”). The del allele appears at much lower frequency in the Sanger sequencing of RT-PCR products, presumably as a result of nonsense-mediated decay. Both, the R31X allele as well as the del allele abrogate harmonin translation, first by the STOP codon and second by a frame-shift mutation. Consequently, homozygous F0 and F2 animals showed consistent congenital hearing disabilities and

vestibular dysfunction, independent of the allelic constellation. We have documented these findings in a new Figure S2 and S3 and in the results section. For future studies we are working on a consistent breeding strategy for producing consistent allelic constellations in USH1C pigs. It needs to be considered that this asymmetric modification of the porcine USH1C alleles did not occur in all founder animals. Very likely, founder animals descend from distinct single cell clones that were mixed for the cloning procedure to achieve a higher efficacy in the SCNT procedure. In vitro testing of gene repair was, however, examined in primary cells of founder pigs carrying the humanized mutation on both alleles.

*5. Figure 1 is largely illegible even when viewed with a magnifying glass. A significant fraction of the figure should go to a supplement, where there is room for 2-3 panels per page.*

→ we apologize for the inadequate quality and have followed the referee´s suggestion and considerably improved Figure 1, enlarged the remaining part (particularly letters and figures) and transferred some components into EV figures. According to (minor) point 13, we also adapted Figure 1D to comprise the species names, specified the figure text and improved presentation of Cre-mediated excision of the neo selection cassette (now Figure EV1F-H).

*6. Figure 2 and EV2. Be consistent with the graph's style. For example, some are with the absolute value on the figure and some are not. There are no "y" axis labels in the series of graphs, the labels indicated significance are also absent on some of them. There is no need to put the absolute p-value-the power of significance can be presented with asterisks and their meaning in the legend. Most lettering is too small.*

→ We apologize for the somewhat inconsistent quality and improved Figures 2, S4, S6. All graphs are now consistently structured, labelled and illustrated with numbers at larger scale. In the barrier test (Figures 2A and S4) we examined uneven and small groups of animals, for which reason we did not feel capable of calculating statistical significances. We describe the constellation of the respective groups in the figure text and mention also that data plots represent individual runs that met our inclusion criteria defined in the Supplementary Materials and Methods.

*7. Figure 3. Lettering in panel A is too small, about right in B and D, and too big in panel C.*

→ We apologize for the inconsistent quality and thank the referee for the suggestion and improved lettering in Figure 3 (as well as in Figure 4).

*8. Figure 4. Lettering is hugely inconsistent (compare size in A to that in G). The lack of care in figure preparation unfortunately reflects the lack of care in manuscript preparation.*

→ We apologize for the inconsistent quality and thank the referee for the suggestion. We have now put emphasis on a consistent and readable layout of the new Figure 4 and the data of GFAP expression that are now in Figure 5A, B).

*9. Page 5 and Figure 3A, EV5E, F Authors presented a significant difference between wild-type animals and heterozygotes animals in ABR; also both wild-type animals and heterozygous had progressive hearing loss at 3 and 6 months through all tested frequencies and click data. How do authors explain this? Were the same animals tested at given time points? It is not clear from the figure legend whether "mean values" are presented with standard deviation or standard error?*

→ We thank the reviewer for indicating this aspect; we now clarify several aspects regarding methodology and interpretation.

Regarding a reduced auditory sensitivity of heterozygous individuals, we were able to reproduce the difference between heterozygous and WT pigs with different methods (tone audiometry vs. clicks) and in different experiments (3 months of age vs. 6 months of age) (EV2 E, F). Although differences are

small (approx. 10 dB) and might not be a relevant impairment in the daily life of heterozygous USH1C carriers, we find it important to report that heterozygous USH1C+/- pigs had slightly elevated hearing thresholds compared to WT pigs, because this finding indicates that harmonin is necessary at considerable amounts for proper function, at least in the cochlea. Of note, we did not examine a single cohort of animals at different timepoints, but compared distinct groups of pigs at the respective ages. The consistently enhanced hearing threshold of het USH1C pigs vs. WT controls in distinct groups at different ages support our interpretation.
Regarding the proposed progressive hearing loss of WT and het USH1C pigs over time, we would be, however, tempted to follow the referee´s conclusions. Again, we observed a small difference of 10 dB, but in contrast to the measurements of WT vs. het animals that were conducted at the same time point at the same conditions for a given age, we can only compare the data gained at distinct time points without clear standardization. It should be noted that the experimental situation changes considerably between 3 and 6 month old pigs simply due to the massive body growth (3 months: 25 kg, 6 months: 100 kg). Measurement of ABR hearing threshold is highly dependent on detection of the signal based on amplitude and amplitude/background ratio. ABR is by its nature a far-field recording technique, given that recording electrodes are placed subcutaneously at the base of the ear and in substantial distance to the neural origin of the signal in the cochlear nerve. Consequently, amplitudes are usually much smaller in larger animals when there is more bone and tissue between recording electrode and signal origin. Consequently, we cannot exclude that slightly higher hearing thresholds in older animals of much larger size (100 kg vs 25 kg) are just a reflection of the different experimental situation rather than an indication of a progressive hearing loss. Further, different frequencies were applied to the animals at different ages due to distinct stimulation protocols (see x-axis in Figures 3B and EV2E, F). Therefore, we would be reluctant to over-interpret the data and hope that the referee agrees with the restriction of to our focus on the verified difference between WT and het USH1C pigs. Nonetheless, we have slightly adapted the respective section in the results part of our manuscript and clarified that: *"The hearing threshold of heterozygous USH1C+/- pigs was slightly elevated compared to WT littermates at distinct ages (Figures 3A, EV2E, F). Hearing thresholds for both WT and heterozygous animals slightly improved with increasing test frequency, while thresholds in USH1C animals deteriorated further for higher frequencies. In line with their profound sensorineural hearing loss, USH1C piglets …"*.
In the figure caption, we now clarify that mean values (MV) plus/minus standard deviation (SD) are given for the ABR.

*10. Figure 3A. Either standard deviation or standard error (not clear from the legend) is quite large in USH1C; does it mean that some animals could hear at the age of 8 weeks? Were the same animals tested at 3 weeks? Was ABR performed across all frequencies?*
→ We thank the referee for this point. Indeed, we observed increased standard deviation (SD) for the homozygous USH1C animals at eight weeks. While 4 of 6 animals had threshold values > 100 dB SPL in all frequencies and in the click test, 2 animals responded at somewhat lower intensities (approx. 80 dB SPL, see example given in Figure EV2C). At 3 weeks of age, we examined distinct animals (those that were used for ultrastructural examination of cochlea cells (Figure EV2B). The examined number of animals is, at present, too low to consider a (partially) remaining hearing ability. For this reason, we found it adequate to emphasize the severe hearing disabilities of USH1C animals early in their life, but would refrain from claiming complete deafness at the time of birth. We would, however, agree that a potential hearing ability early after birth, as this is of high relevance for defining the therapeutic window of opportunity for gene therapy treatment of the inner ear defect in human patients, even if this extended only during the first weeks of life, as this is of considerable relevance for defining the therapeutic window of opportunity for gene therapy treatment of the inner ear defect in human patients. Such studies, however, will need comprehensive investigation of ABR on larger animal

numbers at distinct time points and will involve also carefully selected WT pigs, at least age-matched animals and at best litter mate controls. ABR was performed in all animals at the indicated frequencies.

*11. Figure 3. The presented data on Figures 3C and 3D are confusing; the legend does not fully describe the figure. Do traces represent the mean, or this is individual recordings? Specify "n" in the legend. The left panel in D has three traces, which are very different. How do authors explain this variability? What does "dB" mean in the ERG figure? The same questions for figure D right panel. The authors also provided some statistical analysis of Ganzfeld ERG in the text, however, they did not clarify whether the results were statistically significant, and there are no relevant figures.*

→ This point has been also raised by referee #1 pt3. We have clarified the number of examined animals (2 USH1C and WT animals, respectively) in the figure caption. The observed individual difference between the animals might have many reasons, including individual differences, experimental differences or else. The finding of reduced responses in the distinct dark and light adapted stimulation, however, is consistent and in line with reduced vision in the behavior tests and the findings at morphological level, but the low number of animals and the variability within "groups" support our reluctance for extended statistical calculations. We have reduced the misleading "dB" term from the figure and give, according to the recommendation of referee #1 pt3, a more comprehensive overview of the data in table format.

*There are also many other questions related to ERG data: what statistical analysis was applied? How many animals were analyzed? Would be nice to see data not only for "a and b" wave amplitude but also the implicit times for peaks of "a and b" waves. Since the OCT does not show phenotype in USH1C, it would be good to see fundus microscopy of the central and peripheral retina, to evaluate the presence of any peripheral degeneration.*

→ We agree with the suggestion to extend the documentation in our ERG. So, we have implemented all ERG data on 1y old USH1C vs WT pigs in table format as a new Figure S7 and the new ERG on AAV-treated animals in Figure EV5C. In cSLO we document that there were no signs of gross degeneration in the central retina (new Figure S8). There are also no signs of degeneration in the periphery (observation by MDF).

*12. Page 6, Page 16, Authors refer to data that are "not shown". 'Data not shown' is not permitted: all significant data should be displayed in the main figures or Expanded View information.*

→ This aspect has been corrected. As pointed out, the cSLO are now documented in Figure S8. For the ERG-examination of 3 week old piglets we did not record data. Thus, we deleted the respective sentence form the manuscript.

*13. Page 6. "In line with data from previous studies on other species (Reiners et al., 2003; Williams et al, 2009), WT pigs revealed harmonin abundance in the PRC layer, the outer limiting membrane, and outer plexiform layer (Figure EV6)." Unfortunately, the results from referenced studies in mice are not in the line with each other or with the current study. Williams et al, 2009: harmonin was found in the rod synaptic terminal, but also in the processes of the second-order neurons that extended into the invagination of each rod spherule. Indeed, the bipolar cell process contained more than twice the label counted in the rod spherule. Reiners et al., 2003: splice variants of harmonin were differentially expressed in the photoreceptor cell compartments. Where harmonin b isoforms were restricted to the light-sensitive outer segment, the harmonin a and c isoforms were more ubiquitously distributed in the photoreceptors. At the synaptic terminal of photoreceptor cells, harmonin a and c colocalized with myosin VIIa and cadherin 23.*

→ We apologize for the potential confusion. The exact localization of harmonin in the complex retinal structure is indeed an elusive problem. The distinct opinions on this issue in the literature might arise

from a number of different reasons, but mainly we presume that the specificity of the antibody is a most critical parameter. Mostly, they are initially tested under artificial conditions (e.g. by overexpression in immortal cell lines) and so this does not necessarily mean that there will be no cross-reactivity in specific organs. Further, specificity of an AB in one species does not necessarily exclude cross-reactivity in another. Finally, there might be indeed distinct localizations of the same protein in different species, in particular if the cellular anatomy differs, as it is the case for CP in the retina. By using the USH1C knockout pig, we have the unique possibility for ultimately testing the specificity and can verify harmonin signals as true, when they are lacking in USH1C pigs. Based on this very robust experimental setup, we are convinced of our localization experiments, but we would refrain from discussing too much about problematic parameters in other studies. We refer, however, to the ambiguous situation by a specific paragraph in the introduction:

*"Harmonin is also consistently localized to the ribbon synapses of hair cells and PRCs (Gregory et al, 2011; Reiners et al, 2005; Williams et al, 2009a) whereas data on harmonin abundance in other compartments of the retina were contradictory in rodents (Reiners et al, 2003; Reiners et al., 2005; Sahly et al., 2012a; Williams et al., 2009a). In species with pronounced CP structures in PRC such as amphibians or primates, however, USH proteins, including harmonin, seem to co-localize with CP or, at least, appear in the transition zone of IS to OS (Sahly et al., 2012a; Schietroma et al, 2017). Very recently, we detected harmonin also in the OS of rods, in Müller glia cells (MGC) as well as in the outer limiting membrane of the human retina at the adhesive junctions between MGC and PRC (Nagel-Wolfrum et al., 2021)."*

We shortly refer to the same topic also in the results section, where we highlight our finding:

*"Previous studies of USH1C/harmonin expression, particularly in the mouse retina, were inconsistent but indicated localization of harmonin in PRC, MGC and secondary retinal neurons. (Reiners et al., 2003; Reiners & Wolfrum, 2006; Sahly et al, 2012b; Williams et al, 2009b). In WT pigs, we detected harmonin in the PRC layer, the outer limiting membrane (OLM) and outer plexiform layer (OPL) (Figure EV3). More specifically, protein was found in PRC outer segment (OS), the OS base, in CP and synaptic pedicles. Harmonin abundance in MGC microvilli tips and the cell adhesion region of the OLM is in line with expression profiles and subcellular localization of harmonin in the human retina (Nagel-Wolfrum et al., 2021)."*

We hope that the referee agrees that, according to the above mentioned arguments, our data are a major step forward towards a reliable intracellular localization of harmonin, at least in pigs, and that our description is scientifically conservative and, thus well qualified.

*14. Figure 4D. Please give a detailed protocol of how the analysis has been performed. How many replicates and animals underwent the analysis. This should be clearly indicated in the legend. The graph on the right shows no error bars. Was this measurement from one gel? I think it is very speculative to make a conclusion from n=1.*

→ We agree with the reviewer to add further details on our protocol for our analysis in Figure 4G / new Figure 5B. As indicated in the revised legend we now show a representative Western blot of the analysis of protein lysates obtained from WT and USH1C piglets and indicate that the quantification of Western blot bands was performed in an the LI-COR Odyssey infra-red system. The edited legend follows:

***"Figure 5. USH1$^{R31X}$ induce upregulation of GFAP and a synaptic phenotype in USH1C pig photoreceptor cells. (A, B)*** *Upregulation of GFAP in the retina of USH1C pigs at an age of 3 weeks (3w).* ***(A)*** *Representative immunofluorescence staining of GFAP in Müller glia cells which extend throughout almost the entire retina from the OLM (arrow) to the ganglion cell layer (GCL) of the retina. The consistent increase of GFAP expression in the Müller glia of USH1C pigs indicates Müller cell activation and gliosis. IS, inner segment; ONL, outer nuclear layer; OPL, outer plexiform layer; INL, inner nuclear layer; IPL, inner plexiform layer; scale bar: 20 µm.* ***(B) Left panel:*** *Western blot analysis of GFAP protein*

*expression in 3w old USH1C piglets and age matched controls. Anti-actin Western blot was used as loading control.* ***Right panel:*** *Quantification of Western blot bands in 4 gels by the LI-COR Odyssey system revealed a strong increase GFAP expression in 3w USH1C piglets when compared to age-matched WT controls (2 piglets, 3w, 2 retinas each, 2 TRs, error bars represent standard deviation (SD) of the mean, two-tailed Student's t tests, **p≤0.01).* ***(C, D)*** *Reduced synaptic width in USH1C pigs at an age of 1 year (1y).* ***(C)*** *Fluorescent microscopic analysis of the cone synapse phenotype.* ***Left panel:*** *Representative images of longitudinal sections through WT and USH1C pig retinae stained for the pre-synaptic marker PSD-95 (green) and by fluorescent peanut agglutinin (PNA, red) for cone synaptic pedicles (white arrows) and counter-stained by DAPI for nuclear DNA (blue). Scale bar 10µm.* ***Middle panel:*** *Higher magnification of a PNA-stained cone synaptic pedicle. Synapse width was determined as the maximum extension of consistent PNA signals. Scale bar 1µm.* ***Right panel:*** *Measuring cone synaptic pedicle width in WT and USH1C pigs by applying a Fiji script to PNA-stained sections indicated reduced synaptic width (2 pigs, 1y, 1 retina each, number of examined synapses indicated, error bars represent SD, two-tailed Student's t tests, ***p≤0.001).* ***(D)*** *Determining cone synaptic pedicles width by TEM.* ***Left panel****: Representative images of retinal cross sections. Scale bar 500nm.* ***Right panel:*** *Quantification of synaptic width confirmed the significantly reduced width of cone synaptic pedicles in USH1C pigs. (1 pig, 1y, 1 retina each, number of examined synapses indicated, error bars represent SD, two-tailed Student's t tests, *p≤0.05)."*

*15. Page 8. The authors used human USH1C splice variant a1 for rescue experiments. Did this splice variant is most abundant in the pig's retina? Would be nice to see rtPCR data from the pig's retina that support the choice of isoform for gene therapy.*

→ we followed the referee´s suggestions and extended out RT-PCR data set. In addition to the PCR on the 5´-end of USH1C transcripts that does not discriminate the _a, _b and _c splice variants and shows correct splicing of the humanized exon2 into USH1C transcripts, we designed 2 RT-PCR that should amplify the _a and _c variants as well as the _b3 variant (based on the GenBank designations). This PCR showed (i) that _b variants represent only a minor variant that was hardly amplified by 4 distinct primer sets in a number of distinct tissues. (ii) that no alternative splicing of exon11 that should characterize the _c splice variant appears in any PCR product. (iii) that instead alternative splicing of exon 13 in _a variants were observed in different tissues. (iv) that the retina indicated alternative splicing of exons 13 and 14 at very low level). (v) that _b variants consistently lack exon 27 and have exon 20 alternative spliced. We have to consider that we did not ultimately clarify the entire splice-spectrum of USH1C in different tissue nor in the retina, but it became clear that USH1C_a1 (including exons1-15, 21-26, 28) is the dominant variant in the retina. This matches with a recent study of ours, in which we have identified USH1C/harmonin_a1 as the most abundant splice variant in the human retina (Nagel-Wolfrum et al. 2021 doi:10.1101/2021.08.27.457962). The decision to choose the USH1C_a1 splice variant for gene therapy is also illustrated by its effect on dermal fibroblasts (Figure 7C) and in the new data on retinal gene therapy in vivo (Figures 7D and EV5). We integrated the major findings on common USH1C splice variants in Figures 4A-C and S9 as well as in Figure S3 where we now illustrate the identification of two distinct KO alleles in some of the founder animals.

*16. The whole section on testing therapeutic approaches, including Figure 6, should probably be removed. These tests are mostly not directly related to the animal model, and they are not related to each other. Save them for a paper that treats the pig model.*

→ we accept the referee´s point of view that treatment studies can be seen as an add-on to the core of the manuscript. Considering referee #1´s, referee #3´s and the editor´s opinion of this section as illustrating the translational potential of the model, we have, however, extended this section and show now also the efficacy of retinal gene therapy in vivo (Figures 7D and EV5, for details please see our

comments to referee #1 pt 5´s concern). We hope that referee 2 will agree that these new experiments have substantially improved the relevance of our manuscript.

Other comments:

*17. Page 2, "with at least 12 genes assigned to three clinical USH types". I would recommend change 12 to 10. There are five USH1 (myosin VIIa, harmonin, cadherin-23, protocadherin-15, and Sans), a putative USH1 (CIB2), three USH2 (usherin, ADGRV1, whirlin), and one USH3a (clarin-1) proteins (Géléoc G, El-Amraoui A, 2020). CIB2 is referred to as atypical USH as, unlike for all other 5 USH1 genes, several loss of function pathogenic variants in the CIB2 gene in both humans and mice were shown to cause the DFNB48 non-syndromic hearing loss, without loss of balance or vision (Booth et al., 2018; Michel et al., 2017).*

→ We thank the referee for pointing at this ambigous aspects of USH. We agree with the reviewer´s position, that 10 genes are commonly accepted as valid "USH genes" in the USH field. In our previous calculation, we have added CIB2 still as USH1J (Riazuddin et al. 2012, doi:10.1038/ng.2426) and ESPIN as USH1M (Ahmed et al. 2017 DOI: 10.1136/jmedgenet-2017-105221), which are still debated. CIB2 for sure is a deafness gene, most probably non-syndromic because recent studies did not confirm a loss of balance or vision (Booth et al., 2018; Michel et al., 2017) and that ESPIN was only named as an USH gene in one publication. Interestingly, it has been shown that in a more recent study Cib2 deficient mice develop retinal degeneration and vison loss (Sethna et al. 2021 doi: 10.1038/s41467-021-24056-1), which certainly inspires the debate. Similar to CIB2, some mutations in USH1C (R608P, IVS12DS G-C+5, according OMIM #605242), MYO7A (D218N, G671S, R395H, N458I, M599I, R244P etc. according OMIM #276903), CDH23 (D1243N, D1400N, D2148N, D1348N, P1888S, etc. according OMIM#605516) or PCDH15 (G262D, R134G, V528D, according OMIM #605514) do only cause deafness. Conversely, some USH2A mutation (C759F, R4674G, according OMIM #608400) are classified as non-syndromic RP, whereas most mutations in these genes constitute "typical" syndromic Usher, i.e. combined blind-deafness. In some cases, the reason might be that mutations appear only in splice variants that are indispensable in one organ, but not in the other (e. g the USH1C mutation are localized in or around the CC2 and PST domains of harmonin that is expressed in the ear, but not in the eye). In other cases, the reason for the distinct phenotype is less clear. Most likely some mutations retain some residual activity that is sufficient to retain the function in one organ but not in the other. For USH1E there is no causative gene defined. A further ultra-rare USH2 variant has been vaguely localized to chr15q. It is, however, beyond our manuscript to cover these partially unresolved peculiarities of USH gene and we agree with the referee´s recommendation that "*at least 10 genes assigned to …*" is the most consistent and conservative statement and changed the sentence accordingly.

*18. Page 2, "The most severe of them is USH1, characterized by profound hearing loss from birth on, vestibular areflexia, and pre-pubertal onset of Retinitis pigmentosa (RP)". Change "Retinitis pigmentosa" to retinitis pigmentosa.*

→ We followed the referees recommendation and changed the wording accordingly.

*19. Page 3 "extended CTCF/cohesin-binding","3 intronic SNPs identified on the disease-allele". It is usually recommended to introduce every acronym before using it alone in the text.*

→ We followed the referee´s suggestion and emphasize that CTCF abbreviates "CCCTC-binding factor". As this very specific genetic term might not completely reach each readers satisfaction, we provided two references of recent review articles (Ghirlando & Felsenfeld, doi: 0.1101/gad.277863.116; Holwerda & deLaat doi:10.1098/rstb.2012.0369) that nicely and very globally describe the relevance on CTCF elements for gene regulation and illustrate why we put emphasis on such elements in our bioinformatics analysis. We also explained that SNPs abbreviates "single nucleotide polymorphisms" in the same paragraph.

*20. Figure 1. Incorporate into Figure 1D the list of examined species to the right or left of the sequence*

*data. Rephrase the 1D legend since it is not accurate. The gel on Figure1H represents the excision of the selection cassette. Specify in the legend the meaning of the grey boxes. What template was used as a control in this experiment?*

→ In accordance with pt5, we have restructured Figure 1 and moved parts into Figure EV1 for better visualization. We have incorporated the species names for Figure 1D and specified the Figure caption: *"**(D)** At protein level, all examined species show identical amino acid sequence and high degree of conservation at the intronic borders at nucleotide level."*. Former Figure 1H is now in Figure EV1. The grey boxes in the gel picture indicated effective excision of the selection cassette after lipofection with Cre-mRNA. Realizing that it more confused the readers than it supported understanding, we have removed these boxes and described the outcome in the figure text. The Controls (Ctrl) were artificially created by mixing genomic WT DNA with modified BAC vector (for the neo PCR) and with modified BAC after treatment with Cre (for the delta-neo PCR). We have clarified these issues in the Figure text which is now *"(G) Representative genotyping of a founder litter, indacting that animals 5313 and 5317 have sufficiently excised neo. For controls (Ctrl.), WT genomic DNA was mixed with modified BAC for the neo-PCR (yellow) and mixed with a modified BAC that has been treated with Cre for the delta-neo PCR (blue)."*.

*21. Page 4 "(Figure EV1A-F)" Is this an independent sentence? There is no explanation in the text relevant to the Figure.*

→ we apologize for the typo and corrected the error.

*22. Page 4 "At an age of 3-6 months, we occasionally observed nystagmus (Movie 2), a common phenomenon of vestibular dysfunction and vertigo (Dougherty et al, 2020) and previously described in USH patients (Bonneau et al, 1993; Pieke-Dahl et al, 2000; Puffenberger et al, 2012)". The cited references (Pieke-Dahl et al., 2000; Puffenberger et al., 2012) do not accurately represent the statement in the text; I would recommend replacing them with more suitable references. Tiny point: "et alia" is abbreviated in citations as "et al." with a period. Even though the abbreviation is rather silly as it only saves one character, we have to do it correctly.*

→ This point refers to reviewer #1 pt2. We have discussed this aspect with our new co-authors Drs. Straka and Dlugaiczyk (experts on the vestibular and auditory system) and have corrected the citations and put the aspect of nystagmus in a broader context. The respective section reads now: *"At an age of 3-6 months, we occasionally observed a spontaneous nystagmus of the eyes (Movie 2) in some animals. This is very likely caused by caused by an asymmetrical tonic firing of vestibular afferents from the right and left labyrinth (Baloh et al, 2012; Dougherty et al, 2020), and has been anecdotally described in USH patients (Bonneau et al, 1993; Moller et al., 1989)."*

Regarding the citation style we are as puzzled as the referee as we used the EMBO Mol Med citation style of the End-Note reference program for preparing the citation list of the initially submitted manuscript. We have now updated the EMBO Mol Med citation formula at EndNote.

*23. Page 4 and Figure 2A. Indicate in the text or the legend the age and number of animals that were tested, the frequency of the test for each animal.*

→ This comment refers to referee #1 pt2. We describe the constellation of examined groups and the number of test performed in the dark and under light conditions in the figure text. As pointed out at in our answer to pt.6, we did not feel capable of performing serious statistical tests for significance for the barrier test (Figures 2A, S4) as we examined uneven und small groups of animals there.

*24. Figure legend 2. "Impaired movement of USH pigs in 3-dimensional space". The title of the figure is not accurate. The legend also has references to non-existent figures such as "see Figure S2, Figure S3, S4".*

→ We apologize for the inconvenience. The new title of Figure 2 is *"Impaired orientation of USH1C pigs in 3 dimensional space"*. The reference to supplemental data is corrected (Figures S4 – S6).

*25. Figure legend 2 "Mean values {plus minus} standard deviation are given by lines, mean values {plus minus} standard error are given by numbers, p-values for Mann-Whitney U-tests are indicated. Analysis included all runs, for normalized data sets, see Figure S4". Is this standard deviation or standard error? Please correct*

→ We apologize for the insufficient specification. We have now indicated in the figure text of (now Figure S6) illustrates mean values plus/minus standard error.

*26. FigureEV5 C. What genotype/age represents the figure?*

→ we apologize for the misleading description. Former Figure EV5 is now EV2. (C) is an 8 week old USH1C animal, (D) are 2 years old USH1C and WT animals. We have described it more clearly now in the figure caption: *"(C) and (D) show exemplary response curves for determining sound pressure threshold levels in an 8-week-old USH1C animal (C) as well as 2-year-old USH1C and WT animals (D)."*

*27. Page 6 "Interestingly, spectral domain optical coherence tomography (Figure 3E) revealed only minor differences in retinal architecture between WT and USH1C pigs with good preservation of outer retinal layers containing the PRCs, suggesting a large therapeutic window". What age was tested? How many animals? It is good to see average thickness across multiple scans in both USH1C and WT*

→ We specified this aspect now; two animals were examined for each genotype at an age of 12 months. We decided to show only representative examples as we did not find substantial or significant differences. We did not fully get the idea of the referee´s argument for measuring multiple scans since this suggests a verified non-significant difference, which in fact would not be the case.

*28. Figure 4C. There is some signal present in the panel with harmonin staining. Is this a real signal or background? It would be good to see co-staining with nuclei markers and photoreceptor markers to better see architecture of the retina and evaluate the quality of the sections.*

→ We followed the reviewer´s suggestions and added the co-staining with DAPI as a nuclei marker in the new Figure 4E to visualize the retina layers.

*29. Figure EV 6A the outer limiting membrane (arrow). There is no arrow on the figure.*

→ We apologize for the inconvenience and added the arrow into the image (new Figure EV3A).

*30. Page 7. "While the structure and arrangement of PRCs appeared grossly intact in USH1C pigs (Figures 4e, S7a), subcellular analysis revealed specific disruptions in the architecture of compartments comprising harmonin." This statement is very speculative. No data that support this statement. Also, Figure S7a is not found. Probably "containing" is more appropriate than "comprising."*

→ We apologize for the ambiguity and have improved the wording and refer to the correct figures now (including also the cSLO of 1 year old USH1C and WT pigs (requested in pt12) in new Figure S8) for which reason we now refer to the structure and arrangement of the retina rather than PRC. The modified sentence reads now: *"While the general structure of the retina appeared grossly intact in USH1C pigs (Figures 4E, EV3G, S8), subcellular analysis revealed specific disruptions in the architecture of compartments containing harmonin."*

*31. Figure 5 C, E and the relevant legend is incomplete. Was mean presented with SD or SE? It is hard to believe just looking into the graph that the difference is significant. The data has to be reanalyzed and properly presented. The same problem is with FigureEV7 B and C (right panel). Authors also should provide the detailed protocol of quantitative fluorescent microscopic analysis and transmission*

*microscopic analyses that they performed, otherwise, it is not possible to evaluate the methodology.*
→ This criticism has a very valid point. In the revised version, we describe the respective statistical tests and the p values for Figures 2, 5-7 in the respective figure captions. More detailed protocols for our quantitative fluorescent microscopic analysis of connecting ciliary length and cone synaptic width in retinal sections by using Fiji scripts are provided now in Supplemental Materials and Methods (page 3). Further, we clarify in Supplemental Materials and Methods that *"Measurements of structures such as connecting cilia length or synapse width were performed directly in the TEM using the measuring tool of the Olympus SIS image analysis system (Soft Imaging Systems, Münster, Germany)."* (page 4).

*32. Figure EV7 C. Would it be possible that the cone synaptic pedicles is being wider is an artifact (if the sectioning was more tangentially oriented then they could appear longer when they are not) ?*
→ For avoiding artifacts, we have analyzed the width in cone synaptic pedicles by light/fluorescence microscopy in longitudinal cryosections through pig retinae (new Figure 5C), but also determined the length of connecting cilia in PRC (Figure 6 B, C) in addition to the direct TEM measurements. For both, the synaptic pedicle width and the connecting cilia length, the direct examination of TEM the indirect staining in immunohistology yield the same results. Thus, we feel rather confident to claim a reduced width of cone synaptic pedicles and elongated connecting cilia in PRC. For detailed argumentation please see comments to referee #1 pt4.

*33. Page 7. Specify in the text or in the legend the dose/volume that was injected for each vector. Were samples immunostained to amplify GFP signal? It is always beneficial to co-stain with DAPI and other markers to evaluate retina architecture.*
→ We added the requested information on the virus amount (200 µl of each AAV ($2*10^{10}$ AAV particle)) in the text and provide a co-staining of GFP (direct signal, no anti-GFP immunostaining) and DAPI (Figure 7A).

**Referee #3 (Remarks for Author):**
*Review of the manuscript "Early Disruption of Photoreceptor Cell Architecture and Loss of Vision in a Humanized Pig Model of Usher Syndrome" by Dr. Klymiuk and colleagues [EMM-2021-14817] General comments:*
*The authors generated a pig model for Usher Sydrome type 1C by introducing a human mutation into the harmonin-encoding gene. The study describes in detail the phenotype of the USH1C pigs that reflects quite well the syndromic disease of human patients: hearing defects, vestibular dysfunction and visual impairment. The model and the results are convincing and well described. However, while this USH1C pig model is certainly very interesting for future pre-clinical studies, I don't think that the data so far presented in this study have substantially improved our molecular understanding of the USH1C disease as claimed in the last paragraph of the Discussion. The study is essentially based on visual and motion behavior. The therapeutic approaches described in the manuscript either with AAV strategy or CRISPR Cas9 technology also appear to have been treated rather superficially in cell culture and require further in-depth investigation in vivo.*
→ we have incorporated the concerns of all referees on this section and have substantially improved this part by adding data on therapeutic AAV treatment in vivo. For details see please our answer to referee #1 pt5.

Specific comments:
*1) A single allele insertion of the human mutation gave a progressive "dominant-negative" phenotype in Heterozygote pigs as indicated by the age-dependent increase of the hearing thresholds (Figs3A and*

*EV5). Could you please discuss this point in regards to the human disease and to the development of future genetic therapy.*

→ we agree that a progressive "dominant-negative" effect of heterozygous USH1C contradicts to the definition, or better said, common assumption of an autosomal recessive disease that should theoretically not show any clinical phenotype in its heterozygous status. In the meanwhile, however, there is some shift in the opinion on this assumption. In general, it is simply a matter of how detailed a phenotype is described. In many cases, the findings might be biologically irrelevant. In some cases, a heterozygous constellation might even be beneficial under certain circumstances and propagate the allele frequency in a population. A most popular example is sickle cell anemia, caused by mutations in the HBB gene, providing some protection from malaria in a heterozygous stage (Piel et al. Nat Commun 2010 DOI: 10.1038/ncomms1104), leading to enrichment of the diseased allele in areas with high malaria prevalence and an increased appearance of beta-thalassemia in the respective human population; a similar beneficial effect of heterozygous mutations was presumed for the CFTR F508del allele in Caucasians due to protection against cholera (Gabriel et al. Science 1994 doi: 10.1126/science.7524148.), typhic fever (Pier et al. Nature 1998 doi:10.1038/30006), cattle pastoralism (Alfonso-Sanchez et al. Med Hypothes 2010 doi:10.1016/j.mehy.2009.12.018), bronchiolar asthma (Schroeder et al. Nat Med 1995 doi:10.1038/nm0795-703) or due to respiratory advantages during the last glacial period (Kurbel, Med Hypothes 2019 doi: 10.1016/j.mehy.2018.11.006). Given the extremely low frequencies of USH1C mutations in the human population, such a beneficial effect can be excluded and their appearance in patients is, very likely, a stochastic effect and not linked to any evolutionary selection. We have to point out that the reduced hearing effect in pigs is relatively small and that we are not sure if the observed effects can be verified in human USH1C carriers, due to the very small number of USH1C carriers and the divergent social and environmental conditions under which they live. We are also not aware of any reports on a reduced ability for hearing in USH1C carriers. We believe, however, that our finding will stimulate further research into that direction and clarify open questions. Such effects can be much better studied in animal experiments as cohorts can be kept under standardized environmental conditions. Whether this will result in a systematic investigations of USH1C carriers by hearing aids or any other treatment cannot be seriously estimated at present. We considered these points as interesting, but also highly speculative. We appreciate, however, referee 2´s interest in the topic and have added a short paragraph to the discussion section: *"In our work we give first indication for a potentially impaired hearing ability in heterozygous USH1C carriers (Figures 3A and EV2). As the results were consistent at distinct ages, in different groups of animals and across the examined frequencies, we interpret this as a slight but significant biological effect. It must be considered that we are not aware of reports on impaired hearing in human USH1C carriers and so we cannot seriously estimate if these findings are of clinical relevance for human USH1C carriers. Regarding the very low numbers of patients carrying distinct USH1C mutations, however, we would definitely exclude an evolutionary benefit of heterozygous USH1C defects, as it has been reasoned for the spreading of genes such as HBB (Piel et al, 2010) or CFTR (Gabriel et al, 1994; Pier et al, 1998) in the human population."*

*2) It would have been interesting to plot and compare the amplitude of the ABR waves I (hair cell synapses) and V (central synapses) as a function of sound intensity to determine whether the phenotype affects all sound intensities, in particular in the Hetero at various age. Indeed, since genetic deletion of harmonin has been shown to also affect the hair cell ribbon synapse in mice, analysis of the ABR wave amplitudes could have been informative regarding a possible progressive hair cell synaptopathy in pigs. Also, recordings of the DPOAEs, reflecting the mechanical amplification by the OHCs could have been interesting in order to get a more complete assessment of the auditory function.*

→ Thank you for pointing this out. It is a greatly appreciated suggestion to include DPOAE in the analysis for hearing assessment and OHC function in USH1C. When working with pigs, however, we

realized that DPOAE raise a number of technical challenges and require exact adaptation of probes and stimulus paradigms, tailored to the specific anatomical situation of the pig ear. We apologize that we cannot sufficiently address this question at present and would rather refer to future studies in which we plan to examine DPOAEs on USH1C pigs. As pointed out in our answer to referee #2 pt 9, examination of heterozygous USH1C animals is indeed an interesting undertaking. The small difference between the two genotypes, however, will require comprehensive studies under highly standardized examination, at best in a longitudinal manner, finally clarify this question.

*3) Throughout the text and figures, it is often difficult to know the exact number of animal studied. Please give the N number in the text for each test - such as for those described page 6 and Fig3.*
→ We recognized this shortage. We have followed the referee´s request and provide numbers of animals, eyes or technical replicates in all figure captions. For details see answers to the respective comments for referees #1 and #2.

*4) Page 8: Citing Nagel-Wolfrum et al. as unpublished data is insufficient, please give a more detailed description of AAV vector encoding USH1C variant a1 and the reasons of choosing this variant.*
→ We have performed RT-PCR that discriminates USH1C_a and USH1C_b variants, detects some alternative splicing variants, but identified USH1C_a1 as the major transcript variant in the pig retina. This is in line with data on human retina (Nagel-Wolfrum et al. 2021, doi:10.1101/2021.08.27.457962) and correlates also with the correction of the phenotypes in dermal fibroblasts in vitro and in the pig eye in vivo. For details, please see answer to referee #2 pt15.

*5) Give a more precise description of how the length of the primary cilium in primary skin fibroblasts was determined ? The use of this single parameter as a marker of restoration after gene therapy seems not convincing.*
→ We have now described the measurements of primary cilia in more detail in Supplemental Material and Methods (page 4). We added the number of ciliated cells as an additional readout parameter and second marker of restoration after gene therapy (Figure 7C).

*Minor points:*
*6) Page 6, in the text top paragraph, give the SD value of the a-wave amplitude with a decimal 10{plus minus}0.X?*
→ we have now indicated that the respective value is 10±0.1 µV.

*7) page 5, second para, replace "sensoneural" by "sensorineural"*
→ we thank the referee for the advice and corrected the term accordingly.

16th Dec 2021

Dear Dr. Klymiuk,

Thank you for the submission of your revised manuscript to EMBO Molecular Medicine. We have now received feedback from the three referees who re-reviewed your manuscript. As you will see from the reports below, they are now overall supportive of publications, however referee #1 still raises some issues that should be addressed before acceptance of the manuscript. Therefore, we would like you to address the comments raised by this referee, as well as the minor comments from referee #3.

Additionally, please also address the following editorial issues:
- Please provide up to 5 keywords.
- Please remove "Data not shown". As per our guidelines, on "Unpublished Data" the journal does not permit citation of "Data not shown". All data referred to in the paper should be displayed in the main or Expanded View figures.
- As we do not have size limitations, please consider inserting (part of) your supplemental Mat & Met in the main manuscript text.
- Please remove the Movie legends from the Appendix and from the Appendix table of content.
- Please update nomenclature of the movies to Movie EV1 etc. Please zip movie file together with corresponding legend in txt/docx format.
- Thank you for providing a nice synopsis picture. Please also provide a synopsis text. Synopses include a short stand first (maximum of 300 characters, including space) as well as 2-5 one-sentences bullet points that summarizes the paper. Please write the bullet points to summarize the key NEW findings. They should be designed to be complementary to the abstract - i.e. not repeat the same text (maximum of 30 words / bullet point). Please use the passive voice.
- As part of the EMBO Publications transparent editorial process initiative (see our Editorial at http://embomolmed.embopress.org/content/2/9/329), EMBO Molecular Medicine will publish online a Review Process File (RPF) to accompany accepted manuscripts.
This file will be published in conjunction with your paper and will include the anonymous referee reports, your point-by-point response and all pertinent correspondence relating to the manuscript. Let us know whether you agree with the publication of the RPF and as here, if you want to remove or not any figures from it prior to publication.
Please note that the Authors checklist will be published at the end of the RPF.

I look forward to receiving your revised manuscript as soon as possible. Use this link to login to the manuscript system and submit your revision: in ot A i e

With kind regards,

Lise Roth

Lise Roth, PhD
Editor
EMBO Molecular Medicine

***** Reviewer's comments *****

Referee #1 (Remarks for Author):

The current version of the manuscript appears significantly improved. An impressive number of data has been overall collected, allowing a very comprehensive characterization of the pig model. Also, the new exploratory data on the use of this USH1C model for investigating the in vivo efficacy of therapeutic approaches appear promising. However, there are several aspects that, in this reviewer opinion, still require improvements:

- The paragraph on evaluation of therapeutic efficacy should be expanded. Indeed, while the use of only two animals appears reasonable due to the difficulties related to large animal studies, all data collected should be shown for both animals. Given the large number of consistent phenotypic readouts described from the authors in the USH1C pigs (i.e. cilia length, OS morphology, mfERG, etc...), it is unclear why these parameters were not evaluated in the AAV- vs PBS-treated eyes. Inclusion of these data appears relevant especially considering that the whole characterization of the animal model shown in Figures from 1 to 6 is based on animals of up to 1 year of age, while the animals used for evaluation of therapeutic approaches were significantly older (2-3 years of age). Can the author comment if they see any further exacerbation of the disease (i.e. by evaluating cilia

length, OS stacks, ONL thickness...) at these later ages?

- In the first paragraph, the authors mention they found two different types of mutation in the founder animals. However, it is then unclear the genotype of the animals included in all the following studies. Please clarify. If pigs with different genotypes were used across the studies, it is recommended to include in the manuscript a table summarizing the genotype of the animals used in each experiment/figure panel.

- Fig. 2: for those tests where an adequate number of animals were available (as in panel B-C, where 5 animals per group were used), it would be more explicative to see graphs in which each dot represents the average performance of each animal, rather than each of the runs. This data could be added in the Supplementary Material section. Also, when data from animals which differ in age are pulled together (as in panel A where 5- and 12-months old pigs are used), it would be interesting to know how comparable data from these ages are and if any trend in worsening of the disease is seen.

In addition, the following minor modifications are suggested:

- Page 11: "the success of mRNA therapy in exploratory experiments in vivo..." is somehow misleading as the in vivo gene therapy approach used by the authors is based on delivery of a gene rather than an mRNA. It appears more appropriate to simply refer to it as "gene therapy" or "AAV-based delivery of harmonin"

- Legend to Fig.7 and Supplemental Materials and Methods section (page 7): please use "distribution in retinal layers" rather than "biodistribution", which usually refers to distribution in a whole body. As a side comment, given the use of the ubiquitous CMV promoter, it would be better to show in Fig.7 pictures where expression in the RPE cell layer is visible too.

- Legend to Fig. S1: It appears that a string of the legend, which included the last part of the description of panel F as well as the beginning of the description of panel G, is missing. Please check.

- Page 8: it appears that the "Fig. 8SD, E" is erroneously referenced after the sentence "USH1C_b splice variants were found at low level in general, but clearly indicated a lack of exon 27 in all transcripts and alternative splicing of exon 20 (Figure 8SD, E)." Please check.

Referee #2 (Remarks for Author):

I think this is a nice study and the paper is much improved now. The revised manuscript has adequately addressed my concerns. I think it is suitable for publication.

Referee #3 (Remarks for Author):

Manuscript Number: EMM-2021-14817-V2

Title: Early Disruption of Photoreceptor Cell Architecture and Loss of Vision in a Humanized Pig Model of Usher Syndrome
Corresponding Author: Dr. Klymiuk

In this revised version, the authors have significantly improved the quality of the manuscript and have satisfactorily responded to my points. The authors now provide new data generated by sub-retinal injection of AAV-harmonin_a1 vectors that show significant restoration of retinal function in two USH1C pigs (Fig 7D and Fig EV5). Overall, the revised manuscript shows more convincingly a translational clinical potential of their USH1C pig model.

I only have few minor comments:

1) The last sentence of the abstract is not clear and needs to be rewritten. Please change mRNA therapy that could be confusing by AAV-gene therapy or gene therapy.

2) I think it is important to precise somewhere in page 10 of Results that the modification of the porcine USH1C gene in exon 2 (Fig 1C) will affect the three isoforms of harmonin.

3) Problem: Fig 7D left graph is identical to Fig EV5D. In Fig EV5D, I suggest to select another ERG recording from pig # 10056. Alternatively, I suggest to replace the right panel 7D by the two panels of EV5B. Also indicate the numbering of the 2 pigs # 10056 and # 10054 in 7D . In EV5 then show only panels A and C.

4) Page 21, Legend of Fig7A last sentence, (representative images from 1 pig - remove s)

**16th Dec 2021**

Dear Dr. Klymiuk,
Thank you for the submission of your revised manuscript to EMBO Molecular Medicine. We have now received feedback from the three referees who re-reviewed your manuscript. As you will see from the reports below, they are now overall supportive of publications, however referee #1 still raises some issues that should be addressed before acceptance of the manuscript.
Therefore, we would like you to address the comments raised by this referee, as well as the minor comments from referee #3.
→ we thank the editor and referees for kindly and patiently supporting our work. We appreciate their judgement and have made modifications on the manuscript version that was finally accomplished by EMBO Mol Med´s editorial team and us on Nov.26th. Any sections that have been marked in red as modifications within the revised Nov.-version have now turned black as a sign of acceptance. Any modifications that have newly been made in this final step are marked in orange for valid identification.
In addition to minor modifications in the text, some minor shifting of figures components and the implementation of Materials & Methods in the main text document, the referees´ requests demanded in the implementation of 3 new supplementary figures: Figure S7, the individual variability of pigs in the behavior tests, as requested by referee #1, pt 3. Figure S10, the illustration of AAV-transduction into RPE, as requested by referee #1, pt 5. Table S1, a correlation of experimental animals to their genetic status in the USH1C gene and the genetic background, as suggested by referee #1 pt 2.
We have also made slight modifications on the gene editing section (Figures 7B and EV4). We now exclusively show data on primary cells that yield the humanized R31X segment on one allele and a larger deletion, including porcine exon 2 on the second allele. This is consistent with most of the USH1C pigs that we produced by breeding and, even more relevant, to the situation in USH1C patients. There, the extremely low frequency of mutations lead to compound heterozygosity, meaning that a R31X allele is always accompanied by another USH1C-disrupting allele. Although our initial examination of mono- and bi-allelic modifications of the humanized fragment is interesting from a basic scientific point of view, internal discussions and the suggestions by referees during the review process stimulated a slight adaptation of data presentation: Experiments on mixed cell clones have been initially done with cells carrying the R31X/del allelic constellation and therefore remain as they were. Examination of single cell clones is now shown for 68 SSC generated from the same cell population, carrying the R31X/del allelic constellation. The new depiction does not affect the data in quantitative manner, but the respective paragraph in the results section and the legends of Figures 7B lower right, and EV4D were slightly adapted to improve readability, understanding and translational interpretation of our work.

Additionally, please also address the following editorial issues:
- Please provide up to 5 keywords.
→5 keywords are provided: gene therapy / impaired vision / photoreceptor morphology / pig model / Usher Syndrome /

- Please remove "Data not shown". As per our guidelines, on "Unpublished Data" the journal does not permit citation of "Data not shown". All data referred to in the paper should be displayed in the main or Expanded View figures.
→we double checked the manuscript and did not find the any remaining "data not shown" in the final text. We might have, however, caused confusion, by marking deleted sections by red, strike-through text passages. We have now completely removed this sections from the text.

- As we do not have size limitations, please consider inserting (part of) your supplemental Mat & Met in the main manuscript text.
→ we appreciate EMBO Mol Med´s ambition at documenting methods in the main text section. We found it, however, a bit confusing to move only parts of the supplementary Materials & Methods section, because it might compromise readability, if one has to switch permanently from the main text to the supplementary document. We propose to either keep the composition as it was in the revised version, with a short reference in the main text to the extended materials and methods in the supplementary document or include the comprehensive description of the methods in the main text, as we present in our new version. In the latter case, it would make most sense to keep only the antibody and gene synthesis references in the supplementary text. Without doubt, we would be happy with the latter option, but due to the complex and diverse methods we used, this would significantly increase the volume of the main text (>5000 words and approx. 20 new references). For this reason, we kindly ask the editor for a decision on the final structure of the manuscript.

- Please remove the Movie legends from the Appendix and from the Appendix table of content.
→ we followed the editor´s suggestion.

- Please update nomenclature of the movies to Movie EV1 etc. Please zip movie file together with corresponding legend in txt/docx format.
→ we updated nomenclature and renamed movie files Movie EV1 – Movie EV7. We also set up a common .docx file describing the content of all movies. EMBO Mol Med, however, did not faciliate uploading .zip files and allowed only .mp4, .mov or other movie formats. We hope that providing separate movie files in .mp4 format with a common description in a .docx is convenient.

- Thank you for providing a nice synopsis picture. Please also provide a synopsis text. Synopses include a short stand first (maximum of 300 characters, including space) as well as 2-5 one-sentences bullet points that summarizes the paper. Please write the bullet points to summarize the key NEW findings. They should be designed to be complementary to the abstract - i.e. not repeat the same text (maximum of 30 words / bullet point). Please use the passive voice.
→ a blurb text and 5 bullet points have been included in to the manuscript text.

- As part of the EMBO Publications transparent editorial process initiative (see our Editorial at http://embomolmed.embopress.org/content/2/9/329), EMBO Molecular Medicine will publish online a Review Process File (RPF) to accompany accepted manuscripts. This file will be published in conjunction with your paper and will include the anonymous referee reports, your point-by-point response and all pertinent correspondence relating to the manuscript. Let us know whether you agree with the publication of the RPF and as here, if you want to remove or not any figures from it prior to publication. Please note that the Authors checklist will be published at the end of the RPF.
→ we agree on publishing a corresponding RPF, including author checklist with our manuscript. We are not completely sure which figures are meant to be removed or not.

I look forward to receiving your revised manuscript as soon as possible. Use this link to login to the manuscript system and submit your revision: https://embomolmed.msubmit.net/cgi-bin/main.plex

******* Reviewer's comments *******

**Referee #1 (Remarks for Author):**

The current version of the manuscript appears significantly improved. An impressive number of data has been overall collected, allowing a very comprehensive characterization of the pig model. Also,

the new exploratory data on the use of this USH1C model for investigating the in vivo efficacy of therapeutic approaches appear promising. However, there are several aspects that, in this reviewer opinion, still require improvements:

1) The paragraph on evaluation of therapeutic efficacy should be expanded. Indeed, while the use of only two animals appears reasonable due to the difficulties related to large animal studies, all data collected should be shown for both animals. Given the large number of consistent phenotypic readouts described from the authors in the USH1C pigs (i.e. cilia length, OS morphology, mfERG, etc...), it is unclear why these parameters were not evaluated in the AAV- vs PBS-treated eyes. Inclusion of these data appears relevant especially considering that the whole characterization of the animal model shown in Figures from 1 to 6 is based on animals of up to 1 year of age, while the animals used for evaluation of therapeutic approaches were significantly older (2-3 years of age). Can the author comment if they see any further exacerbation of the disease (i.e. by evaluating cilia length, OS stacks, ONL thickness...) at these later ages?

→ We agree with the referee that a consistent and comprehensive evaluation of defined cohorts of USH1C pigs and WT littermate controls would be desirable for profound examination of gene therapy efficacy, ideally within the first 12 months of age. Due to the limited numbers of animals available and the still open questions on the design of such pre-clinical studies (time point of intervention, dosage, follow-up period, etc.), however, we opted for initial pilot experiments. We used two retired breeders for exploratory pilot studies that would have otherwise sacrificed without further gain of knowledge. At the present time point, we have completed one experiment (#10056, injection at 2.5y), due to the COVID restrictions, however, without intermediate examinations. The experiments will not be fully comparable to gene therapy in another animal (#10054, injection at 3.5y). Importantly, however, we clearly document harmonin abundance in the treated eye by molecular and morphological examination. In line with the distribution studies with eGFP (Figure 7A and new S11, see referee #1, pt 5), we show that distinct cell types can be addressed by AAV. Both therapy experiments indicate that harmonin reconstitution leads to (partial) correction of ERG levels in rods and cones, suggesting that human-relevant therapies can be sufficiently examined in USH1C pigs. We think this as a major achievement and have now included the light-adapted ERG data from both animals in Figure 7D (as suggested by referee #3, pt 3) while the data on dark adaptation and the summary of quantitative values are given in Figure EV5. It is clear that at present we neither can seriously answer questions on appropriate virus dosages, injection time points, sustainability of treatment, etc., nor truly estimate the ultimate potential of AAV-based gene therapy for USH1C. We are, however, convinced that depicting these pilot experiments is highly relevant for the comprehensive characterization of the USH1C pig model and we hope that the chosen strategy will satisfy the editor´s, referees´ and readers´ interest.

2) In the first paragraph, the authors mention they found two different types of mutation in the founder animals. However, it is then unclear the genotype of the animals included in all the following studies. Please clarify. If pigs with different genotypes were used across the studies, it is recommended to include in the manuscript a table summarizing the genotype of the animals used in each experiment/figure panel.

→ we agree with the referee that an overview on the animals used in the distinct experiments would be helpful for the readers. We have added a supplementary “Table S1. Experimental animals” to conclusively demonstrate the usage of the respective animals and their genotype and refer to this new data set in the results section.

3) Fig. 2: for those tests where an adequate number of animals were available (as in panel B-C, where 5 animals per group were used), it would be more explicative to see graphs in which each dot

represents the average performance of each animal, rather than each of the runs. This data could be added in the Supplementary Material section. Also, when data from animals which differ in age are pulled together (as in panel A where 5- and 12-months old pigs are used), it would be interesting to know how comparable data from these ages are and if any trend in worsening of the disease is seen.
→ We thank the referee for this great suggestion. We have indeed performed these types of analysis and found support for our conclusion that USH1C pigs were consistently slower and used snout contacts more often than WT control pigs. There was no indication that single individuals shifted the total data points towards significant differences. We found it stimulating to implement the individual data sets in a new Figures S7 and stated that *"Plotting the data points for individual animals supported the interpretation of a general difference between USH1C pigs and WT control animals (Figure S7)."* in the results section. The analysis, however, also showed the limitations of splitting data sets into individual subsets, in particular when we worked with smaller data sets such as the experiments in the dark. The issue of reduced resolution is even more pronounced when we looked at the potential progression of vision loss. We have accumulated data from the 3 consecutive initial runs (runs conducted within 2-3 weeks, animals approx. 7 months old) and 3 consecutive runs from later stages (animals approx. 10 months). Even the shift of one run in the analysis (i.e. exploration of runs 15-17 vs. runs 16-18) yielded different results.

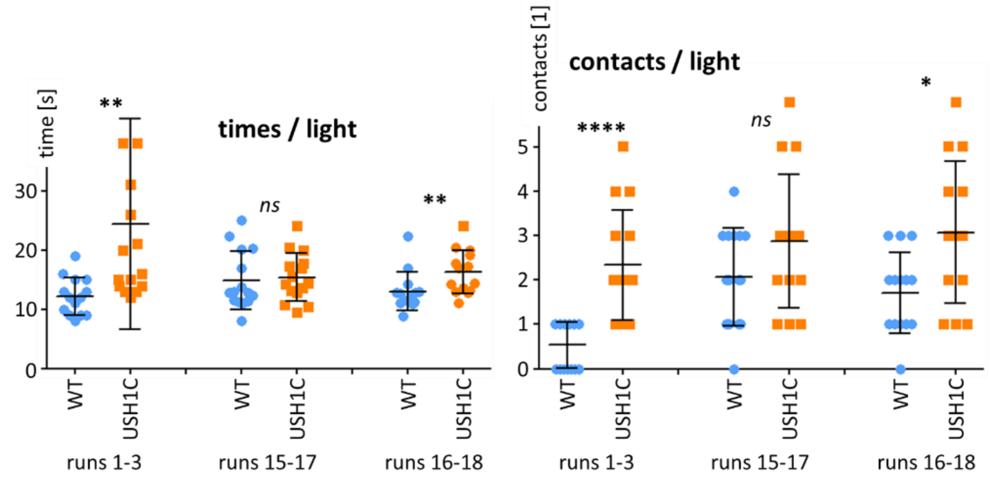


This confirms our assumption that the varying composition of the respective courses (see Figure S5) that was necessary to prevent a learning effect of the animals had a significant impact on the performance. Further, the compliance of the animals on the experimental days was variable. Specifically, WT pigs seem to lose interest in the test with increasing age, as indicated by increased snout contacts. For this reason, we would refrain from interpreting the increasing snout contacts of USH1C pigs as an indication of progressive vision loss. Generally, we think that the overall number of data points is simply too small to seriously estimate the progression of vision loss by this method. To avoid misinterpretation, we would therefore hesitate to include the graphs shown here for "early" and "late" experiments in a similar manner as we did for the individual performance in the new Figure S7.

In addition, the following minor modifications are suggested:
4) Page 11: "the success of mRNA therapy in exploratory experiments in vivo..." is somehow misleading as the in vivo gene therapy approach used by the authors is based on delivery of a gene rather than an mRNA. It appears more appropriate to simply refer to it as "gene therapy" or "AAV-based delivery of harmonin"
→ we agree that terms in scientific manuscripts are often used in a very casual manner and we recognize that we are susceptible for such incorrectness as well. We thank the referee for the

suggestions and use the super-precise and clear term “AAV-based delivery of harmonin” in the manuscript.

5) Legend to Fig.7 and Supplemental Materials and Methods section (page 7): please use "distribution in retinal layers" rather than "biodistribution", which usually refers to distribution in a whole body. As a side comment, given the use of the ubiquitous CMV promoter, it would be better to show in Fig.7 pictures where expression in the RPE cell layer is visible too.
→ We again ask for apologies for the superficial usage of terms. We use now the phrase “AAV transduction of retinal layers” to better describe that we aimed at the local distribution of AAV-mediated gene expression in the retina.
Regarding the side comment: eGFP is indeed expressed in the cells of the RPE after AAV transduction, albeit to distinct extents. We refer to that issue in the results section and have prepared a figure (new Figure S11) illustrating eGFP-mediated epifluorescence in the RPE layers.

6) Legend to Fig. S1: It appears that a string of the legend, which included the last part of the description of panel F as well as the beginning of the description of panel G, is missing. Please check.
→ There was no mistake during the creation of the .pdf document, but obviously, our wording was misleading. We improved this section by:
*“**(F)** In a male cell line, targeting the porcine USH1C locus was less efficient than in the female cell line that was used for generating USH1C pigs. **(G)** Sanger sequencing of the modified clones confirmed correct transition from the humanized fragment to the porcine sequence in USH1C intron 2.”*

7) Page 8: it appears that the "Fig. 8SD, E" is erroneously referenced after the sentence "USH1C_b splice variants were found at low level in general, but clearly indicated a lack of exon 27 in all transcripts and alternative splicing of exon 20 (Figure 8SD, E)." Please check.
→ We have corrected this typo and now correctly refer to Figure S10D, E.

**Referee #2 (Remarks for Author):**
I think this is a nice study and the paper is much improved now. The revised manuscript has adequately addressed my concerns. I think it is suitable for publication.
→ We thank referee #2 for this nice and clear statement.

**Referee #3 (Remarks for Author):**
Manuscript Number: EMM-2021-14817-V2
Title: Early Disruption of Photoreceptor Cell Architecture and Loss of Vision in a Humanized Pig Model of Usher Syndrome
Corresponding Author: Dr. Klymiuk
In this revised version, the authors have significantly improved the quality of the manuscript and have satisfactorily responded to my points. The authors now provide new data generated by sub-retinal injection of AAV-harmonin_a1 vectors that show significant restoration of retinal function in two USH1C pigs (Fig 7D and Fig EV5). Overall, the revised manuscript shows more convincingly a translational clinical potential of their USH1C pig model.
→ We thank referee #3 for the positive judgement.

I only have few minor comments:
1) The last sentence of the abstract is not clear and needs to be rewritten. Please change mRNA therapy that could be confusing by AAV-gene therapy or gene therapy.

→ the comment on mRNA therapy meets referee #1 ´s pt 4. We now use the term "AAV-based delivery of harmonin", as suggested by referee #1 throughout the manuscript. For facilitating better understanding, the last sentence of the abstract was modified into *"AAV-based delivery of harmonin into the eye of USH1C pigs indicated therapeutic efficacy in vivo."*

2) I think it is important to precise somewhere in page 10 of Results that the modification of the porcine USH1C gene in exon 2 (Fig 1C) will affect the three isoforms of harmonin.

→ We thank the referee for pointing at this missing information. We have now included the sentence *"For the integration of exon 2 in the major splice variants USH1C_a, _b, and _c, the chosen mutation will affect all relevant USH1C transcripts and its disruption was, therefore, proposed to cause the full spectrum of USH."* in the introduction section. We also referred to this point in the first paragraph of the chapter *"USH1C pigs facilitate testing of therapeutic approaches in vivo and in vivo"* by introducing the sentence *"The defined genetic cause of the model, the disruption of exon 2, affects all relevant USH1C splice forms and, therefore, facilitates the testing of a broad spectrum of treatment options."*

3) Problem: Fig 7D left graph is identical to Fig EV5D. In Fig EV5D, I suggest to select another ERG recording from pig # 10056. Alternatively, I suggest to replace the right panel 7D by the two panels of EV5B. Also indicate the numbering of the 2 pigs # 10056 and # 10054 in 7D. In EV5 then show only panels A and C.

→ We followed the referee´s suggestion and implemented the light-adapted ERG of both AAV-treated USH1C pigs in Figure 7D and show only dark-adapted ERG and the quantification of ERG data in Figure 5. The distinct ages at time points of injection and the distinct post-operative observation periods are highlighted in the legend of Figure 7D.

4) Page 21, Legend of Fig7A last sentence, (representative images from 1 pig - remove s)

→ removed; thank you for helping to correct this typo.

## 2nd Revision - Editorial Decision 25th Jan 2022

25th Jan 2022

Dear Dr. Klymiuk,

Thank you for the submission of your revised manuscript to EMBO Molecular Medicine. We have now received the enclosed report from referee #1 who re-reviewed your manuscript. As you will see, this referee appreciates that you have addressed most concerns, but still raises a remaining issue that should be addressed before we can accept your manuscript. Therefore, please clarify the genotype of the pigs listed in Appendix Table S1 ("unclear").

Furthermore, please also address the following editorial points:
- Please remove the coloured text.
- In the material and methods and in the checklist, and in relation to human skin biopsies, please include a statement confirming that informed consent was obtained from all subjects and that the experiments conformed to the principles set out in the WMA Declaration of Helsinki and the Department of Health and Human Services Belmont Report.
- Please indicate in the figures or in their legends the exact p values, not a range, including for non-significant p values. You may provide exact p values as an additional table in the Appendix file if you prefer.
- Thank you for providing a synopsis text. I added minor modification to fit our style and format, please let me know if you agree with the following:

"The full phenotypic spectrum of Usher Syndrome is reflected in a pig model carrying a patient-specific mutation after partial humanization of the porcine USH1C gene. Retinal function in USH1C pigs was improved when harmonin expression was reconstituted by AAV-mediated gene therapy.
• Partial humanization of the porcine USH1C gene was facilitated by the high degree of sequence conservation in the N-terminal region of harmonin.
• Vision loss within the first year of life in USH1C pigs was consistently indicated by behavior tests, clinical examination and morphological analysis.
• Early onset of vision loss was correlated to disrupted photoreceptor cell architecture.
• Ciliopathy mechanisms and their therapeutic correction were investigated in primary cells of USH1C individuals.
• Local application and therapeutic efficacy of AAV-mediated treatments was examined in USH1C pigs in vivo."

Please remove the synopsis from the main manuscript text and upload it as an independent file.

I look forward to receiving your revised manuscript.

With kind regards,

Lise Roth

Lise Roth, PhD

Editor

EMBO Molecular Medicine

To submit your manuscript, please follow this link:

in ot A i e

The system will prompt you to fill in your funding and payment information. This will allow Wiley to send you a quote for the article processing charge (APC) in case of acceptance. This quote takes into account any reduction or fee waivers that you may be eligible for. Authors do not need to pay any fees before their manuscript is accepted and transferred to our publisher.

***** Reviewer's comments *****

Referee #1 (Remarks for Author):

The authors have addressed most of my concern. Yet, further clarification on the genotype of the pigs used in the studies is required. Indeed, over the manuscript the authors refer to two types of mutations found in the animals: "One constituted the c.C91T / p.R31X mutation while the other was a large genomic disruption, deleting exon 2 of the USH1C gene" (results section; page 6-7). However, in the new Appendix Table S1, most of the homo animals have a genotype defined as "unclear". Please clarify what "unclear" means. Do these pigs carry an additional type of pathogenic mutation which could be associated with USH1C phenotype development?

***** Reviewer's comments *****

**Referee #1 (Remarks for Author):**

The authors have addressed most of my concern. Yet, further clarification on the genotype of the pigs used in the studies is required. Indeed, over the manuscript the authors refer to two types of mutations found in the animals: "One constituted the c.C91T / p.R31X mutation while the other was a large genomic disruption, deleting exon 2 of the USH1C gene" (results section; page 6-7). However, in the new Appendix Table S1, most of the homo animals have a genotype defined as "unclear". Please clarify what "unclear" means. Do these pigs carry an additional type of pathogenic mutation which could be associated with USH1C phenotype development?

→ The terminus “unclear” referred to the difficulties to discriminate a R31X /R31X allelic constellation from a R31X/del constellation in the offspring of a R31X/del x R31X/WT matings by conventional end-point PCR. We agree, however, with the referee that the characterization of these animals as homozygous USH1C KO is not satisfying. We have therefore clarified the exact genotype in all experimental animals by the qPCR-based approached of determining the number of R31X alleles that we have already documented in the revised version of the manuscript.

9th Feb 2022

Dear Dr. Klymiuk,

Thank you for the submission of your revised manuscript to EMBO Molecular Medicine. We have consulted referee #1 who approved your new Table S1. I am therefore pleased to inform you that your manuscript is accepted for publication and is now being sent to our publisher to be included in the next available issue of EMBO Molecular Medicine.

Please read below for additional IMPORTANT information regarding your article, its publication and the production process.

Congratulations on your interesting work!

With kind regards,

Lise Roth

Lise Roth, Ph.D
Scientific Editor
EMBO Molecular Medicine

Follow us on Twitter @EmboMolMed
Sign up for eTOCs at embopress.org/alertsfeeds

EMBO PRESS

YOU MUST COMPLETE ALL CELLS WITH A PINK BACKGROUND

PLEASE NOTE THAT THIS CHECKLIST WILL BE PUBLISHED ALONGSIDE YOUR PAPER

Corresponding Author Name: Klymiuk, Nikolai
Journal Submitted to: EMBO Molecular Medicine
Manuscript Number: EMM-2021-14817

USEFUL LINKS FOR COMPLETING THIS FORM

http://www.antibodypedia.com
http://1degreebio.org
http://www.equator-network.org/reporting-guidelines/improving-bioscience-research-repor
http://grants.nih.gov/grants/olaw/olaw.htm
http://www.mrc.ac.uk/Ourresearch/Ethicsresearchguidance/Useofanimals/index.htm
http://ClinicalTrials.gov
http://www.consort-statement.org
http://www.consort-statement.org/checklists/view/32-consort/66-title
http://www.equator-network.org/reporting-guidelines/reporting-recommendations-for-tumo
http://datadryad.org
http://figshare.com
http://www.ncbi.nlm.nih.gov/gap
http://www.ebi.ac.uk/ega
http://biomodels.net/
http://biomodels.net/miriam/
http://jjj.biochem.sun.ac.za
https://osp.od.nih.gov/biosafety-biosecurity-and-emerging-biotechnology/
http://www.selectagents.gov/

## Reporting Checklist For Life Sciences Articles (Rev. June 2017)

This checklist is used to ensure good reporting standards and to improve the reproducibility of published results. These guidelines are consistent with the Principles and Guidelines for Reporting Preclinical Research issued by the NIH in 2014. Please follow the journal's authorship guidelines in preparing your manuscript.

### A- Figures
### 1. Data
### The data shown in figures should satisfy the following conditions:

- the data were obtained and processed according to the field's best practice and are presented to reflect the results of the experiments in an accurate and unbiased manner.
- figure panels include only data points, measurements or observations that can be compared to each other in a scientifically meaningful way.
- graphs include clearly labeled error bars for independent experiments and sample sizes. Unless justified, error bars should not be shown for technical replicates.
- if n< 5, the individual data points from each experiment should be plotted and any statistical test employed should be justified
- Source Data should be included to report the data underlying graphs. Please follow the guidelines set out in the author ship guidelines on Data Presentation.

### 2. Captions
### Each figure caption should contain the following information, for each panel where they are relevant:

- a specification of the experimental system investigated (eg cell line, species name).
- the assay(s) and method(s) used to carry out the reported observations and measurements
- an explicit mention of the biological and chemical entity(ies) that are being measured.
- an explicit mention of the biological and chemical entity(ies) that are altered/varied/perturbed in a controlled manner.

- the exact sample size (n) for each experimental group/condition, given as a number, not a range;
- a description of the sample collection allowing the reader to understand whether the samples represent technical or biological replicates (including how many animals, litters, cultures, etc.).
- a statement of how many times the experiment shown was independently replicated in the laboratory.
- definitions of statistical methods and measures:
  - common tests, such as t-test (please specify whether paired vs. unpaired), simple $\chi 2$ tests, Wilcoxon and Mann-Whitney tests, can be unambiguously identified by name only, but more complex techniques should be described in the methods section;
  - are tests one-sided or two-sided?
  - are there adjustments for multiple comparisons?
  - exact statistical test results, e.g., P values = x but not P values < x;
  - definition of 'center values' as median or average;
  - definition of error bars as s.d. or s.e.m.

Any descriptions too long for the figure legend should be included in the methods section and/or with the source data.

**In the pink boxes below, please ensure that the answers to the following questions are reported in the manuscript itself. Every question should be answered. If the question is not relevant to your research, please write NA (non applicable). We encourage you to include a specific subsection in the methods section for statistics, reagents, animal models and human subjects.**

### B- Statistics and general methods

Please fill out these boxes (Do not worry if you cannot see all your text once you press return)

| | |
|---|---|
| 1.a. How was the sample size chosen to ensure adequate power to detect a pre-specified effect size? | NA |
| 1.b. For animal studies, include a statement about sample size estimate even if no statistical methods were used. | For the high production effort of large animals, sample size was determined by numbers of available animals, e.g. animals within a litter. In case of 2 litters at the same time, animals were combined in one cohort. In the case of naturally produced litters, WT littermates were used as controls to minimize genetic background variability. In the case of litters arising from nuclear transfer, age-matched WT controls were used. |
| 2. Describe inclusion/exclusion criteria if samples or animals were excluded from the analysis. Were the criteria pre-established? | We excluded animal experiments only in the behavior tests, mainly because of limited compliance / motivation to run the course. Occasionally, animals were excluded due to temporary illness (e.g. lameness). Exclusion criteria are described in the supplemental Materials & Methods section in detail. |
| 3. Were any steps taken to minimize the effects of subjective bias when allocating animals/samples to treatment (e.g. randomization procedure)? If yes, please describe. | In behavior tests, randomized orders of the experimental animals were used for each test run. For anesthesia, intervention, and for sampling, storage and processing of tissue we followed standard operating procedures. For cellular experiments we used >6 technical replicates. |
| For animal studies, include a statement about randomization even if no randomization was used. | Groups were defined by genotyping. We did not perform systematic treatment studies, so no randomization was done in the gene therapy experiments. |
| 4.a. Were any steps taken to minimize the effects of subjective bias during group allocation or/and when assessing results (e.g. blinding of the investigator)? If yes please describe. | In ABR, the experimentators were not aware of the genotype of the investigated animals. In the ERG examination of the animals after receiving gene therapy, the experimentator was not aware of which eye was AAV-treated and which was sham-treated. Blinding was, not possible for the ERG / OCT measurements and the behavior test on OCT and WT animals. |
| 4.b. For animal studies, include a statement about blinding even if no blinding was done | In ABR, the experimentators were not aware of the genotype of the investigated animals. In the ERG examination of the animals after receiving gene therapy, the experimentator was not aware of which eye was AAV-treated and which was sham-treated. Blinding was, not possible for the ERG / OCT measurements and the behavior test on OCT and WT animals. |
| 5. For every figure, are statistical tests justified as appropriate? | Yes, we have used appropriate tests for statistical examination. Test method and signifances are given in each figure text or in the figure. |
| Do the data meet the assumptions of the tests (e.g., normal distribution)? Describe any methods used to assess it. | for behavior tests we used most conservative Mann-Whitney U test (Figures 2, S4), most prominent data were (duration, contacts) were for consistency and normality (Figure S6). For tests on cells, we used Kolmogorov-Smirnov test to check for cumulative distribution. All other statistically examined experiments were performed with two-tailed Student´s t-test, as independent samples were examined. |

| | |
|---|---|
| Is there an estimate of variation within each group of data? | We indicated mean value +/- standard deviation and/or mean value +/- standard error for the statistically examined experiments. Whereever possible we depicted also each measurement as dot in the figure. |
| Is the variance similar between the groups that are being statistically compared? | we observed more variability in the USH1C groups, particularly in the behavior tests. We interprete this as a consequence of individual disease progression and/or the influence of vestibular dysfunction in locomotion resulting in individual adaptation in the respective animals. |

### C- Reagents

| | |
|---|---|
| 6. To show that antibodies were profiled for use in the system under study (assay and species), provide a citation, catalog number and/or clone number, supplementary information or reference to an antibody validation profile. e.g., Antibodypedia (see link list at top right), 1DegreeBio (see link list at top right). | antibody are defined in Supplemental Materials & Methods (pg9./10), each of them either by reference to literature in the case of non-commercial AB and with catalog number and supplier for commercially available AB. |
| 7. Identify the source of cell lines and report if they were recently authenticated (e.g., by STR profiling) and tested for mycoplasma contamination. | we did not use cell lines, but isolated primary cells. We did not examine mycoplasma contamination. |

* for all hyperlinks, please see the table at the top right of the document

### D- Animal Models

| | |
|---|---|
| 8. Report species, strain, gender, age of animals and genetic modification status where applicable. Please detail housing and husbandry conditions and the source of animals. | we report on the generation of a new animals model, created by SCNT from pig primary cells. Resulting animals were housed, raised and propagated at two certified institutions (CiMM - Center of innovative Medial Models, LMU Munich and PigMod Center - IAPG Libechov). Details are described in the Supplemental Materials & Methods. |
| 9. For experiments involving live vertebrates, include a statement of compliance with ethical regulations and identify the committee(s) approving the experiments. | Animal work were conducted under the rules and supervision of the responsible authorities in the above mentioned facilities. Animal experiments were approved at the responsible authorities. Reference numbers of approvals are given in the manuscript under the specific "regulatory statement" section. |
| 10. We recommend consulting the ARRIVE guidelines (see link list at top right) (PLoS Biol. 8(6), e1000412, 2010) to ensure that other relevant aspects of animal studies are adequately reported. See author guidelines, under 'Reporting Guidelines'. See also: NIH (see link list at top right) and MRC (see link list at top right) recommendations. Please confirm compliance. | National law and permissions for animal experiments are following European guidelines, concerted with ARRIVE guidelines. Housing in CiMM and PigMod are following ARRIVE guidelines. |

### E- Human Subjects

| | |
|---|---|
| 11. Identify the committee(s) approving the study protocol. | Human primary cells were provided by University of Tuebingen, Germany, work on the cells was carried under approval of the ethics committee of the Landesärztekammer Rheinland-Pfalz. The reference number is given in the Regulatory Statement of the manuscript. |
| 12. Include a statement confirming that informed consent was obtained from all subjects and that the experiments conformed to the principles set out in the WMA Declaration of Helsinki and the Department of Health and Human Services Belmont Report. | Informed consent was obtained from all subjects providing skin biopsies. The experiments on human fibroblasts conformed to the principles set out in the WMA Declaration of Helsinki and the Department of Health and Human Services Belmont Report. |
| 13. For publication of patient photos, include a statement confirming that consent to publish was obtained. | NA |
| 14. Report any restrictions on the availability (and/or on the use) of human data or samples. | NA |
| 15. Report the clinical trial registration number (at ClinicalTrials.gov or equivalent), where applicable. | NA |
| 16. For phase II and III randomized controlled trials, please refer to the CONSORT flow diagram (see link list at top right) and submit the CONSORT checklist (see link list at top right) with your submission. See author guidelines, under 'Reporting Guidelines'. Please confirm you have submitted this list. | NA |
| 17. For tumor marker prognostic studies, we recommend that you follow the REMARK reporting guidelines (see link list at top right). See author guidelines, under 'Reporting Guidelines'. Please confirm you have followed these guidelines. | NA |

### F- Data Accessibility

| | |
|---|---|
| 18: Provide a "Data Availability" section at the end of the Materials & Methods, listing the accession codes for data generated in this study and deposited in a public database (e.g. RNA-Seq data: Gene Expression Omnibus GSE39462, Proteomics data: PRIDE PXD000208 etc.) Please refer to our author guidelines for 'Data Deposition'.<br><br>Data deposition in a public repository is mandatory for:<br>a. Protein, DNA and RNA sequences<br>b. Macromolecular structures<br>c. Crystallographic data for small molecules<br>d. Functional genomics data<br>e. Proteomics and molecular interactions | NA - we did not perform analysis macromolecular structures, small molecule crystallography, functional genomics, proteomics or transcriptomics. Molecular analysis on transcripts and proteins were based on publicly available information. AB and primers are characterized in detail in the Supplemental Materials & Methods section. |
| 19. Deposition is strongly recommended for any datasets that are central and integral to the study; please consider the journal's data policy. If no structured public repository exists for a given data type, we encourage the provision of datasets in the manuscript as a Supplementary Document (see author guidelines under 'Expanded View' or in unstructured repositories such as Dryad (see link list at top right) or Figshare (see link list at top right). | Information is given in the Supplementary Materials & Methods section. |
| 20. Access to human clinical and genomic datasets should be provided with as few restrictions as possible while respecting ethical obligations to the patients and relevant medical and legal issues. If practically possible and compatible with the individual consent agreement used in the study, such data should be deposited in one of the major public access-controlled repositories such as dbGAP (see link list at top right) or EGA (see link list at top right). | NA |
| 21. Computational models that are central and integral to a study should be shared without restrictions and provided in a machine-readable form. The relevant accession numbers or links should be provided. When possible, standardized format (SBML, CellML) should be used instead of scripts (e.g. MATLAB). Authors are strongly encouraged to follow the MIRIAM guidelines (see link list at top right) and deposit their model in a public database such as Biomodels (see link list at top right) or JWS Online (see link list at top right). If computer source code is provided with the paper, it should be deposited in a public repository or included in supplementary information. | NA |

### G- Dual use research of concern

| | |
|---|---|
| 22. Could your study fall under dual use research restrictions? Please check biosecurity documents (see link list at top right) and list of select agents and toxins (APHIS/CDC) (see link list at top right). According to our biosecurity guidelines, provide a statement only if it could. | No |
